# Identifying and analyzing different cancer subtypes using RNA-seq data of blood platelets

**DOI:** 10.18632/oncotarget.20903

**Published:** 2017-09-15

**Authors:** Yu-Hang Zhang, Tao Huang, Lei Chen, YaoChen Xu, Yu Hu, Lan-Dian Hu, Yudong Cai, Xiangyin Kong

**Affiliations:** ^1^ Department of General Surgery, Shanghai Jiao Tong University Affiliated Sixth People's Hospital, Shanghai 200233, People's Republic of China; ^2^ Institute of Health Sciences, Shanghai Institutes for Biological Sciences, Chinese Academy of Sciences, University of Chinese Academy of Sciences, Shanghai 200031, People's Republic of China; ^3^ School of Life Sciences, Shanghai University, Shanghai 200444, People's Republic of China; ^4^ College of Information Engineering, Shanghai Maritime University, Shanghai 201306, People's Republic of China; ^5^ Institute of Biochemistry and Cell Biology, Shanghai Institutes for Biological Sciences, Chinese Academy of Sciences, Shanghai 200031, People's Republic of China

**Keywords:** cancer detection, liquid biopsy, RNA-seq data, support vector machine, maximum relevance minimum redundancy

## Abstract

Detection and diagnosis of cancer are especially important for early prevention and effective treatments. Traditional methods of cancer detection are usually time-consuming and expensive. Liquid biopsy, a newly proposed noninvasive detection approach, can promote the accuracy and decrease the cost of detection according to a personalized expression profile. However, few studies have been performed to analyze this type of data, which can promote more effective methods for detection of different cancer subtypes. In this study, we applied some reliable machine learning algorithms to analyze data retrieved from patients who had one of six cancer subtypes (breast cancer, colorectal cancer, glioblastoma, hepatobiliary cancer, lung cancer and pancreatic cancer) as well as healthy persons. Quantitative gene expression profiles were used to encode each sample. Then, they were analyzed by the maximum relevance minimum redundancy method. Two feature lists were obtained in which genes were ranked rigorously. The incremental feature selection method was applied to the mRMR feature list to extract the optimal feature subset, which can be used in the support vector machine algorithm to determine the best performance for the detection of cancer subtypes and healthy controls. The ten-fold cross-validation for the constructed optimal classification model yielded an overall accuracy of 0.751. On the other hand, we extracted the top eighteen features (genes), including TTN, RHOH, RPS20, TRBC2, in another feature list, the MaxRel feature list, and performed a detailed analysis of them. The results indicated that these genes could be important biomarkers for discriminating different cancer subtypes and healthy controls.

## INTRODUCTION

Cancer has generally been regarded as a general term to describe a group of diseases associated with abnormal cell growth with invasive and metastatic characteristics [1–3]. Based on statistics from the WHO, every year, more than 8.2 million people die from cancer, accounting for approximately 13% of deaths worldwide, indicating that cancer is one of the most threatening diseases in the world [4, 5]. According to a prediction of the WHO, in the next two decades, the incidence of cancer may be elevated by more than 70% [5]. Therefore, it is urgent to study the biological foundations of cancer and modify clinical treatment strategies [6]. However, more than 100 types of cancer have been identified, each of which need to be diagnosed and treated specifically [5]. Considering the complexity of cancer diagnosis and treatment, it is quite important to establish a convenient and effective method for the early detection and identification of various cancer subtypes.

Traditionally, detection and identification of cancers relied on three basic groups of testing methods: lab tests, imaging procedures and biopsies [7, 8]. Lab tests mainly pay attention to specific substances in the body and generally involve the detection of body fluids, including blood, urine, cerebrospinal fluid (CSF), and so on [9–11]. However, lab tests reflect the overall conditions of the body with the use of only a few markers for tumor screening, such as carcino-embryonic antigen, CEA and alpha fetoprotein, AFP [10]. Doctors cannot diagnose cancer only based on lab tests. For further detection, imaging procedures, including CT scan, nuclear scan, ultrasound, MRI and X-rays, are used [12–14]. With the help of such medical apparatuses, doctors can see deeper into the body, which may simplify the diagnosis of cancer. However, most screening is expensive and has potential pathogenic effects, though screening may be quite safe at normal doses. Such characteristics may impose restrictions on large-scale screening of cancer patients. Medical imaging can only be applied to patients with certain clinical symptoms or tumor markers identified by lab tests for further identification and classification. Biopsies have been widely regarded as the gold standard for tumor diagnosis. With a needle, an endoscope, or during surgery, doctors directly withdraw tissue or fluid from patients for further pathological diagnosis [15–17]. Although such testing methods can obtain accurate pathological information from the patients or the tumor itself for correct diagnosis, as an invasive detection method, it not only can be quite expensive and time-consuming but can also have a risk of infection [18–20]. Tumor patients with certain infections may not be suitable for such detection.

Recently, gene detection has been introduced for the detection and diagnosis of tumors. Based on the genetic characteristics of tumor cells, people can precisely classify tumors (even those with similar clinical symptoms) into different molecular subtypes, which can be treated by appropriate therapeutic strategies [21]. However, detection relies on tumor tissues, which can only be obtained by invasive methods, such as biopsies, which are unsuitable for large-scale detection and early screening. To solve this problem, a new concept, liquid biopsy, has been presented [16, 22–24]. Liquid biopsy is a specific detection method that relies on the sampling and analysis of non-solid tissues, including blood, lymphatic fluid and CSF [25]. Unlike traditional biopsy, such a detection system is nearly non-invasive, with comparable accuracy [22, 24]. The combination of gene detection and liquid biopsies provides us with a new effective tool for accurate and non-invasive detection of tumors. In addition, it is suitable for large-scale detection and early screening. However, to apply such effective methods for tumor diagnosis, identification of effective markers turn out to be the premise problem for further development of liquid biopsy.

Based on multi-omics data, various approaches have been presented to identify and distinguish different tumor subtypes. In 2015, Zhang *et al*. reported an effective computational method to classify ten types of major cancer subtypes that threaten human health by reverse phase protein array profiles, implying the availability and feasibility of tumor detection by protein profiling [26]. Further, late in 2016, Zhang *et al*. presented a systematic analysis algorithm that contributes to the classification of cancers based on the copy number variation (CNV) landscape, confirming that the CNV landscape may also be an effective detection index for tumor classification [27]. Apart from such an analysis at the genomic and proteomic level, Best *et al.* reported an effective method to distinguish cancer subtypes solely based on RNA-seq results of tumor-educated platelets, a functional blood component that can be easily obtained by liquid biopsy [28]. Tumor-educated platelets contain specific pre-mRNAs of the bone marrow, spliced circulating mRNAs of primary and metastatic tumors, and specific spliced mRNAs of the platelets themselves induced by the tumor microenvironment, making tumor-educated platelets a perfect source for liquid biopsy. Such fundamental research achievements confirmed that the combination of genetic characteristics (either DNA-seq or RNA-seq results) and liquid biopsy might accomplish non-invasive, early detection and identification of different tumor subtypes. However, many markers and genes are redundant, and the genes that can be detected for diagnosis in liquid biopsy are limited [28]. Therefore, it is urgent to provide a computational method to analyze such data, thereby screening core and aberrantly expressed genes for further detection.

In this study, based on the RNA-seq results of tumor-educated platelets, we applied computational methods to screen core mRNA markers that can distinguish cancer subtypes from healthy controls. The gene expression profiles of blood from patients who had one of six cancer subtypes and healthy persons were analyzed by maximum relevance minimum redundancy (mRMR) [29]. Upon further analysis of the feature lists yielded by the mRMR method, eighteen important genes were extracted that may be essential biomarkers for the classification of cancer subtypes and healthy controls. In addition, an optimal classification model using a support vector machine (SVM) algorithm [30, 31] as the classifier was built, which provided good performance with an overall accuracy of 0.751 for diagnosing different cancer types and healthy controls.

## RESULTS

### Results of the mRMR method

In this study, each patient or healthy sample was represented by 13,445 features as described in Section “Dataset and feature construction”, each of which indicates the expression level of some gene. To analyze them, the mRMR method was employed. According to the relevance between features and targets, all features were ordered in the MaxRel feature list, in which features with high relevance to targets obtained high ranks. In addition, another feature list, mRMR feature list, was also yielded by the mRMR method by further considering the redundancies between features. These two lists are provided in Supplementary Table 1 and 2, respectively.

### Results of the IFS method

The IFS method was applied to the mRMR feature list yielded by the mRMR method to identify optimal features for classification. In this method, several feature sets were constructed, which consisted of some first features in the mRMR feature list. Then, for each feature set, the SVM was executed on the dataset, in which samples were represented by feature in the set. However, testing all of the possible feature subsets would take much time due to our limited computational power because 13,445 features were used in this study. In view of this, we designed an IFS method that contained two stages. In the first stage, we only tested some special feature subsets to determine the possible range of optimal features. In the second stage, all of the feature subsets in the possible range were tested to identify the optimal feature subset.

**Figure 1:**
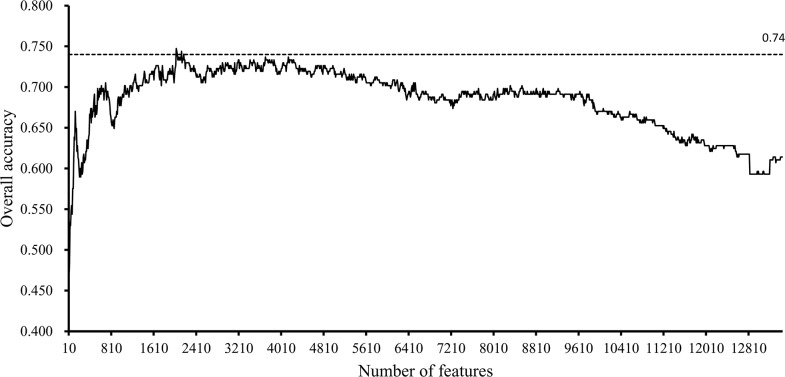
IFS-curves for the results yielded in the first stage of the IFS method The Y-axis represents the overall accuracy, and the X-axis represents the number of features used for classification. The high overall accuracies (no less than 0.740) all cluster between 2000 and 2200.

In the first stage, we tested the feature subsets F_*i*_, where *i* is a multiple of ten, *i.e.*, the numbers of features in these subsets were multiples of ten. For each of these feature subsets, the SVM was executed on all samples that were represented by features in this subset, with its performance evaluated by ten-fold cross-validation. The predicted results were counted as accuracies and specificity, as mentioned in Section “Measurements”. After all of these feature subsets had been tested, several accuracies and specificities were obtained, which are provided in Supplementary Table 3 and 4. Because the overall accuracy *TACC* was selected as the major measurement, we plotted a curve, namely, an IFS-curve, with *TACC* as the Y-axis and the number of features as the X-axis, which is shown in Figure 1, to extract the feature subset that can yield the best performance for the SVM. It can be observed that the IFS-curve first follows a sharp increasing trend and reaches the maximum overall accuracy (0.747) when 2030 features were used before becoming stable and following a slow decreasing trend. The high overall accuracies (no less than 0.740) all clustered at approximately 2100. Thus, we believed that the possible range of optimal features was between 2000 and 2200.

**Figure 2:**
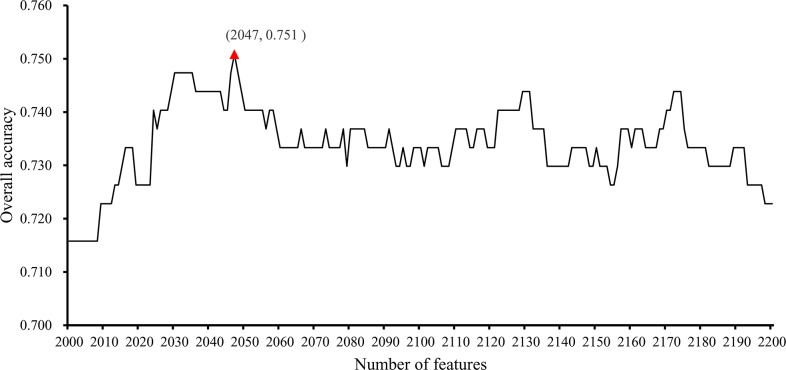
IFS-curves for the results yielded in the second stage of the IFS method The Y-axis represents the overall accuracy, and the X-axis represents the number of features used for classification. The highest overall accuracy was 0.751 when 2047 features were used.

**Figure 3:**
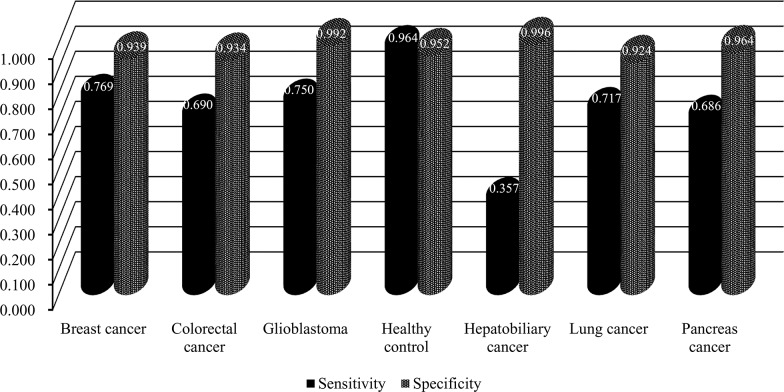
The performance of the optimal classification model evaluated by ten-fold cross-validation

In the second stage, we further tested the feature subsets F_*i*_ with 2000≤*i*≤2200. The obtained accuracies and specificities mentioned in Section “Measurements” are provided in Supplementary Table 5 and 6. For ease of observation, we plotted a curve with the overall accuracy (*TACC*) as the Y-axis the number of features as the X-axis, as shown in Figure 2. We can see that the highest *TACC* was 0.751 when the top 2,047 features in the mRMR feature list were used for classification. Thus, these features were deemed to be optimal features and comprised the optimal feature subset. By using these optimal features, an optimal classification model was built. The detailed performance of this model is shown in Figure 3. It can be seen that the specificity for each class is quite high (more than 0.920) and the prediction accuracy for each class (i.e., sensitivity) is quite high, except for the accuracy for hepatobiliary cancer. The possible reason for the low accuracy of hepatobiliary cancer may be the small size of this class, which only contained fourteen samples, while the other classes contained at least 35 samples (more than twice as many samples than those available for hepatobiliary cancer).

### Comparison with commercial cancer detection panels

There are already some commercial cancer detection panels. Here, we collected cancer panel genes from the following seven commercial cancer detection panels: (I) CancerNext (http://www.ambrygen.com/tests/cancernext), (II) CancerNextExpanded (http://www.ambrygen.com/tests/cancernext-expanded), (III) CloudHealth (http://en.chgenomics.com/products/hereditary), (IV) GeneDx (https://www.genedx.com/test-catalog/available-tests/comprehensive-cancer-panel/), (V) Illumina (https://www.illumina.com.cn/products/by-type/clinical-research-products/trusight-rna-pan-cancer.html), (VI) NanoString (https://www.nanostring.com/products/gene-expression-panels/hallmarks-cancer-gene-expression-panel-collection/pancancer-pathways-panel), (VII) xGen (https://www.idtdna.com/pages/products/nextgen/target-capture/xgen-lockdown-panels/xgen-pan-cancer-panel). The retrieved genes from these seven panels were provided in Supplementary Table 7.

Using the same procedures for building the optimal classification model mentioned in Section “Results of the IFS method”, genes retrieved from each panels can yield an optimal classification model. The performance of these classification models are listed in Table 1, from which we can see that the performance of these models were much inferior to the proposed model.

**Table 1:** The performance of the optimal classification models using different reference gene sets

Reference gene set	TACC
This study	0.751
Genes in CancerNext	0.407
Genes in CancerNextExpanded	0.463
Genes in CloudHealth	0.421
Genes in GeneDx	0.400
Genes in Illumina	0.656
Genes in NanoString	0.618
Genes in xGen	0.519

### Important genes

For the MaxRel feature list yielded by the mRMR method, extensive investigation of some of the top features may lead to novel biomarkers for distinguishing different cancer patients. In the MaxRel feature list, each feature was measured by an MI value. A feature with a high MI value indicates that it is quite important. Thus, we set a threshold of 0.360 to select important features, *i.e.*, features with MI values larger than 0.360 were extracted, corresponding to the eighteen genes listed in Table 2. We also investigated ranks of these eighteen genes in the mRMR feature list and listed them in the last column of Table 2. It can be seen that the maximum rank was 122, indicating that these important eighteen genes were all in the optimal feature subset that consisted of the top 2,047 features in the mRMR feature list. It can partly prove that these eighteen genes were quite essential for classification of six cancer subtypes and healthy samples. In the following section, these features were extensively analyzed to uncover the differences of the biological processes and molecular functions between the six cancer subtypes and healthy samples.

**Table 2:** The top 18 features in the MaxRel feature list

Order	Feature name	Gene name	Description	MI value	Rank in the mRMR feature list
1	ENSG00000155657	TTN	Titin	0.416	1
2	ENSG00000008988	RPS20	Ribosomal Protein S20	0.407	13
3	ENSG00000177600	RPLP2	Ribosomal Protein Lateral Stalk Subunit P2	0.405	6
4	ENSG00000211772	TRBC2	T Cell Receptor Beta Constant 2	0.396	19
5	ENSG00000168028	RPSA	Ribosomal Protein SA	0.393	35
6	ENSG00000142534	RPS11	Ribosomal Protein S11	0.384	64
7	ENSG00000142676	RPL11	Ribosomal Protein L11	0.381	48
8	ENSG00000105193	RPS16	Ribosomal Protein S16	0.380	57
9	ENSG00000160654	CD3G	CD3g Molecule	0.379	25
10	ENSG00000168421	RHOH	Ras Homolog Family Member H	0.373	3
11	ENSG00000139193	CD27	CD27 Molecule	0.369	8
12	ENSG00000131469	RPL27	Ribosomal Protein L27	0.368	106
13	ENSG00000163682	RPL9	Ribosomal Protein L9	0.368	86
14	ENSG00000071082	RPL31	Ribosomal Protein L31	0.367	78
15	ENSG00000149311	ATM	ATM Serine/Threonine Kinase	0.367	17
16	ENSG00000149806	FAU	FAU, Ubiquitin Like And Ribosomal Protein S30 Fusion	0.366	31
17	ENSG00000109475	RPL34	Ribosomal Protein L34	0.366	122
18	ENSG00000089009	RPL6	Ribosomal Protein L6	0.366	117

## DISCUSSION

**Figure 4:**
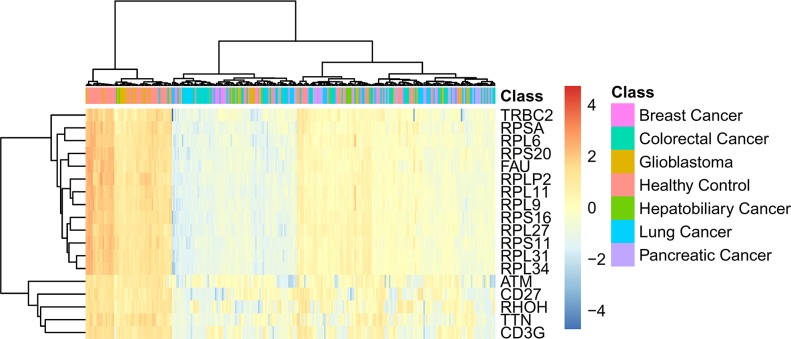
The heat map of all samples using the important eighteen genes

In Section “Important genes”, eighteen important genes, listed in Table 2, were extracted. These 18 genes are deemed to be important for distinguishing six cancer subtypes and healthy samples. Figure 4 shows the heat map of all samples using the important eighteen genes. It can be seen that the healthy samples were clearly clustered together and among the cancer samples, the Glioblastoma samples were most similar with healthy samples. Generally based on our results, we summarized two specific biological and functional characteristics of various functional genes have been predicted. First, various immune associated genes like CD3G have been predicted, indicating the distinctive expression pattern in tumor and normal tissues. During the tumorigenesis of various cancer subtypes, like breast cancer, colorectal cancer, glioblastoma, the immune system of patients have been confirmed to be systemically suppressed, especially in the tumor microenvironment (cancer adjacent tissues) [32–34]. Therefore, it is quite reasonable to predict genes that contributing to immune reaction as potential differentially expressed genes and biomarkers. Another specific characteristic turns out to be that glioblastoma as a brain cancer has the most similar expression pattern with normal patients based on liquid biopsy of blood platelet. Recent publications confirmed that during the initiation and progression of brain cancer like glioblastoma, the Blood Brain Barrier (BBB) acts as an effective protective screen, preventing the spread of characteristic biomarkers from the brain to the circulating system, resulting in the major liquid biopsy biomarkers that have been identified are mostly based on cerebrospinal fluid detection [35, 36]. Therefore, the expression profile of blood platelet from brain cancer patients and normal controls may appear to be the most similar, comparing to other cancer subtypes. Here, in our study, based on detailed expression profiling data, we successfully validated the similarity between the blood biopsy result of brain cancer patients and normal controls and further identified the potential biomarkers that can recognize brain cancer patients, confirming that similar as blood expression pattern of brain cancer patients with normal controls, there still remain potential biomarkers to reflect the tumorigenesis processes. As for elaborating biological processes that the important gene may participate in, the detailed analysis of each functional gene can be seen below.

**Figure 5:**
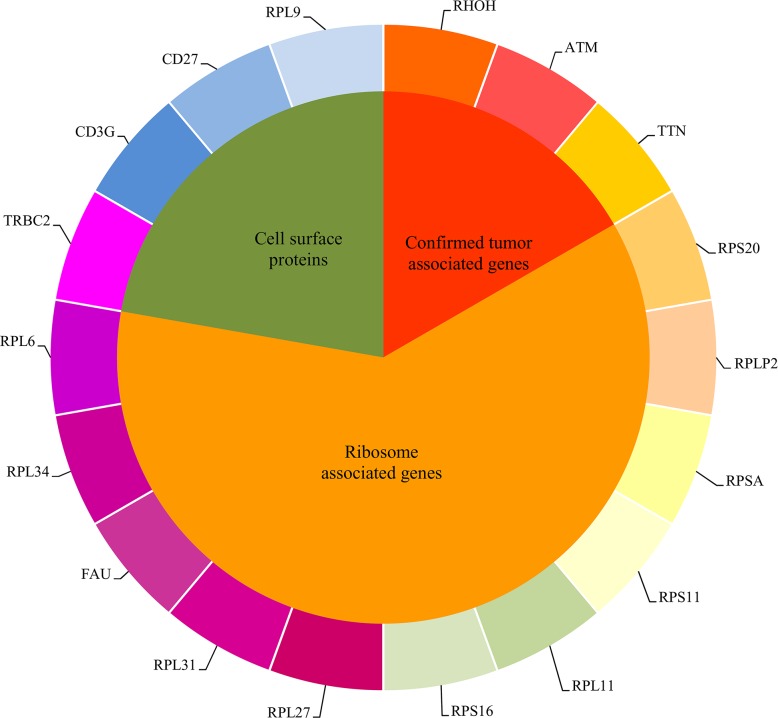
The eighteen important genes found in the MaxRel feature list were clustered into three groups

Based on recent publications, the specific functions of these genes and the specific biological processes that these genes participate in, it can be confirmed that these genes may form grouping standards for cancer subtype identification and differential diagnosis. For clarity, these eighteen genes were clustered into three groups, as shown in Figure 5. The following sections analyze the genes we extracted and divided into different groups one by one.

### Confirmed tumor associated genes

Among the eighteen genes, a specific oncogene, TTN (ENSG00000155657), has been regarded as a crucial marker for the distinction of six cancer subtypes and healthy controls. Encoding the protein Titin, this gene has been confirmed to contribute to platelet activation and cardiac conduction [37, 38]. This gene has also been reported to be a specific cancer-associated gene that can distinguish healthy controls from the other six subgroup of cancers, which all have been confirmed by recent publications [39–41]. Recent publications also confirmed that TTN might affect the composition of serum proteins, as it is expressed in hematopoietic cells, thus implying that TTN can be used as a potential marker in liquid biopsies [42]. Another gene, ENSG00000168421, is the tumor suppressor RHOH, a member of the Ras superfamily. Considering that this gene is expressed in hematopoietic cells, it is quite suitable for it to act as a liquid biopsy marker for the differential diagnosis of tumor [43, 44]. Although this gene has been confirmed to contribute to tumorigenesis, there are few reports on it. This implies that, currently, RHOH can only be used to differentiate between tumor samples and normal controls. ATM, Ataxia Telangiectasia Mutated serine/threonine kinase (ENSG00000149311), has been widely reported to contribute to the regulation of the cell cycle as a member of the PI3/PI4-kinase family [45, 46]. Based on recent publications, this tumor-associated gene contributes to abnormal proliferation and invasion of tumor cells in multiple tumor subtypes [47, 48]. Furthermore, a 2013 *Blood* report showed that ATM might participate in the secretion of exosomes during tumorigenesis and angiogenesis, confirming the possibility of its early detection by liquid biopsy [49]. Among our candidate subtypes, this gene has been confirmed to contribute to all six cancer subtypes, implying that this gene may be a functional marker to distinguish healthy samples from specific cancer subtypes [50–55].

### Ribosome associated genes

For a long time, ribosome associated genes which contribute to ribosome biogenesis have been confirmed to be a group of functional tumor associated genes regulating the proliferation rate of tumor cells [56, 57]. Among the important eighteen genes, some were ribosome associated genes. RPS20 (ENSG00000008988) has also been predicted to be a candidate biomarker. This gene has been reported to encode a ribosomal protein component of the 40S subunit [58, 59]. Different from genes that contribute to all six cancer subtypes, RPS20 has only been identified in limited cancer subtypes, including colorectal cancer and glioblastoma [60, 61]. Therefore, in our seven types of samples, this gene can distinguish colorectal cancer and glioblastoma from the other four cancer subtypes and healthy controls. Further research on this gene also confirmed that it can be identified in exosomes of colorectal carcinoma, which can be further detected by liquid biopsy, validating our prediction [62]. As another ribosome associated gene, RPSA (ENSG00000168028) may also be differentially expressed in our candidate seven groups. Based on recent publications, this gene has only been identified in colorectal cancer, lung cancer, esophageal squamous cancer and acute leukemia [63]. As for the six candidate cancer subtypes and healthy controls in our study, RPSA can distinguish colorectal cancer and lung cancer from the other subtypes [63, 64]. Similarly, another ribosome-associated gene, RPS11 (ENSG00000142534), has also been found to be a candidate biomarker. This gene also encodes a specific component of the 40S subunit. Recent publications have identified it in breast cancer, glioblastoma, lung cancer and colorectal cancer, allowing us to distinguish samples of those four cancers from hepatobiliary cancer samples and normal controls [60, 65, 66]. Similarly, as a homologue of RPS11 analyzed above, RPS16 (ENSG00000105193) was also listed as a candidate biomarker in our study. Like RPS11, this gene encodes a ribosomal protein that is a component of the 40S subunit. According to recent publications, various systematic diseases have been attributed to RPS16, including Diamond-Blackfan Anemia and cancer [67–69]. Furthermore, based on recent publications, only hepatobiliary cancer has been reported to be associated with abnormal functions of this gene, as detected in blood samples, implying that this gene can be a candidate liquid biopsy marker for hepatobiliary cancer [68]. Similar to RPS20, as analyzed above, all of the homologues of RPS20 can also be functional components of exosomes, implying the differentiated role of RPS20 and its homologues for the detection of cancer by liquid biopsy [70, 71].

ENSG00000177600, RPLP2, also has ribosome-associated functions. This gene encodes a component of the 60S subunit [72]. Based on recent publications, this gene has been identified in the blood component of only three tumor subtypes in our study: colorectal cancer, breast cancer and hepatobiliary cancer, indicating its potential contribution to pathological typing [73–75]. As another component of the 40S subunit, RPL11 (ENSG00000142676) encodes another sub-component of the ribosome 40S subunit. Based on recent publications, this gene has been confirmed to contribute to the RPL11-HDM2-p53 nucleolar stress response pathway, which is coupled with the Akt/mTORC1 signaling axis, implying its function during tumorigenesis [76]. However, based on recent findings, there is no direct evidence for RPL11 to contribute to one or a few specific cancer subtypes, implying that it can only differentiate between the normal control and the other six subtypes of cancers. Combined with its specific expression in exosomes, RPL11 may be an effective biomarker for tumor liquid biopsy, validating our prediction [77]. Similarly, RPL31 (ENSG00000071082), as a functional component of the 60S ribosome, has also been confirmed to be a candidate biomarker [78]. Until now, this gene has only been identified in four cancer subtypes: breast cancer, prostate cancer, pancreatic cancer and gastric carcinoma [69, 79–83]. Therefore, considering the specific expression profile of RPL31 in exosomes, it is quite reasonable to use this gene as a crucial standard for the further classification of different tumor subtypes, distinguishing breast cancer and pancreatic cancer from the other four specific cancer subtypes and healthy controls.

FAU, as a ubiquitin-like and ribosomal protein S30 fusion (ENSG00000149806), has been wildly reported to contribute to the biological processes related to Finkel-Biskis-Reilly (FBR)-murine sarcoma virus as a potential secretory protein [84, 85]. This gene has been confirmed to contribute to the initiation of breast cancer, implying that it may be a functional biomarker for the identification and differential diagnosis of breast cancer [86]. ENSG00000109475, RPL34, has also been predicted to be a candidate gene to distinguish between the six cancer subtypes and healthy control. As another ribosomal protein, this gene has only been identified in lung cancer and gastric cancer. Furthermore, this gene has also been identified in the extracellular environment in a mouse model [87]. Thus, it may act as a potential liquid biopsy biomarker for the further classification of candidate tumor subtypes [88, 89]. RPL6 (ENSG00000089009) is also a component of the 60S ribosome. Based on the existing literature, this gene has been identified in colorectal cancer, lung cancer, breast cancer and gastric cancer, implying its potential as a typing marker for distinguishing colorectal cancer, breast cancer and lung cancer from the other three cancer subtypes and healthy control [90–92]. Similar to other ribosomal protein ligands, RPL6 has also been identified in the exosome as a functional ribosome associated component, implying that RPL6 may be a potential biomarker. RPL27 (ENSG00000131469) is a crucial component of the 60S subunit [93, 94]. In gastric cancer, head and neck squamous cell carcinoma, oral squamous cell carcinoma, hepatobiliary cancer and breast cancer, this gene has been confirmed to contribute to the initiation and progression of tumors, with specific expression in bodily fluids, including blood [95–99].

### Cell surface proteins (receptors and antigens)

Cell surface proteins can be generally divided into receptors and antigens based on their specific biological functions. Based on recent publications, such two group of proteins have been confirmed to be differentially expressed in tumor comparing to normal cells, which can be attributed to the different biological function and recognition mechanism of tumor cells. Differential expressed proteins not only act as potential biomarkers for tumor identification and classification but also reflect the diverse potential oncogenic mechanisms of different tumor subtypes. TRBC2, ENSG00000211772, encodes a specific region of the T-cell receptor beta-2 chain [100]. Based on recent publications, this gene has also been identified in multiple cancer subtypes, including the six cancer subtypes used in this study, implying that this gene is a crucial marker for the distinction of cancer patients and healthy controls. ENSG00000160654, CD3G, has also been extracted as a candidate biomarker. CD3G has been widely reported to participate in antigen recognition associated biological processes, coupling antigen recognition to specific intracellular signal transduction pathways [101, 102]. Encoding a specific protein that can be easily detected by liquid biopsy, CD3G has been confirmed to be differentially expressed, and may be a direct target of different functional microRNA targets different subtypes [103]. In breast cancer and colorectal cancer, this gene has been confirmed to be differentially expressed compared to normal controls, implying that CD3G can distinguish breast cancer and colorectal cancer samples from other tumor subtypes and normal controls [103]. Considering that CD3G and its regulatory miRNAs have already been detected in peripheral blood, it may be reasonable to use CD3G as a potential biomarker for liquid biopsy, validating our prediction [103]. Another cluster of differentiation (CD) protein, CD27 (ENSG00000139193), was also found in this study. As a member of the TNF-receptor superfamily, this gene has been identified in various cancer subtypes, including glioblastoma, breast cancer and colorectal cancer, but not lung cancer, pancreatic cancer or hepatobiliary cancer, by blood detection [104, 105]. Other studies have also confirmed that during tumorigenesis, CD27-containing exosomes can be identified in the peripheral blood of patients suffering from various cancer subtypes, validating the applicability and practicability of our predicted biomarkers [106]. Considering the differential expression of this gene in different cancer subtypes, it is quite reasonable to regard this gene as a potential biomarker for differential diagnosis. RPL9, or ribosomal protein L (ENSG00000163682), may also contribute to the classification of cancer subtypes and normal control based on the liquid biopsy results. As a ribosome-associated gene of the 60S subunit, this gene has been reported to contribute to various cancer subtypes, including lung cancer, hepatobiliary cancer, breast cancer and colorectal cancer, but not glioblastoma or pancreatic cancer [107]. Therefore, this gene can be a useful marker to distinguish glioblastoma, pancreatic cancer and healthy samples.

This study attempted to identify novel biomarkers (genes) that contribute to the classification of different cancer subtypes by analyzing gene expression data from RNA-seq results through computational methods. Eighteen identified genes were found to be differentially expressed in six cancer subtypes and healthy controls. All of these biomarkers were further classified into three groups, implying their crucial roles for tumorigenesis. In addition, we also propose an optimal classification method for the identification of six cancer subtypes and healthy controls, which can be a novel tool for diagnosing different cancer subtypes.

## MATERIALS AND METHODS

### Dataset and feature construction

We downloaded gene expression profiles of blood from 285 samples from the Gene Expression Omnibus (GEO) under the accession number GSE68086 [28]. These 285 samples were collected from patients who had one of the following cancer subtypes: breast cancer, colorectal cancer, glioblastoma, hepatobiliary cancer, lung cancer, or pancreatic cancer or from healthy controls. The detailed number of samples in each cancer subtype or healthy samples is listed in Table 3.

**Table 3:** Breakdown of 285 RNA-seq samples

Cancer subtype	Number of samples
Breast cancer	39
Colorectal cancer	42
Glioblastoma	40
Hepatobiliary cancer	14
Lung cancer	60
Pancreatic cancer	35
Healthy control	55

Because the expression levels of some genes from the 285 samples are quite weak, we discarded genes whose expression level in more than 90% of samples was zero, leaving 13,445 genes. Thus, each sample can be represented by 13,445 features, each of which indicates the expression level of a gene in the sample. Furthermore, the gene expression profiles were processed with quantile normalization and *log*2 transformed. The purpose of this study was to find optimal blood biomarkers for distinguishing various cancer patients.

### Feature selection method

As mentioned in Section “Dataset and feature construction”, all samples were represented by the expression levels of 13,445 genes. By extensively analyzing the samples, we can extract the genes that may be important biomarkers for different cancer subtypes. In this study, we employed a widely used and reliable feature selection method, the mRMR method [29], which has been applied to address different complicated biological and medical problems [108–121].

**Figure 6:**
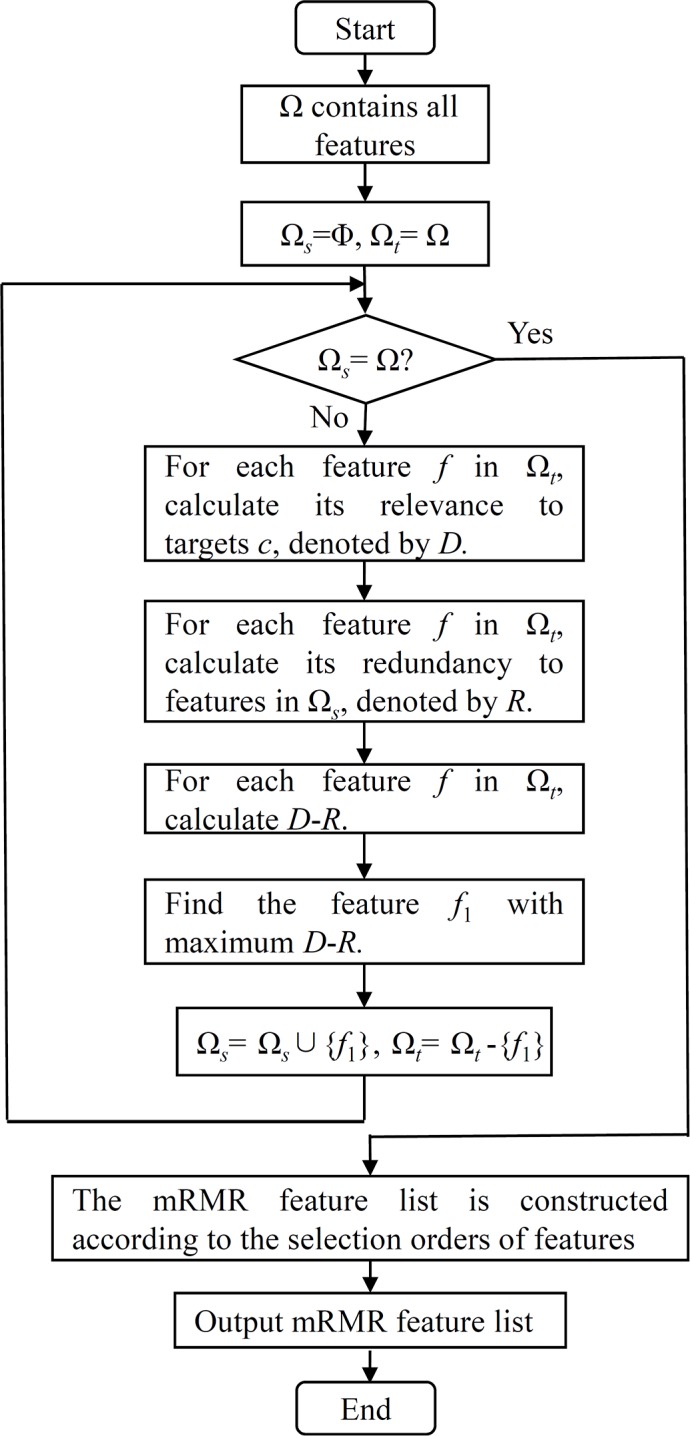
The flow chart of constructing the mRMR feature list in the mRMR method

The mRMR method is a mutual information (MI) based feature selection method. The correlations between features and targets are evaluated by the following MI equation:

$$I(x,y)=\iint p(x,y)\log\frac{p(x,y)}{p(x)p(y)}\text{dx dy}$$ (1)

where *p*(*x*, *y*) is the joint probabilistic density and *p*(*x*) and *p*(*y*) are the marginal probabilistic densities. A large MI value means that two variables have a strong correlation. The mRMR method evaluates each feature based on its relevance to targets and its redundancy to other features. Thus, two excellent criteria are used in the mRMR method: Max-Relevance and Min-Redundancy. The former indicates the importance of each feature based on its relevance to targets, while the latter assesses the importance of each feature using its redundancy to other features. By these two criteria, the mRMR method can produce two feature lists, the MaxRel feature list and mRMR feature list, in which all features are ranked rigorously. The MaxRel feature list ranks features according to their relevance to targets, *i.e.*, features are ranked in this list by the decreasing order of their MI values to targets. Production of the mRMR feature list is listed below and shown in Figure 6.

Given a dataset with *N* features, let Ω be a set consisting of all *N* features, Ω_s_ be a set containing selected features and Ω_t_ be the set consisting of the remaining features, *i.e.*, Ω_t_=Ω-Ω_t_s. Initially, Ω _s_ is set to be an empty set and all features are in Ω_t_. Then, a loop procedure is executed to move the features in Ω_t_ one by one to Ω_s_. For each feature *f* in Ω_t_, its relevance to targets *c* is calculated by *D = I(f, c)* and its redundancy to features in Ω_s_ is calculated by ($R=\frac{1}{\left|\Omega_{s}\right|}\sum_{f'\in \Omega_{s}}I(f,f')$). Because both the criteria of Max-Relevance and Min-Redundancy are considered when producing the mRMR feature list, we further calculate *D*-*R* for each feature in Ω_t_. The feature with the maximum *D*-*R* is selected and moved from Ω_t_ to Ω_s_. When all of the features are in Ω_s_, the loop procedure stops. Accordingly, the mRMR feature list can be ordered according to the selection orders of features, *i.e.*, the first selected feature occupies the first place, followed by the second selected feature, the third selected feature, and so forth. For formulation, the mRMR feature list was formulated as

$$F=[f_{1},f_{2},\ldots ,f_{N}]$$ (2)

The mRMR method only provides two feature lists for a given dataset. Clearly, the mRMR feature list can be used to extract the optimal subset of features for building an optimal classification model. Furthermore, a feature with a high rank in the mRMR feature list is more important for classification. However, we do not know how many top features in this list should be selected. To determine how many top features in this list should be selected, the incremental feature selection (IFS) method was employed in this study. This method evaluates the importance of several feature sets that contain some of the top features in *F* by testing their discriminating power in a classification algorithm.

In detail, for a feature set, say F_*i*_ = {f_*1*_,f_*2*_,…,f_*i*_}, containing the top *i* features in *F*, all samples are represented by the features in F_*i*_. Then, a classification algorithm is executed on these samples with its performance evaluated by one of the cross-validation methods [122–128]. After all of the possible feature sets have been tested, the feature set yielding the best performance can be found. This feature subset is deemed to be the optimal feature subset, and the features in this subset are called optimal features. At the same time, an optimal classification model can be built, which adopts the optimal features to represent samples. However, in many cases, it is quite time-consuming to test all possible feature subsets because there are too many possible feature subsets. In this case, only a part of possible feature subsets were tested. The obtained feature subset in this case is still called the optimal feature subset and the constructed classification model is still termed the optimal classification model for convenience.

### Classification algorithm

In the aforementioned IFS method, a classification algorithm is necessary. Here, we selected the classic machine learning algorithm, support vector machine (SVM) algorithm [30, 31]. This algorithm maps all samples into a higher dimensional space, in which these samples can be perfectly classified by a hyper-plane. Until now, several types of SVM algorithms have been proposed to tackle different types of classification problems. In this study, we chose to use the SVM algorithm trained by the sequential minimal optimization (SMO) algorithm [129] proposed by Platt. To train the SVM, a large quadratic program (QP) must be solved. The SMO algorithm breaks the large QP problem into several smallest QP problems and solves these QP sub-problems analytically. This procedure can avoid the storage of matrix and using the time-consuming numerical QP optimization as an inner loop. To quickly implement this type of SVM, we directly employed the classifier, SMO, in Weka [130] using its default parameters.

### Measurements

As mentioned in Section “Classification algorithm”, the SVM was adopted as the prediction engine. Ten-fold cross-validation [122] was employed to evaluate the performance of the SVM on different feature subsets. In this cross-validation method, the original dataset is randomly and equally divided into ten parts. Samples in each part are singled out as testing samples, and the remaining samples are used to train the classification model. Thus, each sample is tested exactly once. Compared with another cross-validation method, jackknife test [124, 131], ten-fold cross-validation needs much less time and yields similar results in most cases. Because our computational power was limited, we selected ten-fold cross-validation rather than jackknife test to evaluate the performance of the SVM in this study.

As listed in Table 1, all samples were classified into seven classes. To assess the predicted results yielded by a classification model, the prediction accuracy for the *j*-th class, denoted as *ACC_j_*, can be calculated as

$${\text{ACC}}_{j}=\frac{x_{j}}{X_{j}}$$ (3)

where *x_j_* represents the number of samples that are predicted correctly in the *j*-th class and *X_j_* represents the total number of samples in the *j*-th class. In addition, we can calculate the overall accuracy, denoted as *TACC*, to assess the performance of the classification model on the whole, which can be computed by

$$\text{TACC}=\frac{\sum_{j}x_{j}}{\sum_{j}X_{j}}$$ (4)

Clearly, the overall accuracy can be appropriately used as the major measurement to evaluate the performance of each classification model. The prediction accuracy of each class was also provided in this study as references.

Besides, to further analyze the predicted results yielded by each classification model, we calculated the sensitivity (SN) and specificity (SP) for the *j*-th class, which were defined as follows:

$$\left\{ \begin{array}{l} SN_{j}=\frac{TP_{j}}{TP_{j}+FN_{j}}\\ SP_{j}=\frac{TN_{j}}{TN_{j}+FP_{j}} \end{array}\right.$$ (5)

where *TP_j_* represented the number of correctly predicted samples in the *j*-th class, *FN_j_* represented the number of incorrectly predicted samples in the *j*-th class, *FP_j_* represented the number of samples in other classes that were predicted to be in the *j*-th class, and *TN_j_* represented the number of samples in other classes that were not predicted to be in the *j*-th class. It is easy to see that the sensitivity of one class is same as the prediction accuracy of that class.

## SUPPLEMENTARY TABLES
















