# Supplementary material for: Identifying and analyzing different cancer subtypes using RNA-seq data of blood platelets
Source: Oncotarget. 2017 Sep 15;8(50):87494–511. doi: 10.18632/oncotarget.20903 (PMC5675649; doi:10.18632/oncotarget.20903)
Supplement: Supplementary file 1 [file oncotarget-08-87494-s001.pdf]

Identifying and analyzing different cancer subtypes using RNA-seq data of blood platelets

SUPPLEMENTARY MATERIALS

Supplementary Table 1: The MaxRel feature list yielded by the mRMR method

See Supplementary File 1

Supplementary Table 2: The mRMR feature list yielded by the mRMR method

See Supplementary File 2

Supplementary Table 3: The prediction accuracy for each class and overall accuracy yielded by the first stage of the IFS method

See Supplementary File 3

Supplementary Table 4: The specificity for each class yielded by the first stage of the IFS method

See Supplementary File 4

Supplementary Table 5: The prediction accuracy for each class and overall accuracy yielded by the second stage of the IFS method

See Supplementary File 5

Supplementary Table 6: The specificity for each class yielded by the second stage of the IFS method

See Supplementary File 6

Supplementary Table 7: Cancer panel genes in seven commercial cancer detection panels

See Supplementary File 7
