# Supplementary material for: Identifying and analyzing different cancer subtypes using RNA-seq data of blood platelets
Source: Oncotarget. 2017 Sep 15;8(50):87494–511. doi: 10.18632/oncotarget.20903 (PMC5675649; doi:10.18632/oncotarget.20903)
Supplement: Supplementary file 5 [file oncotarget-08-87494-s005.docx]

**Supplementary Table 4:** **The specificity for each class yielded by the first stage of the IFS method**

| **Number of features** | **Breast cancer** | **Colorectal cancer** | **Glioblastoma** | **Healthy control** | **Hepatobiliary cancer** | **Lung cancer** | **Pancreas cancer** |
| --- | --- | --- | --- | --- | --- | --- | --- |
| 10 | 0.939 | 0.914 | 0.963 | 0.948 | 1.000 | 0.631 | 0.948 |
| 20 | 0.890 | 0.844 | 0.980 | 0.922 | 1.000 | 0.831 | 0.916 |
| 30 | 0.874 | 0.864 | 0.988 | 0.943 | 1.000 | 0.889 | 0.888 |
| 40 | 0.886 | 0.856 | 0.992 | 0.948 | 0.996 | 0.871 | 0.892 |
| 50 | 0.878 | 0.872 | 0.996 | 0.948 | 0.993 | 0.858 | 0.916 |
| 60 | 0.902 | 0.868 | 0.992 | 0.943 | 0.982 | 0.871 | 0.912 |
| 70 | 0.890 | 0.864 | 0.992 | 0.948 | 0.985 | 0.876 | 0.904 |
| 80 | 0.894 | 0.860 | 0.996 | 0.943 | 0.989 | 0.898 | 0.916 |
| 90 | 0.894 | 0.885 | 0.980 | 0.957 | 0.989 | 0.880 | 0.912 |
| 100 | 0.907 | 0.889 | 0.984 | 0.970 | 0.985 | 0.880 | 0.924 |
| 110 | 0.902 | 0.893 | 0.984 | 0.974 | 0.982 | 0.889 | 0.948 |
| 120 | 0.915 | 0.872 | 0.984 | 0.957 | 0.985 | 0.907 | 0.948 |
| 130 | 0.911 | 0.881 | 0.984 | 0.965 | 0.982 | 0.938 | 0.952 |
| 140 | 0.919 | 0.872 | 0.988 | 0.965 | 0.982 | 0.924 | 0.932 |
| 150 | 0.923 | 0.864 | 0.984 | 0.957 | 0.989 | 0.920 | 0.948 |
| 160 | 0.898 | 0.877 | 0.971 | 0.961 | 0.989 | 0.902 | 0.952 |
| 170 | 0.902 | 0.868 | 0.976 | 0.965 | 0.985 | 0.916 | 0.948 |
| 180 | 0.902 | 0.872 | 0.976 | 0.961 | 0.985 | 0.920 | 0.928 |
| 190 | 0.898 | 0.877 | 0.976 | 0.961 | 0.989 | 0.907 | 0.932 |
| 200 | 0.907 | 0.864 | 0.971 | 0.961 | 0.989 | 0.907 | 0.928 |
| 210 | 0.890 | 0.868 | 0.984 | 0.961 | 0.982 | 0.893 | 0.936 |
| 220 | 0.902 | 0.864 | 0.980 | 0.961 | 0.985 | 0.898 | 0.936 |
| 230 | 0.915 | 0.860 | 0.976 | 0.952 | 0.993 | 0.907 | 0.912 |
| 240 | 0.919 | 0.860 | 0.984 | 0.952 | 0.985 | 0.907 | 0.916 |
| 250 | 0.907 | 0.877 | 0.988 | 0.948 | 0.989 | 0.902 | 0.924 |
| 260 | 0.886 | 0.889 | 0.992 | 0.948 | 0.985 | 0.893 | 0.924 |
| 270 | 0.886 | 0.881 | 0.988 | 0.952 | 0.989 | 0.893 | 0.932 |
| 280 | 0.907 | 0.877 | 0.992 | 0.952 | 0.985 | 0.911 | 0.920 |
| 290 | 0.911 | 0.889 | 0.992 | 0.952 | 0.985 | 0.902 | 0.916 |
| 300 | 0.915 | 0.877 | 0.988 | 0.952 | 0.978 | 0.902 | 0.924 |
| 310 | 0.919 | 0.872 | 0.988 | 0.943 | 0.982 | 0.916 | 0.920 |
| 320 | 0.927 | 0.864 | 0.992 | 0.943 | 0.985 | 0.907 | 0.920 |
| 330 | 0.919 | 0.868 | 0.988 | 0.943 | 0.982 | 0.924 | 0.928 |
| 340 | 0.911 | 0.885 | 0.988 | 0.943 | 0.985 | 0.911 | 0.928 |
| 350 | 0.911 | 0.901 | 0.988 | 0.948 | 0.982 | 0.911 | 0.928 |
| 360 | 0.915 | 0.901 | 0.992 | 0.943 | 0.989 | 0.898 | 0.920 |
| 370 | 0.911 | 0.893 | 0.992 | 0.943 | 0.989 | 0.898 | 0.928 |
| 380 | 0.915 | 0.889 | 0.984 | 0.948 | 0.989 | 0.916 | 0.932 |
| 390 | 0.907 | 0.897 | 0.992 | 0.943 | 0.989 | 0.916 | 0.932 |
| 400 | 0.923 | 0.909 | 0.992 | 0.943 | 0.985 | 0.920 | 0.932 |
| 410 | 0.927 | 0.909 | 0.992 | 0.943 | 0.996 | 0.916 | 0.916 |
| 420 | 0.927 | 0.918 | 0.992 | 0.943 | 0.996 | 0.916 | 0.920 |
| 430 | 0.923 | 0.909 | 0.992 | 0.943 | 0.996 | 0.911 | 0.916 |
| 440 | 0.919 | 0.909 | 0.996 | 0.943 | 0.996 | 0.916 | 0.932 |
| 450 | 0.923 | 0.905 | 0.992 | 0.943 | 0.996 | 0.916 | 0.924 |
| 460 | 0.923 | 0.905 | 0.988 | 0.943 | 0.996 | 0.929 | 0.924 |
| 470 | 0.919 | 0.922 | 0.988 | 0.943 | 0.996 | 0.924 | 0.916 |
| 480 | 0.931 | 0.918 | 0.992 | 0.943 | 0.993 | 0.929 | 0.920 |
| 490 | 0.943 | 0.905 | 0.992 | 0.943 | 0.993 | 0.947 | 0.912 |
| 500 | 0.923 | 0.901 | 0.988 | 0.948 | 0.996 | 0.938 | 0.908 |
| 510 | 0.919 | 0.905 | 0.988 | 0.948 | 0.996 | 0.938 | 0.912 |
| 520 | 0.931 | 0.905 | 0.992 | 0.948 | 0.996 | 0.938 | 0.908 |
| 530 | 0.923 | 0.909 | 0.992 | 0.943 | 0.996 | 0.938 | 0.904 |
| 540 | 0.931 | 0.909 | 1.000 | 0.943 | 0.996 | 0.929 | 0.904 |
| 550 | 0.947 | 0.914 | 0.992 | 0.948 | 0.996 | 0.933 | 0.912 |
| 560 | 0.927 | 0.914 | 0.992 | 0.948 | 0.996 | 0.933 | 0.916 |
| 570 | 0.927 | 0.914 | 0.992 | 0.952 | 0.996 | 0.938 | 0.920 |
| 580 | 0.931 | 0.909 | 0.992 | 0.948 | 0.996 | 0.938 | 0.916 |
| 590 | 0.923 | 0.914 | 0.992 | 0.948 | 0.996 | 0.942 | 0.928 |
| 600 | 0.923 | 0.918 | 0.992 | 0.952 | 0.993 | 0.933 | 0.932 |
| 610 | 0.923 | 0.905 | 0.996 | 0.948 | 0.996 | 0.942 | 0.924 |
| 620 | 0.923 | 0.918 | 0.996 | 0.943 | 0.989 | 0.942 | 0.936 |
| 630 | 0.927 | 0.909 | 0.996 | 0.948 | 0.993 | 0.942 | 0.924 |
| 640 | 0.923 | 0.909 | 0.996 | 0.948 | 0.993 | 0.942 | 0.928 |
| 650 | 0.919 | 0.918 | 0.992 | 0.952 | 0.993 | 0.942 | 0.928 |
| 660 | 0.923 | 0.905 | 0.996 | 0.952 | 0.993 | 0.942 | 0.932 |
| 670 | 0.923 | 0.901 | 0.996 | 0.952 | 0.993 | 0.938 | 0.924 |
| 680 | 0.931 | 0.901 | 0.996 | 0.952 | 0.996 | 0.933 | 0.928 |
| 690 | 0.931 | 0.914 | 0.996 | 0.952 | 0.996 | 0.938 | 0.912 |
| 700 | 0.931 | 0.926 | 0.996 | 0.948 | 0.996 | 0.933 | 0.920 |
| 710 | 0.927 | 0.914 | 0.992 | 0.948 | 0.996 | 0.933 | 0.928 |
| 720 | 0.923 | 0.909 | 0.988 | 0.948 | 0.996 | 0.942 | 0.920 |
| 730 | 0.919 | 0.918 | 0.992 | 0.948 | 0.996 | 0.942 | 0.924 |
| 740 | 0.919 | 0.918 | 0.996 | 0.948 | 0.996 | 0.947 | 0.916 |
| 750 | 0.919 | 0.914 | 0.996 | 0.948 | 0.996 | 0.942 | 0.916 |
| 760 | 0.927 | 0.909 | 0.992 | 0.948 | 0.996 | 0.942 | 0.916 |
| 770 | 0.923 | 0.901 | 0.996 | 0.948 | 1.000 | 0.938 | 0.920 |
| 780 | 0.915 | 0.909 | 0.996 | 0.948 | 0.996 | 0.938 | 0.916 |
| 790 | 0.923 | 0.893 | 0.992 | 0.948 | 0.996 | 0.938 | 0.916 |
| 800 | 0.927 | 0.889 | 0.992 | 0.948 | 0.996 | 0.938 | 0.912 |
| 810 | 0.915 | 0.893 | 0.996 | 0.943 | 0.996 | 0.933 | 0.916 |
| 820 | 0.915 | 0.893 | 0.996 | 0.943 | 0.996 | 0.929 | 0.916 |
| 830 | 0.911 | 0.905 | 0.996 | 0.943 | 0.996 | 0.933 | 0.904 |
| 840 | 0.911 | 0.909 | 0.996 | 0.943 | 0.993 | 0.938 | 0.908 |
| 850 | 0.915 | 0.897 | 1.000 | 0.943 | 0.993 | 0.938 | 0.904 |
| 860 | 0.915 | 0.893 | 0.996 | 0.943 | 0.996 | 0.942 | 0.900 |
| 870 | 0.907 | 0.905 | 0.996 | 0.943 | 0.996 | 0.947 | 0.904 |
| 880 | 0.911 | 0.901 | 0.996 | 0.943 | 0.996 | 0.947 | 0.908 |
| 890 | 0.911 | 0.901 | 0.996 | 0.943 | 0.996 | 0.947 | 0.908 |
| 900 | 0.919 | 0.905 | 0.996 | 0.943 | 0.993 | 0.938 | 0.908 |
| 910 | 0.927 | 0.901 | 0.996 | 0.943 | 0.993 | 0.942 | 0.908 |
| 920 | 0.919 | 0.901 | 0.996 | 0.943 | 0.996 | 0.942 | 0.908 |
| 930 | 0.923 | 0.914 | 0.996 | 0.948 | 0.996 | 0.938 | 0.912 |
| 940 | 0.931 | 0.909 | 0.996 | 0.948 | 0.996 | 0.938 | 0.912 |
| 950 | 0.927 | 0.909 | 0.996 | 0.948 | 0.996 | 0.938 | 0.916 |
| 960 | 0.923 | 0.901 | 0.996 | 0.948 | 0.996 | 0.942 | 0.912 |
| 970 | 0.927 | 0.901 | 0.996 | 0.948 | 0.996 | 0.942 | 0.912 |
| 980 | 0.923 | 0.909 | 0.996 | 0.948 | 0.996 | 0.947 | 0.912 |
| 990 | 0.923 | 0.901 | 0.996 | 0.948 | 0.996 | 0.942 | 0.912 |
| 1000 | 0.923 | 0.909 | 0.996 | 0.948 | 0.996 | 0.942 | 0.912 |
| 1010 | 0.923 | 0.905 | 0.996 | 0.948 | 0.996 | 0.942 | 0.920 |
| 1020 | 0.927 | 0.909 | 0.996 | 0.948 | 0.996 | 0.947 | 0.912 |
| 1030 | 0.927 | 0.909 | 0.996 | 0.948 | 0.996 | 0.947 | 0.908 |
| 1040 | 0.919 | 0.914 | 0.996 | 0.948 | 0.996 | 0.947 | 0.912 |
| 1050 | 0.927 | 0.909 | 0.996 | 0.948 | 0.996 | 0.947 | 0.916 |
| 1060 | 0.923 | 0.918 | 0.996 | 0.948 | 0.996 | 0.951 | 0.916 |
| 1070 | 0.931 | 0.922 | 0.996 | 0.943 | 0.996 | 0.951 | 0.908 |
| 1080 | 0.927 | 0.918 | 0.996 | 0.943 | 0.996 | 0.947 | 0.908 |
| 1090 | 0.927 | 0.918 | 0.996 | 0.943 | 0.996 | 0.947 | 0.912 |
| 1100 | 0.927 | 0.914 | 0.996 | 0.943 | 0.996 | 0.947 | 0.912 |
| 1110 | 0.927 | 0.918 | 0.996 | 0.943 | 0.996 | 0.951 | 0.912 |
| 1120 | 0.931 | 0.918 | 0.996 | 0.943 | 0.996 | 0.942 | 0.912 |
| 1130 | 0.931 | 0.918 | 0.996 | 0.943 | 0.996 | 0.942 | 0.912 |
| 1140 | 0.931 | 0.918 | 0.992 | 0.943 | 0.996 | 0.938 | 0.912 |
| 1150 | 0.931 | 0.918 | 0.992 | 0.943 | 0.996 | 0.938 | 0.920 |
| 1160 | 0.927 | 0.926 | 0.992 | 0.943 | 0.996 | 0.938 | 0.924 |
| 1170 | 0.927 | 0.922 | 0.992 | 0.943 | 0.996 | 0.942 | 0.924 |
| 1180 | 0.927 | 0.922 | 0.992 | 0.943 | 0.996 | 0.938 | 0.928 |
| 1190 | 0.927 | 0.922 | 0.988 | 0.943 | 0.996 | 0.942 | 0.920 |
| 1200 | 0.923 | 0.914 | 0.988 | 0.948 | 0.996 | 0.938 | 0.932 |
| 1210 | 0.919 | 0.914 | 0.988 | 0.948 | 0.996 | 0.942 | 0.936 |
| 1220 | 0.923 | 0.930 | 0.988 | 0.948 | 0.996 | 0.938 | 0.928 |
| 1230 | 0.923 | 0.926 | 0.988 | 0.948 | 0.996 | 0.938 | 0.932 |
| 1240 | 0.919 | 0.918 | 0.988 | 0.952 | 0.996 | 0.938 | 0.944 |
| 1250 | 0.931 | 0.918 | 0.988 | 0.952 | 0.996 | 0.933 | 0.940 |
| 1260 | 0.931 | 0.926 | 0.988 | 0.952 | 0.996 | 0.929 | 0.940 |
| 1270 | 0.927 | 0.922 | 0.988 | 0.952 | 0.996 | 0.929 | 0.936 |
| 1280 | 0.927 | 0.926 | 0.988 | 0.952 | 0.996 | 0.929 | 0.932 |
| 1290 | 0.915 | 0.926 | 0.988 | 0.952 | 0.996 | 0.929 | 0.936 |
| 1300 | 0.919 | 0.930 | 0.988 | 0.952 | 0.996 | 0.929 | 0.932 |
| 1310 | 0.923 | 0.926 | 0.988 | 0.952 | 0.996 | 0.920 | 0.936 |
| 1320 | 0.919 | 0.930 | 0.988 | 0.952 | 0.996 | 0.920 | 0.932 |
| 1330 | 0.919 | 0.930 | 0.988 | 0.952 | 0.996 | 0.924 | 0.936 |
| 1340 | 0.923 | 0.930 | 0.988 | 0.948 | 0.996 | 0.924 | 0.936 |
| 1350 | 0.919 | 0.926 | 0.988 | 0.948 | 0.996 | 0.924 | 0.944 |
| 1360 | 0.923 | 0.926 | 0.988 | 0.948 | 0.996 | 0.924 | 0.940 |
| 1370 | 0.923 | 0.922 | 0.988 | 0.948 | 0.996 | 0.924 | 0.944 |
| 1380 | 0.923 | 0.922 | 0.988 | 0.952 | 0.996 | 0.920 | 0.944 |
| 1390 | 0.923 | 0.922 | 0.988 | 0.952 | 0.996 | 0.924 | 0.940 |
| 1400 | 0.927 | 0.918 | 0.988 | 0.948 | 0.996 | 0.924 | 0.948 |
| 1410 | 0.927 | 0.922 | 0.988 | 0.948 | 0.996 | 0.924 | 0.948 |
| 1420 | 0.931 | 0.926 | 0.988 | 0.948 | 0.996 | 0.924 | 0.944 |
| 1430 | 0.931 | 0.926 | 0.988 | 0.948 | 0.996 | 0.924 | 0.952 |
| 1440 | 0.931 | 0.922 | 0.988 | 0.948 | 0.996 | 0.924 | 0.952 |
| 1450 | 0.931 | 0.918 | 0.988 | 0.948 | 0.996 | 0.924 | 0.944 |
| 1460 | 0.931 | 0.922 | 0.988 | 0.948 | 0.996 | 0.920 | 0.948 |
| 1470 | 0.927 | 0.922 | 0.988 | 0.952 | 0.996 | 0.920 | 0.948 |
| 1480 | 0.927 | 0.926 | 0.988 | 0.952 | 0.996 | 0.920 | 0.940 |
| 1490 | 0.927 | 0.918 | 0.988 | 0.952 | 0.996 | 0.924 | 0.944 |
| 1500 | 0.927 | 0.922 | 0.988 | 0.952 | 0.996 | 0.920 | 0.944 |
| 1510 | 0.927 | 0.922 | 0.988 | 0.952 | 0.996 | 0.924 | 0.940 |
| 1520 | 0.927 | 0.918 | 0.988 | 0.952 | 0.996 | 0.924 | 0.948 |
| 1530 | 0.927 | 0.926 | 0.988 | 0.952 | 0.996 | 0.924 | 0.948 |
| 1540 | 0.927 | 0.926 | 0.988 | 0.952 | 0.996 | 0.924 | 0.948 |
| 1550 | 0.927 | 0.926 | 0.988 | 0.952 | 0.996 | 0.924 | 0.948 |
| 1560 | 0.927 | 0.926 | 0.988 | 0.952 | 0.996 | 0.929 | 0.944 |
| 1570 | 0.927 | 0.926 | 0.988 | 0.952 | 0.996 | 0.924 | 0.952 |
| 1580 | 0.927 | 0.922 | 0.988 | 0.952 | 0.996 | 0.920 | 0.956 |
| 1590 | 0.923 | 0.930 | 0.988 | 0.952 | 0.996 | 0.916 | 0.960 |
| 1600 | 0.927 | 0.926 | 0.988 | 0.952 | 0.996 | 0.920 | 0.956 |
| 1610 | 0.927 | 0.918 | 0.988 | 0.952 | 0.996 | 0.916 | 0.956 |
| 1620 | 0.927 | 0.926 | 0.988 | 0.952 | 0.996 | 0.920 | 0.956 |
| 1630 | 0.927 | 0.926 | 0.988 | 0.952 | 0.996 | 0.924 | 0.956 |
| 1640 | 0.927 | 0.926 | 0.988 | 0.952 | 0.996 | 0.924 | 0.956 |
| 1650 | 0.927 | 0.934 | 0.988 | 0.957 | 0.996 | 0.924 | 0.948 |
| 1660 | 0.927 | 0.930 | 0.988 | 0.952 | 0.996 | 0.924 | 0.956 |
| 1670 | 0.927 | 0.934 | 0.988 | 0.952 | 0.996 | 0.924 | 0.952 |
| 1680 | 0.927 | 0.934 | 0.988 | 0.952 | 0.996 | 0.924 | 0.952 |
| 1690 | 0.927 | 0.938 | 0.988 | 0.952 | 0.996 | 0.920 | 0.952 |
| 1700 | 0.927 | 0.938 | 0.988 | 0.952 | 0.996 | 0.916 | 0.952 |
| 1710 | 0.927 | 0.930 | 0.988 | 0.948 | 0.996 | 0.916 | 0.952 |
| 1720 | 0.927 | 0.930 | 0.988 | 0.948 | 0.996 | 0.911 | 0.952 |
| 1730 | 0.927 | 0.934 | 0.988 | 0.948 | 0.996 | 0.920 | 0.952 |
| 1740 | 0.927 | 0.930 | 0.988 | 0.948 | 0.996 | 0.920 | 0.952 |
| 1750 | 0.923 | 0.930 | 0.984 | 0.948 | 0.996 | 0.916 | 0.948 |
| 1760 | 0.927 | 0.938 | 0.988 | 0.948 | 0.996 | 0.916 | 0.948 |
| 1770 | 0.923 | 0.942 | 0.988 | 0.948 | 0.996 | 0.920 | 0.956 |
| 1780 | 0.927 | 0.930 | 0.988 | 0.948 | 0.996 | 0.920 | 0.952 |
| 1790 | 0.931 | 0.922 | 0.988 | 0.948 | 0.996 | 0.916 | 0.952 |
| 1800 | 0.923 | 0.922 | 0.988 | 0.948 | 0.996 | 0.916 | 0.960 |
| 1810 | 0.931 | 0.922 | 0.984 | 0.948 | 0.996 | 0.916 | 0.956 |
| 1820 | 0.927 | 0.926 | 0.984 | 0.948 | 0.996 | 0.916 | 0.956 |
| 1830 | 0.927 | 0.918 | 0.988 | 0.948 | 0.996 | 0.920 | 0.956 |
| 1840 | 0.931 | 0.914 | 0.988 | 0.948 | 0.996 | 0.924 | 0.948 |
| 1850 | 0.931 | 0.914 | 0.988 | 0.948 | 0.996 | 0.929 | 0.948 |
| 1860 | 0.931 | 0.914 | 0.984 | 0.948 | 0.996 | 0.929 | 0.952 |
| 1870 | 0.927 | 0.909 | 0.988 | 0.952 | 0.996 | 0.924 | 0.964 |
| 1880 | 0.931 | 0.909 | 0.988 | 0.952 | 0.996 | 0.924 | 0.964 |
| 1890 | 0.923 | 0.922 | 0.988 | 0.952 | 0.996 | 0.924 | 0.956 |
| 1900 | 0.923 | 0.922 | 0.988 | 0.952 | 0.996 | 0.920 | 0.960 |
| 1910 | 0.923 | 0.922 | 0.988 | 0.952 | 0.996 | 0.924 | 0.964 |
| 1920 | 0.927 | 0.926 | 0.988 | 0.952 | 0.996 | 0.920 | 0.956 |
| 1930 | 0.927 | 0.926 | 0.988 | 0.952 | 0.996 | 0.924 | 0.948 |
| 1940 | 0.931 | 0.926 | 0.988 | 0.948 | 0.996 | 0.920 | 0.956 |
| 1950 | 0.923 | 0.922 | 0.992 | 0.948 | 0.996 | 0.920 | 0.960 |
| 1960 | 0.927 | 0.926 | 0.992 | 0.948 | 0.996 | 0.920 | 0.964 |
| 1970 | 0.927 | 0.922 | 0.988 | 0.948 | 0.996 | 0.920 | 0.964 |
| 1980 | 0.927 | 0.914 | 0.988 | 0.948 | 0.996 | 0.920 | 0.964 |
| 1990 | 0.927 | 0.918 | 0.988 | 0.948 | 0.996 | 0.920 | 0.952 |
| 2000 | 0.931 | 0.922 | 0.988 | 0.948 | 0.996 | 0.920 | 0.956 |
| 2010 | 0.927 | 0.926 | 0.992 | 0.952 | 0.996 | 0.920 | 0.956 |
| 2020 | 0.935 | 0.918 | 0.992 | 0.952 | 0.996 | 0.920 | 0.960 |
| 2030 | 0.935 | 0.934 | 0.992 | 0.952 | 0.996 | 0.920 | 0.968 |
| 2040 | 0.935 | 0.934 | 0.992 | 0.952 | 0.996 | 0.924 | 0.960 |
| 2050 | 0.935 | 0.934 | 0.992 | 0.948 | 0.996 | 0.924 | 0.960 |
| 2060 | 0.931 | 0.930 | 0.992 | 0.952 | 0.996 | 0.924 | 0.956 |
| 2070 | 0.931 | 0.926 | 0.992 | 0.952 | 0.996 | 0.929 | 0.956 |
| 2080 | 0.931 | 0.934 | 0.992 | 0.952 | 0.996 | 0.929 | 0.952 |
| 2090 | 0.931 | 0.930 | 0.992 | 0.952 | 0.996 | 0.929 | 0.952 |
| 2100 | 0.939 | 0.922 | 0.992 | 0.952 | 0.996 | 0.929 | 0.952 |
| 2110 | 0.939 | 0.922 | 0.992 | 0.952 | 0.996 | 0.929 | 0.956 |
| 2120 | 0.939 | 0.918 | 0.992 | 0.952 | 0.996 | 0.929 | 0.956 |
| 2130 | 0.939 | 0.926 | 0.992 | 0.952 | 0.996 | 0.929 | 0.960 |
| 2140 | 0.939 | 0.914 | 0.992 | 0.952 | 0.996 | 0.924 | 0.960 |
| 2150 | 0.935 | 0.909 | 0.992 | 0.952 | 0.996 | 0.929 | 0.968 |
| 2160 | 0.939 | 0.909 | 0.992 | 0.952 | 0.996 | 0.929 | 0.964 |
| 2170 | 0.931 | 0.918 | 0.992 | 0.952 | 0.996 | 0.929 | 0.972 |
| 2180 | 0.935 | 0.918 | 0.992 | 0.952 | 0.996 | 0.924 | 0.964 |
| 2190 | 0.935 | 0.918 | 0.992 | 0.952 | 0.996 | 0.924 | 0.964 |
| 2200 | 0.939 | 0.909 | 0.992 | 0.952 | 0.996 | 0.920 | 0.960 |
| 2210 | 0.939 | 0.909 | 0.992 | 0.952 | 0.996 | 0.924 | 0.964 |
| 2220 | 0.939 | 0.914 | 0.992 | 0.952 | 0.996 | 0.924 | 0.960 |
| 2230 | 0.939 | 0.914 | 0.992 | 0.952 | 0.996 | 0.924 | 0.960 |
| 2240 | 0.935 | 0.918 | 0.992 | 0.952 | 0.996 | 0.924 | 0.960 |
| 2250 | 0.935 | 0.918 | 0.992 | 0.952 | 0.996 | 0.924 | 0.960 |
| 2260 | 0.935 | 0.922 | 0.992 | 0.952 | 0.996 | 0.924 | 0.956 |
| 2270 | 0.939 | 0.918 | 0.992 | 0.952 | 0.996 | 0.924 | 0.956 |
| 2280 | 0.939 | 0.914 | 0.992 | 0.952 | 0.996 | 0.924 | 0.956 |
| 2290 | 0.939 | 0.914 | 0.992 | 0.948 | 0.996 | 0.929 | 0.956 |
| 2300 | 0.939 | 0.909 | 0.992 | 0.948 | 0.996 | 0.929 | 0.952 |
| 2310 | 0.939 | 0.909 | 0.992 | 0.948 | 0.996 | 0.929 | 0.952 |
| 2320 | 0.939 | 0.922 | 0.992 | 0.948 | 0.996 | 0.924 | 0.948 |
| 2330 | 0.939 | 0.922 | 0.992 | 0.948 | 0.996 | 0.924 | 0.948 |
| 2340 | 0.939 | 0.922 | 0.992 | 0.948 | 0.996 | 0.924 | 0.948 |
| 2350 | 0.943 | 0.905 | 0.992 | 0.948 | 0.996 | 0.929 | 0.952 |
| 2360 | 0.939 | 0.918 | 0.992 | 0.948 | 0.996 | 0.933 | 0.948 |
| 2370 | 0.935 | 0.914 | 0.992 | 0.948 | 0.996 | 0.933 | 0.948 |
| 2380 | 0.939 | 0.909 | 0.992 | 0.952 | 0.996 | 0.933 | 0.948 |
| 2390 | 0.935 | 0.918 | 0.992 | 0.952 | 0.996 | 0.933 | 0.940 |
| 2400 | 0.931 | 0.918 | 0.992 | 0.952 | 0.996 | 0.929 | 0.944 |
| 2410 | 0.935 | 0.914 | 0.992 | 0.948 | 0.996 | 0.933 | 0.940 |
| 2420 | 0.935 | 0.909 | 0.992 | 0.948 | 0.996 | 0.933 | 0.948 |
| 2430 | 0.935 | 0.914 | 0.992 | 0.948 | 0.996 | 0.933 | 0.940 |
| 2440 | 0.943 | 0.905 | 0.992 | 0.948 | 0.996 | 0.929 | 0.944 |
| 2450 | 0.939 | 0.918 | 0.992 | 0.948 | 0.996 | 0.924 | 0.940 |
| 2460 | 0.939 | 0.918 | 0.992 | 0.948 | 0.996 | 0.924 | 0.940 |
| 2470 | 0.943 | 0.914 | 0.992 | 0.948 | 0.996 | 0.929 | 0.940 |
| 2480 | 0.935 | 0.922 | 0.992 | 0.948 | 0.996 | 0.929 | 0.940 |
| 2490 | 0.947 | 0.909 | 0.992 | 0.952 | 0.996 | 0.924 | 0.936 |
| 2500 | 0.943 | 0.905 | 0.992 | 0.952 | 0.996 | 0.929 | 0.932 |
| 2510 | 0.943 | 0.905 | 0.992 | 0.952 | 0.996 | 0.929 | 0.936 |
| 2520 | 0.939 | 0.914 | 0.992 | 0.948 | 0.996 | 0.929 | 0.936 |
| 2530 | 0.939 | 0.914 | 0.992 | 0.948 | 0.996 | 0.924 | 0.936 |
| 2540 | 0.947 | 0.905 | 0.992 | 0.952 | 0.996 | 0.920 | 0.940 |
| 2550 | 0.947 | 0.905 | 0.992 | 0.952 | 0.996 | 0.924 | 0.940 |
| 2560 | 0.947 | 0.905 | 0.992 | 0.952 | 0.996 | 0.920 | 0.940 |
| 2570 | 0.947 | 0.901 | 0.992 | 0.952 | 0.996 | 0.920 | 0.948 |
| 2580 | 0.947 | 0.897 | 0.992 | 0.948 | 0.996 | 0.924 | 0.944 |
| 2590 | 0.947 | 0.909 | 0.992 | 0.948 | 0.996 | 0.924 | 0.944 |
| 2600 | 0.947 | 0.901 | 0.992 | 0.948 | 0.996 | 0.924 | 0.956 |
| 2610 | 0.947 | 0.905 | 0.992 | 0.948 | 0.996 | 0.924 | 0.952 |
| 2620 | 0.947 | 0.909 | 0.992 | 0.948 | 0.996 | 0.924 | 0.956 |
| 2630 | 0.947 | 0.914 | 0.992 | 0.948 | 0.996 | 0.924 | 0.948 |
| 2640 | 0.947 | 0.914 | 0.992 | 0.948 | 0.996 | 0.924 | 0.948 |
| 2650 | 0.943 | 0.914 | 0.992 | 0.948 | 0.996 | 0.924 | 0.952 |
| 2660 | 0.943 | 0.905 | 0.992 | 0.948 | 0.996 | 0.924 | 0.956 |
| 2670 | 0.939 | 0.909 | 0.992 | 0.948 | 0.996 | 0.929 | 0.956 |
| 2680 | 0.939 | 0.909 | 0.992 | 0.948 | 0.996 | 0.929 | 0.956 |
| 2690 | 0.943 | 0.909 | 0.992 | 0.948 | 0.996 | 0.924 | 0.952 |
| 2700 | 0.943 | 0.909 | 0.992 | 0.948 | 0.996 | 0.924 | 0.952 |
| 2710 | 0.943 | 0.905 | 0.992 | 0.948 | 0.996 | 0.924 | 0.948 |
| 2720 | 0.939 | 0.914 | 0.992 | 0.948 | 0.996 | 0.924 | 0.948 |
| 2730 | 0.939 | 0.914 | 0.992 | 0.948 | 0.996 | 0.924 | 0.948 |
| 2740 | 0.939 | 0.914 | 0.992 | 0.948 | 0.996 | 0.924 | 0.944 |
| 2750 | 0.939 | 0.909 | 0.992 | 0.952 | 0.996 | 0.929 | 0.948 |
| 2760 | 0.939 | 0.909 | 0.992 | 0.952 | 0.996 | 0.924 | 0.948 |
| 2770 | 0.943 | 0.909 | 0.992 | 0.952 | 0.996 | 0.929 | 0.944 |
| 2780 | 0.939 | 0.914 | 0.992 | 0.952 | 0.996 | 0.929 | 0.940 |
| 2790 | 0.939 | 0.918 | 0.992 | 0.952 | 0.996 | 0.920 | 0.952 |
| 2800 | 0.943 | 0.918 | 0.992 | 0.952 | 0.996 | 0.929 | 0.940 |
| 2810 | 0.947 | 0.914 | 0.992 | 0.952 | 0.996 | 0.924 | 0.944 |
| 2820 | 0.947 | 0.914 | 0.992 | 0.952 | 0.996 | 0.924 | 0.940 |
| 2830 | 0.947 | 0.914 | 0.992 | 0.952 | 0.996 | 0.924 | 0.944 |
| 2840 | 0.943 | 0.914 | 0.992 | 0.952 | 0.996 | 0.924 | 0.948 |
| 2850 | 0.947 | 0.909 | 0.992 | 0.952 | 0.996 | 0.920 | 0.952 |
| 2860 | 0.951 | 0.905 | 0.992 | 0.952 | 1.000 | 0.924 | 0.948 |
| 2870 | 0.947 | 0.905 | 0.992 | 0.952 | 1.000 | 0.924 | 0.948 |
| 2880 | 0.943 | 0.905 | 0.992 | 0.952 | 1.000 | 0.920 | 0.948 |
| 2890 | 0.951 | 0.905 | 0.992 | 0.952 | 1.000 | 0.920 | 0.948 |
| 2900 | 0.951 | 0.909 | 0.992 | 0.952 | 1.000 | 0.920 | 0.952 |
| 2910 | 0.947 | 0.914 | 0.992 | 0.952 | 1.000 | 0.916 | 0.952 |
| 2920 | 0.951 | 0.905 | 0.992 | 0.952 | 1.000 | 0.920 | 0.948 |
| 2930 | 0.951 | 0.901 | 0.992 | 0.952 | 1.000 | 0.916 | 0.952 |
| 2940 | 0.951 | 0.905 | 0.992 | 0.952 | 1.000 | 0.916 | 0.948 |
| 2950 | 0.951 | 0.905 | 0.992 | 0.952 | 1.000 | 0.916 | 0.952 |
| 2960 | 0.951 | 0.914 | 0.992 | 0.952 | 1.000 | 0.920 | 0.948 |
| 2970 | 0.951 | 0.909 | 0.992 | 0.948 | 1.000 | 0.920 | 0.952 |
| 2980 | 0.951 | 0.909 | 0.992 | 0.948 | 1.000 | 0.920 | 0.952 |
| 2990 | 0.951 | 0.909 | 0.992 | 0.952 | 1.000 | 0.920 | 0.948 |
| 3000 | 0.955 | 0.909 | 0.992 | 0.948 | 1.000 | 0.920 | 0.952 |
| 3010 | 0.951 | 0.909 | 0.992 | 0.948 | 1.000 | 0.920 | 0.952 |
| 3020 | 0.951 | 0.905 | 0.992 | 0.948 | 1.000 | 0.920 | 0.952 |
| 3030 | 0.951 | 0.914 | 0.992 | 0.948 | 0.996 | 0.920 | 0.952 |
| 3040 | 0.951 | 0.909 | 0.992 | 0.948 | 0.996 | 0.920 | 0.952 |
| 3050 | 0.951 | 0.914 | 0.992 | 0.948 | 0.996 | 0.920 | 0.952 |
| 3060 | 0.951 | 0.914 | 0.992 | 0.948 | 0.996 | 0.920 | 0.952 |
| 3070 | 0.955 | 0.909 | 0.992 | 0.948 | 0.996 | 0.920 | 0.948 |
| 3080 | 0.955 | 0.909 | 0.992 | 0.948 | 0.996 | 0.920 | 0.948 |
| 3090 | 0.955 | 0.909 | 0.992 | 0.948 | 0.996 | 0.920 | 0.948 |
| 3100 | 0.951 | 0.909 | 0.992 | 0.948 | 0.996 | 0.920 | 0.948 |
| 3110 | 0.951 | 0.909 | 0.992 | 0.952 | 0.996 | 0.920 | 0.948 |
| 3120 | 0.951 | 0.905 | 0.992 | 0.948 | 0.996 | 0.920 | 0.948 |
| 3130 | 0.951 | 0.914 | 0.992 | 0.952 | 0.996 | 0.920 | 0.952 |
| 3140 | 0.951 | 0.909 | 0.992 | 0.948 | 0.996 | 0.920 | 0.944 |
| 3150 | 0.951 | 0.909 | 0.992 | 0.948 | 0.996 | 0.920 | 0.948 |
| 3160 | 0.951 | 0.909 | 0.992 | 0.948 | 0.996 | 0.920 | 0.948 |
| 3170 | 0.955 | 0.909 | 0.992 | 0.948 | 0.996 | 0.920 | 0.948 |
| 3180 | 0.951 | 0.914 | 0.992 | 0.952 | 0.996 | 0.920 | 0.952 |
| 3190 | 0.951 | 0.914 | 0.992 | 0.952 | 1.000 | 0.920 | 0.948 |
| 3200 | 0.951 | 0.918 | 0.992 | 0.952 | 1.000 | 0.920 | 0.948 |
| 3210 | 0.951 | 0.918 | 0.992 | 0.952 | 0.996 | 0.920 | 0.952 |
| 3220 | 0.955 | 0.909 | 0.992 | 0.948 | 1.000 | 0.920 | 0.944 |
| 3230 | 0.951 | 0.914 | 0.992 | 0.948 | 1.000 | 0.920 | 0.952 |
| 3240 | 0.951 | 0.914 | 0.992 | 0.952 | 1.000 | 0.920 | 0.948 |
| 3250 | 0.951 | 0.914 | 0.992 | 0.948 | 1.000 | 0.920 | 0.948 |
| 3260 | 0.951 | 0.909 | 0.992 | 0.952 | 1.000 | 0.920 | 0.948 |
| 3270 | 0.947 | 0.914 | 0.992 | 0.948 | 1.000 | 0.916 | 0.952 |
| 3280 | 0.943 | 0.909 | 0.992 | 0.952 | 1.000 | 0.916 | 0.952 |
| 3290 | 0.943 | 0.909 | 0.992 | 0.952 | 1.000 | 0.916 | 0.952 |
| 3300 | 0.943 | 0.909 | 0.992 | 0.952 | 1.000 | 0.916 | 0.952 |
| 3310 | 0.943 | 0.918 | 0.992 | 0.952 | 1.000 | 0.916 | 0.952 |
| 3320 | 0.947 | 0.914 | 0.992 | 0.952 | 1.000 | 0.916 | 0.948 |
| 3330 | 0.947 | 0.914 | 0.992 | 0.952 | 1.000 | 0.916 | 0.948 |
| 3340 | 0.947 | 0.918 | 0.992 | 0.952 | 1.000 | 0.916 | 0.948 |
| 3350 | 0.943 | 0.914 | 0.992 | 0.952 | 1.000 | 0.916 | 0.952 |
| 3360 | 0.951 | 0.909 | 0.992 | 0.952 | 1.000 | 0.916 | 0.948 |
| 3370 | 0.951 | 0.909 | 0.992 | 0.952 | 1.000 | 0.916 | 0.948 |
| 3380 | 0.951 | 0.909 | 0.992 | 0.952 | 1.000 | 0.916 | 0.948 |
| 3390 | 0.951 | 0.909 | 0.992 | 0.952 | 1.000 | 0.916 | 0.948 |
| 3400 | 0.951 | 0.909 | 0.992 | 0.952 | 1.000 | 0.916 | 0.948 |
| 3410 | 0.951 | 0.909 | 0.992 | 0.948 | 1.000 | 0.916 | 0.956 |
| 3420 | 0.951 | 0.914 | 0.992 | 0.952 | 1.000 | 0.916 | 0.956 |
| 3430 | 0.951 | 0.914 | 0.992 | 0.952 | 1.000 | 0.916 | 0.952 |
| 3440 | 0.951 | 0.914 | 0.992 | 0.952 | 1.000 | 0.916 | 0.952 |
| 3450 | 0.951 | 0.914 | 0.992 | 0.952 | 1.000 | 0.916 | 0.952 |
| 3460 | 0.951 | 0.909 | 0.992 | 0.952 | 1.000 | 0.916 | 0.952 |
| 3470 | 0.951 | 0.914 | 0.992 | 0.952 | 1.000 | 0.916 | 0.952 |
| 3480 | 0.951 | 0.914 | 0.992 | 0.952 | 1.000 | 0.916 | 0.952 |
| 3490 | 0.951 | 0.914 | 0.992 | 0.957 | 1.000 | 0.916 | 0.952 |
| 3500 | 0.951 | 0.914 | 0.992 | 0.957 | 1.000 | 0.916 | 0.948 |
| 3510 | 0.951 | 0.914 | 0.992 | 0.957 | 1.000 | 0.916 | 0.948 |
| 3520 | 0.947 | 0.914 | 0.992 | 0.957 | 1.000 | 0.916 | 0.948 |
| 3530 | 0.947 | 0.909 | 0.992 | 0.957 | 1.000 | 0.916 | 0.944 |
| 3540 | 0.947 | 0.909 | 0.992 | 0.957 | 0.996 | 0.916 | 0.952 |
| 3550 | 0.947 | 0.909 | 0.992 | 0.957 | 0.996 | 0.916 | 0.952 |
| 3560 | 0.947 | 0.914 | 0.992 | 0.957 | 1.000 | 0.916 | 0.952 |
| 3570 | 0.947 | 0.909 | 0.992 | 0.957 | 1.000 | 0.916 | 0.948 |
| 3580 | 0.947 | 0.914 | 0.992 | 0.957 | 1.000 | 0.916 | 0.948 |
| 3590 | 0.947 | 0.914 | 0.992 | 0.957 | 0.996 | 0.916 | 0.952 |
| 3600 | 0.947 | 0.914 | 0.992 | 0.952 | 0.996 | 0.916 | 0.952 |
| 3610 | 0.947 | 0.914 | 0.992 | 0.952 | 0.996 | 0.916 | 0.952 |
| 3620 | 0.947 | 0.914 | 0.988 | 0.952 | 0.996 | 0.916 | 0.952 |
| 3630 | 0.951 | 0.914 | 0.992 | 0.952 | 0.996 | 0.916 | 0.952 |
| 3640 | 0.947 | 0.914 | 0.988 | 0.952 | 0.996 | 0.916 | 0.952 |
| 3650 | 0.947 | 0.914 | 0.988 | 0.952 | 0.996 | 0.916 | 0.952 |
| 3660 | 0.947 | 0.914 | 0.988 | 0.952 | 0.996 | 0.916 | 0.952 |
| 3670 | 0.951 | 0.918 | 0.992 | 0.952 | 0.996 | 0.911 | 0.952 |
| 3680 | 0.951 | 0.918 | 0.992 | 0.957 | 0.996 | 0.911 | 0.952 |
| 3690 | 0.951 | 0.918 | 0.988 | 0.957 | 0.996 | 0.911 | 0.952 |
| 3700 | 0.955 | 0.918 | 0.992 | 0.952 | 0.996 | 0.911 | 0.952 |
| 3710 | 0.951 | 0.922 | 0.992 | 0.957 | 0.996 | 0.911 | 0.956 |
| 3720 | 0.951 | 0.922 | 0.992 | 0.957 | 0.996 | 0.911 | 0.952 |
| 3730 | 0.951 | 0.922 | 0.992 | 0.957 | 0.996 | 0.911 | 0.952 |
| 3740 | 0.955 | 0.922 | 0.992 | 0.957 | 0.996 | 0.907 | 0.952 |
| 3750 | 0.951 | 0.922 | 0.992 | 0.957 | 0.996 | 0.911 | 0.952 |
| 3760 | 0.947 | 0.922 | 0.992 | 0.957 | 0.996 | 0.911 | 0.952 |
| 3770 | 0.951 | 0.922 | 0.992 | 0.952 | 0.996 | 0.911 | 0.952 |
| 3780 | 0.951 | 0.922 | 0.992 | 0.952 | 0.996 | 0.911 | 0.956 |
| 3790 | 0.951 | 0.918 | 0.992 | 0.957 | 0.996 | 0.911 | 0.956 |
| 3800 | 0.951 | 0.922 | 0.992 | 0.952 | 0.996 | 0.911 | 0.952 |
| 3810 | 0.951 | 0.922 | 0.992 | 0.952 | 0.996 | 0.911 | 0.952 |
| 3820 | 0.951 | 0.922 | 0.992 | 0.952 | 0.996 | 0.911 | 0.952 |
| 3830 | 0.955 | 0.918 | 0.992 | 0.952 | 0.996 | 0.911 | 0.956 |
| 3840 | 0.951 | 0.922 | 0.992 | 0.952 | 0.996 | 0.911 | 0.952 |
| 3850 | 0.947 | 0.918 | 0.992 | 0.952 | 0.996 | 0.911 | 0.960 |
| 3860 | 0.951 | 0.914 | 0.992 | 0.952 | 0.996 | 0.911 | 0.956 |
| 3870 | 0.951 | 0.922 | 0.992 | 0.952 | 0.996 | 0.911 | 0.956 |
| 3880 | 0.951 | 0.922 | 0.992 | 0.952 | 0.996 | 0.911 | 0.952 |
| 3890 | 0.947 | 0.922 | 0.992 | 0.952 | 0.996 | 0.911 | 0.952 |
| 3900 | 0.951 | 0.918 | 0.992 | 0.952 | 0.996 | 0.907 | 0.956 |
| 3910 | 0.951 | 0.909 | 0.992 | 0.952 | 0.996 | 0.907 | 0.956 |
| 3920 | 0.951 | 0.918 | 0.992 | 0.952 | 0.996 | 0.907 | 0.952 |
| 3930 | 0.951 | 0.909 | 0.992 | 0.948 | 0.996 | 0.907 | 0.956 |
| 3940 | 0.951 | 0.914 | 0.992 | 0.948 | 0.996 | 0.902 | 0.956 |
| 3950 | 0.955 | 0.914 | 0.992 | 0.948 | 0.996 | 0.902 | 0.952 |
| 3960 | 0.951 | 0.918 | 0.992 | 0.948 | 0.996 | 0.902 | 0.956 |
| 3970 | 0.951 | 0.914 | 0.992 | 0.952 | 0.996 | 0.902 | 0.956 |
| 3980 | 0.951 | 0.914 | 0.992 | 0.948 | 0.996 | 0.902 | 0.956 |
| 3990 | 0.951 | 0.914 | 0.992 | 0.948 | 0.996 | 0.902 | 0.960 |
| 4000 | 0.951 | 0.914 | 0.992 | 0.948 | 0.996 | 0.902 | 0.960 |
| 4010 | 0.951 | 0.918 | 0.992 | 0.948 | 0.996 | 0.902 | 0.960 |
| 4020 | 0.951 | 0.918 | 0.992 | 0.948 | 0.996 | 0.902 | 0.960 |
| 4030 | 0.951 | 0.922 | 0.992 | 0.948 | 0.996 | 0.902 | 0.960 |
| 4040 | 0.947 | 0.922 | 0.992 | 0.948 | 0.996 | 0.902 | 0.968 |
| 4050 | 0.951 | 0.922 | 0.992 | 0.948 | 0.996 | 0.902 | 0.964 |
| 4060 | 0.951 | 0.922 | 0.992 | 0.948 | 0.996 | 0.898 | 0.964 |
| 4070 | 0.951 | 0.918 | 0.992 | 0.952 | 0.996 | 0.898 | 0.964 |
| 4080 | 0.951 | 0.918 | 0.992 | 0.952 | 0.996 | 0.902 | 0.960 |
| 4090 | 0.947 | 0.918 | 0.992 | 0.952 | 0.996 | 0.907 | 0.960 |
| 4100 | 0.947 | 0.918 | 0.992 | 0.948 | 0.996 | 0.902 | 0.960 |
| 4110 | 0.947 | 0.922 | 0.992 | 0.948 | 0.996 | 0.902 | 0.964 |
| 4120 | 0.947 | 0.922 | 0.992 | 0.952 | 0.996 | 0.902 | 0.964 |
| 4130 | 0.947 | 0.922 | 0.992 | 0.952 | 0.996 | 0.902 | 0.964 |
| 4140 | 0.947 | 0.930 | 0.992 | 0.952 | 0.996 | 0.902 | 0.964 |
| 4150 | 0.947 | 0.926 | 0.992 | 0.952 | 0.996 | 0.902 | 0.964 |
| 4160 | 0.951 | 0.922 | 0.992 | 0.952 | 0.996 | 0.898 | 0.964 |
| 4170 | 0.951 | 0.926 | 0.992 | 0.952 | 0.996 | 0.898 | 0.964 |
| 4180 | 0.951 | 0.926 | 0.992 | 0.952 | 0.996 | 0.898 | 0.964 |
| 4190 | 0.951 | 0.926 | 0.992 | 0.952 | 0.996 | 0.898 | 0.964 |
| 4200 | 0.951 | 0.926 | 0.992 | 0.952 | 0.996 | 0.898 | 0.964 |
| 4210 | 0.951 | 0.922 | 0.992 | 0.952 | 0.996 | 0.898 | 0.964 |
| 4220 | 0.951 | 0.922 | 0.992 | 0.952 | 0.996 | 0.898 | 0.964 |
| 4230 | 0.951 | 0.922 | 0.992 | 0.952 | 0.996 | 0.902 | 0.964 |
| 4240 | 0.951 | 0.922 | 0.992 | 0.952 | 0.996 | 0.902 | 0.964 |
| 4250 | 0.951 | 0.918 | 0.992 | 0.952 | 0.996 | 0.902 | 0.964 |
| 4260 | 0.951 | 0.922 | 0.992 | 0.952 | 0.996 | 0.898 | 0.964 |
| 4270 | 0.951 | 0.918 | 0.992 | 0.952 | 0.996 | 0.902 | 0.964 |
| 4280 | 0.951 | 0.918 | 0.992 | 0.952 | 0.996 | 0.898 | 0.964 |
| 4290 | 0.951 | 0.918 | 0.992 | 0.948 | 0.996 | 0.898 | 0.964 |
| 4300 | 0.951 | 0.918 | 0.992 | 0.948 | 0.996 | 0.898 | 0.964 |
| 4310 | 0.951 | 0.918 | 0.992 | 0.948 | 0.996 | 0.898 | 0.964 |
| 4320 | 0.951 | 0.918 | 0.992 | 0.952 | 0.996 | 0.898 | 0.960 |
| 4330 | 0.951 | 0.914 | 0.992 | 0.952 | 0.996 | 0.898 | 0.960 |
| 4340 | 0.951 | 0.914 | 0.992 | 0.952 | 0.996 | 0.898 | 0.956 |
| 4350 | 0.951 | 0.922 | 0.992 | 0.952 | 0.996 | 0.898 | 0.960 |
| 4360 | 0.951 | 0.918 | 0.992 | 0.952 | 0.996 | 0.898 | 0.956 |
| 4370 | 0.951 | 0.918 | 0.992 | 0.952 | 0.996 | 0.898 | 0.960 |
| 4380 | 0.951 | 0.922 | 0.992 | 0.952 | 0.996 | 0.898 | 0.956 |
| 4390 | 0.951 | 0.922 | 0.992 | 0.952 | 0.996 | 0.898 | 0.956 |
| 4400 | 0.951 | 0.918 | 0.992 | 0.952 | 0.996 | 0.898 | 0.956 |
| 4410 | 0.951 | 0.922 | 0.992 | 0.952 | 0.996 | 0.898 | 0.956 |
| 4420 | 0.951 | 0.922 | 0.992 | 0.952 | 0.996 | 0.898 | 0.956 |
| 4430 | 0.951 | 0.918 | 0.992 | 0.952 | 0.996 | 0.898 | 0.956 |
| 4440 | 0.951 | 0.918 | 0.992 | 0.952 | 0.996 | 0.898 | 0.956 |
| 4450 | 0.951 | 0.922 | 0.992 | 0.952 | 0.996 | 0.898 | 0.956 |
| 4460 | 0.951 | 0.922 | 0.992 | 0.952 | 0.996 | 0.898 | 0.956 |
| 4470 | 0.955 | 0.914 | 0.992 | 0.952 | 0.996 | 0.898 | 0.956 |
| 4480 | 0.955 | 0.914 | 0.992 | 0.952 | 0.996 | 0.898 | 0.956 |
| 4490 | 0.947 | 0.918 | 0.992 | 0.952 | 0.996 | 0.898 | 0.956 |
| 4500 | 0.947 | 0.918 | 0.992 | 0.952 | 0.996 | 0.898 | 0.956 |
| 4510 | 0.947 | 0.922 | 0.992 | 0.952 | 0.996 | 0.898 | 0.956 |
| 4520 | 0.947 | 0.922 | 0.992 | 0.952 | 0.996 | 0.898 | 0.956 |
| 4530 | 0.947 | 0.922 | 0.992 | 0.952 | 0.996 | 0.898 | 0.956 |
| 4540 | 0.947 | 0.922 | 0.992 | 0.952 | 0.996 | 0.898 | 0.956 |
| 4550 | 0.947 | 0.922 | 0.992 | 0.952 | 0.996 | 0.898 | 0.956 |
| 4560 | 0.947 | 0.918 | 0.992 | 0.952 | 0.996 | 0.898 | 0.956 |
| 4570 | 0.947 | 0.914 | 0.992 | 0.952 | 0.996 | 0.898 | 0.956 |
| 4580 | 0.947 | 0.914 | 0.992 | 0.952 | 0.996 | 0.898 | 0.956 |
| 4590 | 0.947 | 0.914 | 0.992 | 0.952 | 0.996 | 0.898 | 0.960 |
| 4600 | 0.947 | 0.914 | 0.992 | 0.952 | 0.996 | 0.898 | 0.960 |
| 4610 | 0.947 | 0.914 | 0.992 | 0.952 | 0.996 | 0.898 | 0.960 |
| 4620 | 0.947 | 0.914 | 0.992 | 0.952 | 0.996 | 0.898 | 0.960 |
| 4630 | 0.947 | 0.914 | 0.992 | 0.952 | 0.996 | 0.898 | 0.964 |
| 4640 | 0.947 | 0.914 | 0.992 | 0.952 | 0.996 | 0.898 | 0.964 |
| 4650 | 0.947 | 0.918 | 0.992 | 0.952 | 0.996 | 0.898 | 0.964 |
| 4660 | 0.947 | 0.922 | 0.992 | 0.952 | 0.996 | 0.898 | 0.964 |
| 4670 | 0.947 | 0.918 | 0.992 | 0.952 | 0.996 | 0.898 | 0.964 |
| 4680 | 0.947 | 0.918 | 0.992 | 0.952 | 0.996 | 0.898 | 0.964 |
| 4690 | 0.951 | 0.918 | 0.992 | 0.952 | 0.996 | 0.898 | 0.964 |
| 4700 | 0.951 | 0.918 | 0.992 | 0.952 | 0.996 | 0.898 | 0.964 |
| 4710 | 0.951 | 0.918 | 0.992 | 0.952 | 0.996 | 0.898 | 0.964 |
| 4720 | 0.951 | 0.918 | 0.992 | 0.952 | 0.996 | 0.898 | 0.964 |
| 4730 | 0.947 | 0.918 | 0.992 | 0.952 | 0.996 | 0.898 | 0.964 |
| 4740 | 0.947 | 0.918 | 0.992 | 0.952 | 0.996 | 0.898 | 0.964 |
| 4750 | 0.947 | 0.922 | 0.992 | 0.952 | 0.996 | 0.898 | 0.964 |
| 4760 | 0.947 | 0.922 | 0.992 | 0.952 | 0.996 | 0.898 | 0.964 |
| 4770 | 0.947 | 0.918 | 0.992 | 0.952 | 0.996 | 0.898 | 0.960 |
| 4780 | 0.947 | 0.918 | 0.988 | 0.952 | 0.996 | 0.902 | 0.960 |
| 4790 | 0.943 | 0.922 | 0.992 | 0.952 | 0.996 | 0.898 | 0.956 |
| 4800 | 0.947 | 0.922 | 0.988 | 0.952 | 0.996 | 0.902 | 0.964 |
| 4810 | 0.947 | 0.922 | 0.988 | 0.952 | 0.996 | 0.902 | 0.960 |
| 4820 | 0.951 | 0.922 | 0.988 | 0.952 | 0.996 | 0.902 | 0.960 |
| 4830 | 0.951 | 0.922 | 0.988 | 0.952 | 0.996 | 0.902 | 0.960 |
| 4840 | 0.951 | 0.918 | 0.988 | 0.952 | 0.996 | 0.902 | 0.960 |
| 4850 | 0.951 | 0.918 | 0.988 | 0.952 | 0.996 | 0.902 | 0.960 |
| 4860 | 0.951 | 0.918 | 0.988 | 0.952 | 0.996 | 0.902 | 0.960 |
| 4870 | 0.951 | 0.914 | 0.988 | 0.952 | 0.996 | 0.902 | 0.960 |
| 4880 | 0.951 | 0.914 | 0.988 | 0.952 | 0.996 | 0.902 | 0.960 |
| 4890 | 0.951 | 0.914 | 0.988 | 0.952 | 0.996 | 0.902 | 0.956 |
| 4900 | 0.951 | 0.914 | 0.988 | 0.952 | 0.996 | 0.902 | 0.960 |
| 4910 | 0.951 | 0.914 | 0.988 | 0.952 | 0.996 | 0.902 | 0.960 |
| 4920 | 0.951 | 0.914 | 0.988 | 0.952 | 0.996 | 0.902 | 0.960 |
| 4930 | 0.951 | 0.918 | 0.988 | 0.952 | 0.996 | 0.902 | 0.960 |
| 4940 | 0.955 | 0.918 | 0.988 | 0.952 | 0.996 | 0.902 | 0.956 |
| 4950 | 0.951 | 0.918 | 0.988 | 0.952 | 0.996 | 0.902 | 0.960 |
| 4960 | 0.955 | 0.918 | 0.988 | 0.952 | 0.996 | 0.902 | 0.956 |
| 4970 | 0.955 | 0.922 | 0.988 | 0.952 | 0.996 | 0.902 | 0.952 |
| 4980 | 0.959 | 0.922 | 0.988 | 0.952 | 0.996 | 0.902 | 0.952 |
| 4990 | 0.959 | 0.918 | 0.988 | 0.952 | 0.996 | 0.902 | 0.952 |
| 5000 | 0.959 | 0.918 | 0.988 | 0.952 | 0.996 | 0.902 | 0.952 |
| 5010 | 0.959 | 0.914 | 0.988 | 0.952 | 0.996 | 0.898 | 0.952 |
| 5020 | 0.959 | 0.909 | 0.988 | 0.952 | 0.996 | 0.902 | 0.956 |
| 5030 | 0.959 | 0.909 | 0.988 | 0.952 | 0.996 | 0.898 | 0.952 |
| 5040 | 0.959 | 0.909 | 0.988 | 0.952 | 0.996 | 0.898 | 0.956 |
| 5050 | 0.959 | 0.909 | 0.988 | 0.952 | 0.996 | 0.898 | 0.956 |
| 5060 | 0.959 | 0.909 | 0.988 | 0.952 | 0.996 | 0.898 | 0.956 |
| 5070 | 0.959 | 0.909 | 0.988 | 0.952 | 0.996 | 0.898 | 0.956 |
| 5080 | 0.959 | 0.909 | 0.988 | 0.952 | 0.996 | 0.898 | 0.956 |
| 5090 | 0.959 | 0.909 | 0.988 | 0.952 | 0.996 | 0.898 | 0.956 |
| 5100 | 0.959 | 0.909 | 0.992 | 0.952 | 0.996 | 0.893 | 0.956 |
| 5110 | 0.955 | 0.914 | 0.992 | 0.952 | 0.996 | 0.893 | 0.956 |
| 5120 | 0.955 | 0.914 | 0.992 | 0.952 | 0.996 | 0.893 | 0.956 |
| 5130 | 0.955 | 0.914 | 0.992 | 0.952 | 0.996 | 0.893 | 0.960 |
| 5140 | 0.959 | 0.909 | 0.992 | 0.952 | 0.996 | 0.893 | 0.956 |
| 5150 | 0.955 | 0.909 | 0.992 | 0.952 | 0.996 | 0.898 | 0.952 |
| 5160 | 0.955 | 0.909 | 0.992 | 0.952 | 0.996 | 0.898 | 0.956 |
| 5170 | 0.955 | 0.909 | 0.992 | 0.952 | 0.996 | 0.898 | 0.956 |
| 5180 | 0.955 | 0.909 | 0.992 | 0.952 | 0.996 | 0.898 | 0.960 |
| 5190 | 0.955 | 0.914 | 0.992 | 0.952 | 0.996 | 0.898 | 0.960 |
| 5200 | 0.955 | 0.909 | 0.992 | 0.952 | 0.996 | 0.898 | 0.960 |
| 5210 | 0.955 | 0.909 | 0.992 | 0.952 | 0.996 | 0.898 | 0.956 |
| 5220 | 0.955 | 0.909 | 0.992 | 0.952 | 0.996 | 0.898 | 0.956 |
| 5230 | 0.955 | 0.909 | 0.992 | 0.952 | 0.996 | 0.898 | 0.952 |
| 5240 | 0.955 | 0.905 | 0.992 | 0.952 | 0.996 | 0.898 | 0.952 |
| 5250 | 0.955 | 0.905 | 0.992 | 0.952 | 0.996 | 0.898 | 0.952 |
| 5260 | 0.955 | 0.905 | 0.992 | 0.952 | 0.996 | 0.898 | 0.956 |
| 5270 | 0.955 | 0.905 | 0.992 | 0.952 | 0.996 | 0.898 | 0.956 |
| 5280 | 0.955 | 0.905 | 0.992 | 0.952 | 0.996 | 0.898 | 0.952 |
| 5290 | 0.955 | 0.914 | 0.992 | 0.952 | 0.996 | 0.898 | 0.952 |
| 5300 | 0.955 | 0.914 | 0.992 | 0.952 | 0.996 | 0.898 | 0.948 |
| 5310 | 0.955 | 0.909 | 0.992 | 0.952 | 0.996 | 0.898 | 0.948 |
| 5320 | 0.955 | 0.909 | 0.992 | 0.952 | 0.996 | 0.898 | 0.948 |
| 5330 | 0.955 | 0.905 | 0.992 | 0.952 | 0.996 | 0.902 | 0.956 |
| 5340 | 0.955 | 0.905 | 0.992 | 0.952 | 0.996 | 0.898 | 0.956 |
| 5350 | 0.955 | 0.905 | 0.992 | 0.952 | 0.996 | 0.898 | 0.952 |
| 5360 | 0.955 | 0.905 | 0.992 | 0.952 | 0.996 | 0.898 | 0.952 |
| 5370 | 0.955 | 0.909 | 0.992 | 0.952 | 0.996 | 0.902 | 0.952 |
| 5380 | 0.955 | 0.905 | 0.992 | 0.952 | 0.996 | 0.902 | 0.952 |
| 5390 | 0.959 | 0.901 | 0.992 | 0.952 | 0.996 | 0.902 | 0.952 |
| 5400 | 0.959 | 0.905 | 0.992 | 0.948 | 0.996 | 0.902 | 0.948 |
| 5410 | 0.955 | 0.905 | 0.992 | 0.948 | 0.996 | 0.902 | 0.948 |
| 5420 | 0.959 | 0.909 | 0.992 | 0.948 | 0.996 | 0.898 | 0.948 |
| 5430 | 0.959 | 0.905 | 0.992 | 0.948 | 0.996 | 0.898 | 0.948 |
| 5440 | 0.963 | 0.901 | 0.992 | 0.952 | 0.996 | 0.898 | 0.952 |
| 5450 | 0.963 | 0.901 | 0.992 | 0.952 | 0.996 | 0.898 | 0.952 |
| 5460 | 0.959 | 0.905 | 0.992 | 0.952 | 0.996 | 0.898 | 0.952 |
| 5470 | 0.955 | 0.905 | 0.992 | 0.952 | 0.996 | 0.902 | 0.952 |
| 5480 | 0.959 | 0.901 | 0.992 | 0.952 | 0.996 | 0.902 | 0.952 |
| 5490 | 0.959 | 0.905 | 0.992 | 0.952 | 0.996 | 0.898 | 0.956 |
| 5500 | 0.959 | 0.905 | 0.992 | 0.952 | 0.996 | 0.898 | 0.956 |
| 5510 | 0.959 | 0.905 | 0.992 | 0.952 | 0.996 | 0.898 | 0.952 |
| 5520 | 0.959 | 0.905 | 0.992 | 0.952 | 0.996 | 0.898 | 0.956 |
| 5530 | 0.959 | 0.905 | 0.992 | 0.952 | 0.996 | 0.893 | 0.952 |
| 5540 | 0.959 | 0.905 | 0.992 | 0.952 | 0.996 | 0.893 | 0.956 |
| 5550 | 0.959 | 0.909 | 0.992 | 0.952 | 0.996 | 0.893 | 0.952 |
| 5560 | 0.959 | 0.909 | 0.992 | 0.952 | 0.996 | 0.893 | 0.956 |
| 5570 | 0.967 | 0.901 | 0.992 | 0.952 | 0.996 | 0.893 | 0.952 |
| 5580 | 0.967 | 0.901 | 0.992 | 0.952 | 0.996 | 0.893 | 0.952 |
| 5590 | 0.967 | 0.905 | 0.992 | 0.952 | 0.996 | 0.893 | 0.948 |
| 5600 | 0.967 | 0.905 | 0.992 | 0.948 | 0.996 | 0.893 | 0.948 |
| 5610 | 0.963 | 0.905 | 0.992 | 0.948 | 0.996 | 0.893 | 0.948 |
| 5620 | 0.963 | 0.905 | 0.992 | 0.948 | 0.996 | 0.893 | 0.948 |
| 5630 | 0.963 | 0.909 | 0.992 | 0.948 | 0.996 | 0.889 | 0.948 |
| 5640 | 0.963 | 0.909 | 0.992 | 0.948 | 0.996 | 0.889 | 0.948 |
| 5650 | 0.963 | 0.909 | 0.992 | 0.948 | 0.996 | 0.889 | 0.948 |
| 5660 | 0.963 | 0.909 | 0.992 | 0.948 | 0.996 | 0.889 | 0.948 |
| 5670 | 0.963 | 0.909 | 0.992 | 0.948 | 0.996 | 0.889 | 0.948 |
| 5680 | 0.963 | 0.909 | 0.992 | 0.948 | 0.996 | 0.889 | 0.948 |
| 5690 | 0.963 | 0.905 | 0.992 | 0.948 | 0.996 | 0.889 | 0.948 |
| 5700 | 0.963 | 0.905 | 0.992 | 0.948 | 0.996 | 0.889 | 0.948 |
| 5710 | 0.963 | 0.909 | 0.992 | 0.948 | 0.996 | 0.889 | 0.948 |
| 5720 | 0.963 | 0.909 | 0.992 | 0.948 | 0.996 | 0.889 | 0.948 |
| 5730 | 0.963 | 0.909 | 0.992 | 0.948 | 0.996 | 0.889 | 0.948 |
| 5740 | 0.963 | 0.909 | 0.992 | 0.948 | 0.996 | 0.889 | 0.948 |
| 5750 | 0.963 | 0.914 | 0.992 | 0.948 | 0.996 | 0.889 | 0.948 |
| 5760 | 0.967 | 0.909 | 0.992 | 0.948 | 0.996 | 0.889 | 0.948 |
| 5770 | 0.967 | 0.914 | 0.992 | 0.948 | 0.996 | 0.889 | 0.948 |
| 5780 | 0.967 | 0.909 | 0.992 | 0.948 | 0.996 | 0.889 | 0.948 |
| 5790 | 0.967 | 0.914 | 0.992 | 0.948 | 0.996 | 0.889 | 0.948 |
| 5800 | 0.967 | 0.914 | 0.992 | 0.948 | 0.996 | 0.889 | 0.948 |
| 5810 | 0.967 | 0.914 | 0.992 | 0.948 | 0.996 | 0.889 | 0.948 |
| 5820 | 0.967 | 0.909 | 0.992 | 0.948 | 0.996 | 0.889 | 0.948 |
| 5830 | 0.967 | 0.909 | 0.992 | 0.948 | 0.996 | 0.889 | 0.948 |
| 5840 | 0.967 | 0.909 | 0.992 | 0.948 | 0.996 | 0.889 | 0.948 |
| 5850 | 0.967 | 0.909 | 0.992 | 0.948 | 0.996 | 0.889 | 0.948 |
| 5860 | 0.967 | 0.905 | 0.992 | 0.948 | 0.996 | 0.889 | 0.948 |
| 5870 | 0.967 | 0.901 | 0.992 | 0.948 | 0.996 | 0.893 | 0.948 |
| 5880 | 0.967 | 0.901 | 0.992 | 0.948 | 0.996 | 0.893 | 0.948 |
| 5890 | 0.967 | 0.901 | 0.992 | 0.948 | 0.996 | 0.893 | 0.948 |
| 5900 | 0.967 | 0.901 | 0.992 | 0.948 | 0.996 | 0.893 | 0.948 |
| 5910 | 0.967 | 0.901 | 0.992 | 0.948 | 0.996 | 0.893 | 0.948 |
| 5920 | 0.967 | 0.901 | 0.992 | 0.948 | 0.996 | 0.889 | 0.948 |
| 5930 | 0.967 | 0.905 | 0.992 | 0.948 | 0.996 | 0.889 | 0.948 |
| 5940 | 0.967 | 0.905 | 0.992 | 0.948 | 0.996 | 0.889 | 0.948 |
| 5950 | 0.967 | 0.905 | 0.992 | 0.948 | 0.996 | 0.889 | 0.948 |
| 5960 | 0.967 | 0.905 | 0.992 | 0.948 | 0.996 | 0.889 | 0.948 |
| 5970 | 0.963 | 0.905 | 0.992 | 0.948 | 0.996 | 0.889 | 0.948 |
| 5980 | 0.963 | 0.905 | 0.992 | 0.948 | 0.996 | 0.889 | 0.948 |
| 5990 | 0.963 | 0.909 | 0.992 | 0.948 | 0.996 | 0.889 | 0.948 |
| 6000 | 0.963 | 0.909 | 0.992 | 0.948 | 0.996 | 0.889 | 0.948 |
| 6010 | 0.963 | 0.905 | 0.992 | 0.948 | 0.996 | 0.889 | 0.948 |
| 6020 | 0.963 | 0.905 | 0.992 | 0.948 | 0.996 | 0.889 | 0.948 |
| 6030 | 0.963 | 0.905 | 0.992 | 0.948 | 0.996 | 0.889 | 0.948 |
| 6040 | 0.967 | 0.901 | 0.992 | 0.948 | 0.996 | 0.889 | 0.948 |
| 6050 | 0.963 | 0.905 | 0.992 | 0.948 | 0.996 | 0.889 | 0.948 |
| 6060 | 0.963 | 0.905 | 0.992 | 0.948 | 0.996 | 0.889 | 0.948 |
| 6070 | 0.963 | 0.901 | 0.992 | 0.948 | 0.996 | 0.889 | 0.948 |
| 6080 | 0.963 | 0.905 | 0.992 | 0.948 | 0.996 | 0.889 | 0.948 |
| 6090 | 0.963 | 0.901 | 0.992 | 0.948 | 0.996 | 0.893 | 0.948 |
| 6100 | 0.963 | 0.905 | 0.992 | 0.948 | 0.996 | 0.893 | 0.948 |
| 6110 | 0.963 | 0.905 | 0.992 | 0.952 | 0.996 | 0.893 | 0.948 |
| 6120 | 0.963 | 0.905 | 0.992 | 0.952 | 0.996 | 0.893 | 0.948 |
| 6130 | 0.963 | 0.905 | 0.992 | 0.952 | 0.996 | 0.893 | 0.948 |
| 6140 | 0.963 | 0.905 | 0.992 | 0.952 | 0.996 | 0.893 | 0.948 |
| 6150 | 0.959 | 0.905 | 0.992 | 0.952 | 0.996 | 0.889 | 0.948 |
| 6160 | 0.959 | 0.909 | 0.992 | 0.952 | 0.996 | 0.884 | 0.948 |
| 6170 | 0.959 | 0.905 | 0.992 | 0.952 | 0.996 | 0.893 | 0.948 |
| 6180 | 0.955 | 0.914 | 0.992 | 0.952 | 0.996 | 0.889 | 0.948 |
| 6190 | 0.959 | 0.914 | 0.992 | 0.952 | 0.996 | 0.889 | 0.948 |
| 6200 | 0.959 | 0.914 | 0.992 | 0.952 | 0.996 | 0.884 | 0.948 |
| 6210 | 0.955 | 0.914 | 0.992 | 0.952 | 0.996 | 0.880 | 0.948 |
| 6220 | 0.959 | 0.909 | 0.992 | 0.952 | 0.996 | 0.880 | 0.944 |
| 6230 | 0.959 | 0.909 | 0.992 | 0.952 | 0.996 | 0.880 | 0.944 |
| 6240 | 0.959 | 0.909 | 0.992 | 0.952 | 0.996 | 0.880 | 0.948 |
| 6250 | 0.959 | 0.909 | 0.992 | 0.952 | 0.996 | 0.880 | 0.952 |
| 6260 | 0.955 | 0.918 | 0.992 | 0.952 | 0.996 | 0.884 | 0.948 |
| 6270 | 0.955 | 0.918 | 0.992 | 0.952 | 0.996 | 0.884 | 0.948 |
| 6280 | 0.955 | 0.918 | 0.992 | 0.952 | 0.996 | 0.884 | 0.944 |
| 6290 | 0.955 | 0.918 | 0.992 | 0.952 | 0.996 | 0.884 | 0.948 |
| 6300 | 0.951 | 0.914 | 0.992 | 0.952 | 0.996 | 0.884 | 0.948 |
| 6310 | 0.951 | 0.918 | 0.992 | 0.952 | 0.996 | 0.884 | 0.948 |
| 6320 | 0.955 | 0.914 | 0.992 | 0.952 | 0.996 | 0.889 | 0.940 |
| 6330 | 0.955 | 0.909 | 0.992 | 0.952 | 0.996 | 0.889 | 0.940 |
| 6340 | 0.955 | 0.909 | 0.992 | 0.952 | 0.996 | 0.889 | 0.944 |
| 6350 | 0.959 | 0.897 | 0.992 | 0.952 | 0.996 | 0.889 | 0.944 |
| 6360 | 0.959 | 0.897 | 0.992 | 0.952 | 0.996 | 0.880 | 0.948 |
| 6370 | 0.955 | 0.897 | 0.992 | 0.952 | 0.996 | 0.880 | 0.948 |
| 6380 | 0.951 | 0.901 | 0.992 | 0.952 | 0.996 | 0.884 | 0.948 |
| 6390 | 0.951 | 0.901 | 0.992 | 0.952 | 0.996 | 0.884 | 0.952 |
| 6400 | 0.955 | 0.897 | 0.992 | 0.952 | 0.996 | 0.889 | 0.948 |
| 6410 | 0.959 | 0.897 | 0.992 | 0.952 | 0.996 | 0.889 | 0.948 |
| 6420 | 0.959 | 0.901 | 0.992 | 0.952 | 0.996 | 0.884 | 0.948 |
| 6430 | 0.959 | 0.897 | 0.992 | 0.952 | 0.996 | 0.889 | 0.944 |
| 6440 | 0.955 | 0.897 | 0.992 | 0.952 | 0.996 | 0.889 | 0.944 |
| 6450 | 0.955 | 0.897 | 0.992 | 0.952 | 0.996 | 0.889 | 0.948 |
| 6460 | 0.955 | 0.901 | 0.992 | 0.952 | 0.996 | 0.893 | 0.948 |
| 6470 | 0.959 | 0.901 | 0.992 | 0.952 | 0.996 | 0.893 | 0.948 |
| 6480 | 0.959 | 0.897 | 0.992 | 0.952 | 0.996 | 0.893 | 0.944 |
| 6490 | 0.959 | 0.901 | 0.992 | 0.952 | 0.996 | 0.893 | 0.948 |
| 6500 | 0.963 | 0.901 | 0.992 | 0.952 | 0.996 | 0.893 | 0.948 |
| 6510 | 0.959 | 0.897 | 0.992 | 0.952 | 0.996 | 0.893 | 0.944 |
| 6520 | 0.959 | 0.901 | 0.992 | 0.952 | 0.996 | 0.893 | 0.948 |
| 6530 | 0.963 | 0.901 | 0.992 | 0.952 | 0.996 | 0.893 | 0.948 |
| 6540 | 0.959 | 0.901 | 0.992 | 0.948 | 0.996 | 0.893 | 0.948 |
| 6550 | 0.959 | 0.901 | 0.992 | 0.948 | 0.996 | 0.893 | 0.940 |
| 6560 | 0.959 | 0.901 | 0.992 | 0.948 | 0.996 | 0.893 | 0.940 |
| 6570 | 0.955 | 0.909 | 0.992 | 0.948 | 0.996 | 0.893 | 0.936 |
| 6580 | 0.955 | 0.901 | 0.992 | 0.948 | 0.996 | 0.893 | 0.940 |
| 6590 | 0.955 | 0.901 | 0.992 | 0.948 | 0.996 | 0.889 | 0.944 |
| 6600 | 0.955 | 0.897 | 0.992 | 0.948 | 0.996 | 0.889 | 0.944 |
| 6610 | 0.955 | 0.901 | 0.992 | 0.948 | 0.996 | 0.889 | 0.940 |
| 6620 | 0.959 | 0.901 | 0.992 | 0.948 | 0.996 | 0.889 | 0.940 |
| 6630 | 0.955 | 0.901 | 0.992 | 0.948 | 0.996 | 0.889 | 0.944 |
| 6640 | 0.955 | 0.905 | 0.992 | 0.948 | 0.996 | 0.889 | 0.944 |
| 6650 | 0.955 | 0.905 | 0.992 | 0.948 | 0.996 | 0.889 | 0.944 |
| 6660 | 0.959 | 0.897 | 0.992 | 0.948 | 0.996 | 0.889 | 0.952 |
| 6670 | 0.959 | 0.897 | 0.992 | 0.948 | 0.996 | 0.889 | 0.952 |
| 6680 | 0.959 | 0.897 | 0.992 | 0.948 | 0.996 | 0.889 | 0.952 |
| 6690 | 0.959 | 0.893 | 0.992 | 0.948 | 0.996 | 0.889 | 0.952 |
| 6700 | 0.959 | 0.893 | 0.992 | 0.948 | 0.996 | 0.889 | 0.952 |
| 6710 | 0.959 | 0.893 | 0.992 | 0.948 | 0.996 | 0.889 | 0.952 |
| 6720 | 0.959 | 0.897 | 0.992 | 0.948 | 0.996 | 0.889 | 0.952 |
| 6730 | 0.955 | 0.901 | 0.992 | 0.948 | 0.996 | 0.889 | 0.952 |
| 6740 | 0.955 | 0.897 | 0.992 | 0.948 | 0.996 | 0.889 | 0.952 |
| 6750 | 0.955 | 0.897 | 0.992 | 0.948 | 0.996 | 0.889 | 0.952 |
| 6760 | 0.959 | 0.893 | 0.992 | 0.948 | 0.996 | 0.889 | 0.952 |
| 6770 | 0.959 | 0.893 | 0.992 | 0.948 | 0.996 | 0.889 | 0.952 |
| 6780 | 0.959 | 0.893 | 0.992 | 0.948 | 0.996 | 0.884 | 0.948 |
| 6790 | 0.959 | 0.893 | 0.992 | 0.948 | 0.996 | 0.889 | 0.948 |
| 6800 | 0.959 | 0.893 | 0.992 | 0.948 | 0.996 | 0.889 | 0.948 |
| 6810 | 0.959 | 0.893 | 0.992 | 0.948 | 0.996 | 0.889 | 0.948 |
| 6820 | 0.959 | 0.893 | 0.992 | 0.948 | 0.996 | 0.889 | 0.948 |
| 6830 | 0.959 | 0.893 | 0.992 | 0.948 | 0.996 | 0.884 | 0.948 |
| 6840 | 0.959 | 0.893 | 0.992 | 0.948 | 0.996 | 0.884 | 0.948 |
| 6850 | 0.959 | 0.897 | 0.992 | 0.948 | 0.996 | 0.884 | 0.948 |
| 6860 | 0.959 | 0.897 | 0.992 | 0.948 | 0.996 | 0.884 | 0.948 |
| 6870 | 0.959 | 0.897 | 0.992 | 0.948 | 0.996 | 0.884 | 0.948 |
| 6880 | 0.959 | 0.893 | 0.992 | 0.948 | 0.996 | 0.884 | 0.948 |
| 6890 | 0.955 | 0.901 | 0.992 | 0.948 | 0.996 | 0.884 | 0.948 |
| 6900 | 0.955 | 0.897 | 0.992 | 0.948 | 0.996 | 0.884 | 0.944 |
| 6910 | 0.955 | 0.897 | 0.992 | 0.948 | 0.996 | 0.884 | 0.944 |
| 6920 | 0.955 | 0.897 | 0.992 | 0.948 | 0.996 | 0.884 | 0.944 |
| 6930 | 0.955 | 0.897 | 0.992 | 0.948 | 0.996 | 0.884 | 0.944 |
| 6940 | 0.955 | 0.897 | 0.992 | 0.948 | 0.996 | 0.884 | 0.948 |
| 6950 | 0.955 | 0.897 | 0.992 | 0.948 | 0.996 | 0.884 | 0.948 |
| 6960 | 0.955 | 0.897 | 0.992 | 0.948 | 0.996 | 0.889 | 0.944 |
| 6970 | 0.955 | 0.897 | 0.992 | 0.948 | 0.996 | 0.889 | 0.944 |
| 6980 | 0.955 | 0.897 | 0.992 | 0.948 | 0.996 | 0.889 | 0.944 |
| 6990 | 0.955 | 0.893 | 0.992 | 0.948 | 0.996 | 0.893 | 0.948 |
| 7000 | 0.955 | 0.897 | 0.992 | 0.948 | 0.996 | 0.893 | 0.944 |
| 7010 | 0.955 | 0.893 | 0.992 | 0.948 | 0.996 | 0.893 | 0.948 |
| 7020 | 0.955 | 0.893 | 0.992 | 0.948 | 0.996 | 0.893 | 0.948 |
| 7030 | 0.955 | 0.901 | 0.992 | 0.948 | 0.996 | 0.893 | 0.944 |
| 7040 | 0.955 | 0.897 | 0.992 | 0.948 | 0.996 | 0.893 | 0.948 |
| 7050 | 0.955 | 0.897 | 0.992 | 0.948 | 0.996 | 0.893 | 0.948 |
| 7060 | 0.955 | 0.901 | 0.992 | 0.948 | 0.996 | 0.893 | 0.940 |
| 7070 | 0.955 | 0.897 | 0.992 | 0.948 | 0.996 | 0.893 | 0.940 |
| 7080 | 0.955 | 0.897 | 0.992 | 0.948 | 0.996 | 0.893 | 0.940 |
| 7090 | 0.955 | 0.897 | 0.992 | 0.948 | 0.996 | 0.893 | 0.944 |
| 7100 | 0.955 | 0.897 | 0.992 | 0.948 | 0.996 | 0.893 | 0.940 |
| 7110 | 0.955 | 0.901 | 0.992 | 0.948 | 0.996 | 0.889 | 0.940 |
| 7120 | 0.955 | 0.897 | 0.992 | 0.948 | 0.996 | 0.893 | 0.940 |
| 7130 | 0.951 | 0.901 | 0.992 | 0.948 | 0.996 | 0.893 | 0.940 |
| 7140 | 0.951 | 0.901 | 0.992 | 0.948 | 0.996 | 0.893 | 0.940 |
| 7150 | 0.955 | 0.901 | 0.992 | 0.948 | 0.996 | 0.889 | 0.940 |
| 7160 | 0.955 | 0.897 | 0.992 | 0.948 | 0.996 | 0.893 | 0.940 |
| 7170 | 0.955 | 0.897 | 0.992 | 0.948 | 0.996 | 0.893 | 0.940 |
| 7180 | 0.955 | 0.901 | 0.992 | 0.948 | 0.996 | 0.889 | 0.936 |
| 7190 | 0.955 | 0.901 | 0.992 | 0.948 | 0.996 | 0.889 | 0.940 |
| 7200 | 0.955 | 0.901 | 0.988 | 0.948 | 0.996 | 0.889 | 0.936 |
| 7210 | 0.955 | 0.901 | 0.992 | 0.948 | 0.996 | 0.889 | 0.936 |
| 7220 | 0.955 | 0.905 | 0.992 | 0.948 | 0.996 | 0.884 | 0.940 |
| 7230 | 0.955 | 0.901 | 0.992 | 0.948 | 0.996 | 0.880 | 0.944 |
| 7240 | 0.955 | 0.901 | 0.992 | 0.948 | 0.996 | 0.880 | 0.936 |
| 7250 | 0.955 | 0.905 | 0.992 | 0.948 | 0.996 | 0.880 | 0.940 |
| 7260 | 0.955 | 0.901 | 0.992 | 0.948 | 0.996 | 0.880 | 0.940 |
| 7270 | 0.951 | 0.909 | 0.992 | 0.948 | 0.996 | 0.880 | 0.944 |
| 7280 | 0.955 | 0.905 | 0.992 | 0.948 | 0.996 | 0.880 | 0.940 |
| 7290 | 0.955 | 0.905 | 0.992 | 0.948 | 0.996 | 0.880 | 0.940 |
| 7300 | 0.955 | 0.905 | 0.992 | 0.948 | 0.996 | 0.880 | 0.944 |
| 7310 | 0.955 | 0.905 | 0.992 | 0.948 | 0.996 | 0.880 | 0.944 |
| 7320 | 0.955 | 0.905 | 0.992 | 0.948 | 0.996 | 0.880 | 0.940 |
| 7330 | 0.955 | 0.909 | 0.992 | 0.948 | 0.996 | 0.880 | 0.940 |
| 7340 | 0.955 | 0.909 | 0.992 | 0.948 | 0.996 | 0.880 | 0.940 |
| 7350 | 0.955 | 0.909 | 0.988 | 0.948 | 0.996 | 0.884 | 0.940 |
| 7360 | 0.955 | 0.909 | 0.988 | 0.948 | 0.996 | 0.889 | 0.944 |
| 7370 | 0.955 | 0.909 | 0.988 | 0.948 | 0.996 | 0.884 | 0.944 |
| 7380 | 0.955 | 0.909 | 0.992 | 0.948 | 0.996 | 0.880 | 0.944 |
| 7390 | 0.955 | 0.909 | 0.992 | 0.948 | 0.996 | 0.880 | 0.944 |
| 7400 | 0.955 | 0.909 | 0.992 | 0.948 | 0.996 | 0.889 | 0.940 |
| 7410 | 0.951 | 0.914 | 0.992 | 0.948 | 0.996 | 0.889 | 0.944 |
| 7420 | 0.951 | 0.914 | 0.992 | 0.948 | 0.996 | 0.889 | 0.944 |
| 7430 | 0.955 | 0.909 | 0.988 | 0.948 | 0.996 | 0.893 | 0.944 |
| 7440 | 0.951 | 0.914 | 0.992 | 0.948 | 0.996 | 0.884 | 0.944 |
| 7450 | 0.955 | 0.909 | 0.992 | 0.948 | 0.996 | 0.889 | 0.944 |
| 7460 | 0.955 | 0.909 | 0.992 | 0.948 | 0.996 | 0.884 | 0.944 |
| 7470 | 0.955 | 0.909 | 0.992 | 0.948 | 0.996 | 0.889 | 0.944 |
| 7480 | 0.955 | 0.909 | 0.992 | 0.948 | 0.996 | 0.889 | 0.944 |
| 7490 | 0.955 | 0.909 | 0.992 | 0.948 | 0.996 | 0.884 | 0.944 |
| 7500 | 0.955 | 0.909 | 0.992 | 0.948 | 0.996 | 0.880 | 0.944 |
| 7510 | 0.951 | 0.914 | 0.992 | 0.948 | 0.996 | 0.889 | 0.944 |
| 7520 | 0.951 | 0.914 | 0.992 | 0.948 | 0.996 | 0.889 | 0.944 |
| 7530 | 0.951 | 0.914 | 0.992 | 0.948 | 0.996 | 0.889 | 0.940 |
| 7540 | 0.951 | 0.914 | 0.992 | 0.948 | 0.996 | 0.889 | 0.940 |
| 7550 | 0.951 | 0.914 | 0.992 | 0.948 | 0.996 | 0.889 | 0.940 |
| 7560 | 0.951 | 0.914 | 0.992 | 0.948 | 0.996 | 0.884 | 0.940 |
| 7570 | 0.951 | 0.914 | 0.992 | 0.948 | 0.996 | 0.889 | 0.940 |
| 7580 | 0.951 | 0.914 | 0.992 | 0.948 | 0.996 | 0.889 | 0.940 |
| 7590 | 0.955 | 0.909 | 0.992 | 0.948 | 0.996 | 0.884 | 0.940 |
| 7600 | 0.951 | 0.914 | 0.992 | 0.948 | 0.996 | 0.884 | 0.940 |
| 7610 | 0.951 | 0.914 | 0.992 | 0.948 | 0.996 | 0.889 | 0.940 |
| 7620 | 0.951 | 0.909 | 0.992 | 0.948 | 0.996 | 0.889 | 0.944 |
| 7630 | 0.951 | 0.909 | 0.992 | 0.948 | 0.996 | 0.889 | 0.944 |
| 7640 | 0.951 | 0.909 | 0.988 | 0.948 | 0.996 | 0.893 | 0.948 |
| 7650 | 0.951 | 0.909 | 0.992 | 0.948 | 0.996 | 0.884 | 0.948 |
| 7660 | 0.951 | 0.905 | 0.992 | 0.948 | 0.996 | 0.884 | 0.952 |
| 7670 | 0.955 | 0.897 | 0.992 | 0.948 | 0.996 | 0.889 | 0.952 |
| 7680 | 0.955 | 0.901 | 0.992 | 0.948 | 0.996 | 0.889 | 0.952 |
| 7690 | 0.955 | 0.901 | 0.992 | 0.948 | 0.996 | 0.889 | 0.952 |
| 7700 | 0.955 | 0.901 | 0.992 | 0.948 | 0.996 | 0.889 | 0.952 |
| 7710 | 0.951 | 0.901 | 0.992 | 0.948 | 0.996 | 0.889 | 0.952 |
| 7720 | 0.951 | 0.909 | 0.992 | 0.948 | 0.996 | 0.889 | 0.952 |
| 7730 | 0.951 | 0.909 | 0.992 | 0.948 | 0.996 | 0.889 | 0.944 |
| 7740 | 0.951 | 0.914 | 0.992 | 0.948 | 0.996 | 0.884 | 0.944 |
| 7750 | 0.951 | 0.901 | 0.992 | 0.948 | 0.996 | 0.884 | 0.948 |
| 7760 | 0.951 | 0.914 | 0.992 | 0.948 | 0.996 | 0.884 | 0.944 |
| 7770 | 0.951 | 0.909 | 0.992 | 0.948 | 0.996 | 0.884 | 0.944 |
| 7780 | 0.955 | 0.905 | 0.992 | 0.948 | 0.996 | 0.884 | 0.944 |
| 7790 | 0.955 | 0.909 | 0.992 | 0.948 | 0.996 | 0.880 | 0.940 |
| 7800 | 0.955 | 0.905 | 0.992 | 0.948 | 0.996 | 0.880 | 0.944 |
| 7810 | 0.955 | 0.905 | 0.992 | 0.948 | 0.996 | 0.880 | 0.944 |
| 7820 | 0.951 | 0.909 | 0.992 | 0.948 | 0.996 | 0.880 | 0.944 |
| 7830 | 0.955 | 0.905 | 0.992 | 0.948 | 0.996 | 0.880 | 0.944 |
| 7840 | 0.955 | 0.905 | 0.992 | 0.948 | 0.996 | 0.880 | 0.944 |
| 7850 | 0.955 | 0.905 | 0.992 | 0.948 | 0.996 | 0.880 | 0.948 |
| 7860 | 0.955 | 0.909 | 0.992 | 0.948 | 0.996 | 0.884 | 0.944 |
| 7870 | 0.955 | 0.909 | 0.992 | 0.948 | 0.996 | 0.884 | 0.944 |
| 7880 | 0.955 | 0.905 | 0.992 | 0.952 | 0.996 | 0.884 | 0.944 |
| 7890 | 0.955 | 0.901 | 0.992 | 0.952 | 0.996 | 0.884 | 0.948 |
| 7900 | 0.955 | 0.901 | 0.992 | 0.952 | 0.996 | 0.884 | 0.944 |
| 7910 | 0.955 | 0.901 | 0.992 | 0.952 | 0.996 | 0.884 | 0.944 |
| 7920 | 0.955 | 0.901 | 0.992 | 0.948 | 0.996 | 0.884 | 0.944 |
| 7930 | 0.951 | 0.905 | 0.992 | 0.948 | 0.996 | 0.884 | 0.944 |
| 7940 | 0.951 | 0.905 | 0.992 | 0.948 | 0.996 | 0.884 | 0.944 |
| 7950 | 0.951 | 0.905 | 0.992 | 0.948 | 0.996 | 0.889 | 0.944 |
| 7960 | 0.951 | 0.905 | 0.992 | 0.948 | 0.996 | 0.884 | 0.944 |
| 7970 | 0.951 | 0.905 | 0.992 | 0.948 | 0.996 | 0.884 | 0.944 |
| 7980 | 0.951 | 0.905 | 0.992 | 0.948 | 0.996 | 0.884 | 0.944 |
| 7990 | 0.951 | 0.905 | 0.992 | 0.948 | 0.996 | 0.884 | 0.944 |
| 8000 | 0.951 | 0.909 | 0.992 | 0.948 | 0.996 | 0.884 | 0.948 |
| 8010 | 0.951 | 0.905 | 0.992 | 0.948 | 0.996 | 0.884 | 0.952 |
| 8020 | 0.951 | 0.914 | 0.992 | 0.948 | 0.996 | 0.884 | 0.948 |
| 8030 | 0.951 | 0.909 | 0.992 | 0.948 | 0.996 | 0.880 | 0.948 |
| 8040 | 0.951 | 0.909 | 0.992 | 0.948 | 0.996 | 0.880 | 0.948 |
| 8050 | 0.951 | 0.909 | 0.992 | 0.948 | 0.996 | 0.880 | 0.944 |
| 8060 | 0.951 | 0.914 | 0.992 | 0.948 | 0.996 | 0.880 | 0.944 |
| 8070 | 0.951 | 0.914 | 0.992 | 0.948 | 0.996 | 0.880 | 0.948 |
| 8080 | 0.951 | 0.914 | 0.992 | 0.952 | 0.996 | 0.884 | 0.948 |
| 8090 | 0.947 | 0.909 | 0.992 | 0.952 | 0.996 | 0.884 | 0.948 |
| 8100 | 0.947 | 0.909 | 0.992 | 0.952 | 0.996 | 0.884 | 0.944 |
| 8110 | 0.947 | 0.909 | 0.992 | 0.952 | 0.996 | 0.884 | 0.944 |
| 8120 | 0.951 | 0.914 | 0.992 | 0.948 | 0.996 | 0.884 | 0.944 |
| 8130 | 0.951 | 0.914 | 0.992 | 0.948 | 0.996 | 0.884 | 0.944 |
| 8140 | 0.951 | 0.914 | 0.992 | 0.948 | 0.996 | 0.884 | 0.944 |
| 8150 | 0.951 | 0.914 | 0.992 | 0.948 | 0.996 | 0.884 | 0.944 |
| 8160 | 0.951 | 0.914 | 0.992 | 0.948 | 0.996 | 0.884 | 0.944 |
| 8170 | 0.951 | 0.914 | 0.992 | 0.948 | 0.996 | 0.884 | 0.944 |
| 8180 | 0.951 | 0.914 | 0.992 | 0.948 | 0.996 | 0.884 | 0.944 |
| 8190 | 0.951 | 0.914 | 0.992 | 0.952 | 0.996 | 0.889 | 0.944 |
| 8200 | 0.951 | 0.914 | 0.992 | 0.952 | 0.996 | 0.889 | 0.944 |
| 8210 | 0.951 | 0.905 | 0.992 | 0.952 | 0.996 | 0.889 | 0.944 |
| 8220 | 0.951 | 0.909 | 0.992 | 0.952 | 0.996 | 0.889 | 0.944 |
| 8230 | 0.951 | 0.914 | 0.992 | 0.952 | 0.996 | 0.889 | 0.944 |
| 8240 | 0.951 | 0.909 | 0.992 | 0.952 | 0.996 | 0.889 | 0.944 |
| 8250 | 0.951 | 0.909 | 0.992 | 0.952 | 0.996 | 0.889 | 0.944 |
| 8260 | 0.951 | 0.914 | 0.992 | 0.952 | 0.996 | 0.889 | 0.944 |
| 8270 | 0.947 | 0.909 | 0.992 | 0.952 | 0.996 | 0.889 | 0.944 |
| 8280 | 0.947 | 0.909 | 0.992 | 0.952 | 0.996 | 0.889 | 0.944 |
| 8290 | 0.947 | 0.909 | 0.992 | 0.952 | 0.996 | 0.889 | 0.944 |
| 8300 | 0.951 | 0.905 | 0.992 | 0.952 | 0.996 | 0.889 | 0.948 |
| 8310 | 0.947 | 0.909 | 0.992 | 0.952 | 0.996 | 0.889 | 0.948 |
| 8320 | 0.947 | 0.914 | 0.992 | 0.952 | 0.996 | 0.889 | 0.952 |
| 8330 | 0.947 | 0.914 | 0.992 | 0.952 | 0.996 | 0.889 | 0.948 |
| 8340 | 0.947 | 0.914 | 0.992 | 0.952 | 0.996 | 0.889 | 0.948 |
| 8350 | 0.947 | 0.914 | 0.992 | 0.952 | 0.996 | 0.889 | 0.944 |
| 8360 | 0.947 | 0.914 | 0.992 | 0.952 | 0.996 | 0.889 | 0.944 |
| 8370 | 0.947 | 0.914 | 0.992 | 0.952 | 0.996 | 0.889 | 0.944 |
| 8380 | 0.947 | 0.914 | 0.992 | 0.952 | 0.996 | 0.889 | 0.944 |
| 8390 | 0.947 | 0.914 | 0.992 | 0.952 | 0.996 | 0.889 | 0.944 |
| 8400 | 0.947 | 0.905 | 0.992 | 0.952 | 0.996 | 0.889 | 0.944 |
| 8410 | 0.947 | 0.905 | 0.992 | 0.952 | 0.996 | 0.889 | 0.952 |
| 8420 | 0.947 | 0.905 | 0.992 | 0.952 | 0.996 | 0.889 | 0.948 |
| 8430 | 0.947 | 0.901 | 0.992 | 0.952 | 0.996 | 0.889 | 0.952 |
| 8440 | 0.947 | 0.909 | 0.992 | 0.952 | 0.996 | 0.889 | 0.944 |
| 8450 | 0.947 | 0.909 | 0.992 | 0.952 | 0.996 | 0.889 | 0.940 |
| 8460 | 0.947 | 0.914 | 0.992 | 0.952 | 0.996 | 0.889 | 0.936 |
| 8470 | 0.947 | 0.905 | 0.992 | 0.952 | 0.996 | 0.889 | 0.944 |
| 8480 | 0.947 | 0.909 | 0.992 | 0.952 | 0.996 | 0.889 | 0.944 |
| 8490 | 0.947 | 0.905 | 0.992 | 0.952 | 0.996 | 0.889 | 0.952 |
| 8500 | 0.947 | 0.905 | 0.992 | 0.952 | 0.996 | 0.889 | 0.952 |
| 8510 | 0.951 | 0.909 | 0.992 | 0.952 | 0.996 | 0.889 | 0.948 |
| 8520 | 0.951 | 0.909 | 0.992 | 0.952 | 0.996 | 0.889 | 0.948 |
| 8530 | 0.951 | 0.905 | 0.992 | 0.952 | 0.996 | 0.889 | 0.952 |
| 8540 | 0.951 | 0.909 | 0.992 | 0.952 | 0.996 | 0.889 | 0.952 |
| 8550 | 0.951 | 0.905 | 0.992 | 0.952 | 0.996 | 0.889 | 0.952 |
| 8560 | 0.951 | 0.905 | 0.992 | 0.952 | 0.996 | 0.889 | 0.952 |
| 8570 | 0.947 | 0.901 | 0.992 | 0.952 | 0.996 | 0.889 | 0.952 |
| 8580 | 0.947 | 0.905 | 0.992 | 0.952 | 0.996 | 0.889 | 0.952 |
| 8590 | 0.947 | 0.901 | 0.992 | 0.952 | 0.996 | 0.889 | 0.956 |
| 8600 | 0.947 | 0.901 | 0.992 | 0.952 | 0.996 | 0.889 | 0.952 |
| 8610 | 0.947 | 0.901 | 0.992 | 0.952 | 0.996 | 0.889 | 0.952 |
| 8620 | 0.947 | 0.901 | 0.992 | 0.952 | 0.996 | 0.889 | 0.952 |
| 8630 | 0.947 | 0.901 | 0.992 | 0.952 | 0.996 | 0.889 | 0.952 |
| 8640 | 0.951 | 0.901 | 0.992 | 0.952 | 0.996 | 0.889 | 0.952 |
| 8650 | 0.951 | 0.897 | 0.992 | 0.952 | 0.996 | 0.889 | 0.952 |
| 8660 | 0.947 | 0.897 | 0.992 | 0.952 | 0.996 | 0.889 | 0.956 |
| 8670 | 0.951 | 0.897 | 0.992 | 0.952 | 0.996 | 0.889 | 0.952 |
| 8680 | 0.955 | 0.893 | 0.992 | 0.952 | 0.996 | 0.889 | 0.952 |
| 8690 | 0.955 | 0.893 | 0.992 | 0.952 | 0.996 | 0.889 | 0.952 |
| 8700 | 0.951 | 0.893 | 0.992 | 0.952 | 0.996 | 0.893 | 0.952 |
| 8710 | 0.955 | 0.889 | 0.992 | 0.952 | 0.996 | 0.893 | 0.956 |
| 8720 | 0.955 | 0.889 | 0.992 | 0.952 | 0.996 | 0.893 | 0.952 |
| 8730 | 0.955 | 0.889 | 0.992 | 0.952 | 0.996 | 0.893 | 0.952 |
| 8740 | 0.951 | 0.893 | 0.992 | 0.952 | 0.996 | 0.893 | 0.952 |
| 8750 | 0.955 | 0.893 | 0.992 | 0.952 | 0.996 | 0.893 | 0.952 |
| 8760 | 0.955 | 0.893 | 0.992 | 0.952 | 0.996 | 0.893 | 0.952 |
| 8770 | 0.955 | 0.893 | 0.992 | 0.952 | 0.996 | 0.893 | 0.948 |
| 8780 | 0.951 | 0.893 | 0.992 | 0.952 | 0.996 | 0.893 | 0.952 |
| 8790 | 0.951 | 0.893 | 0.992 | 0.952 | 0.996 | 0.893 | 0.952 |
| 8800 | 0.951 | 0.893 | 0.992 | 0.952 | 0.996 | 0.893 | 0.956 |
| 8810 | 0.951 | 0.897 | 0.992 | 0.952 | 0.996 | 0.889 | 0.960 |
| 8820 | 0.951 | 0.893 | 0.992 | 0.952 | 0.996 | 0.889 | 0.956 |
| 8830 | 0.951 | 0.897 | 0.992 | 0.952 | 0.996 | 0.889 | 0.956 |
| 8840 | 0.951 | 0.893 | 0.992 | 0.952 | 0.996 | 0.889 | 0.956 |
| 8850 | 0.951 | 0.893 | 0.992 | 0.952 | 0.996 | 0.889 | 0.956 |
| 8860 | 0.951 | 0.897 | 0.992 | 0.952 | 0.996 | 0.889 | 0.952 |
| 8870 | 0.951 | 0.897 | 0.992 | 0.952 | 0.996 | 0.889 | 0.948 |
| 8880 | 0.951 | 0.901 | 0.992 | 0.952 | 0.996 | 0.889 | 0.952 |
| 8890 | 0.951 | 0.901 | 0.992 | 0.952 | 0.996 | 0.880 | 0.952 |
| 8900 | 0.951 | 0.897 | 0.992 | 0.952 | 0.996 | 0.880 | 0.960 |
| 8910 | 0.951 | 0.897 | 0.992 | 0.952 | 0.996 | 0.880 | 0.960 |
| 8920 | 0.951 | 0.901 | 0.992 | 0.952 | 0.996 | 0.880 | 0.956 |
| 8930 | 0.955 | 0.901 | 0.992 | 0.952 | 0.996 | 0.880 | 0.956 |
| 8940 | 0.951 | 0.905 | 0.992 | 0.952 | 0.996 | 0.880 | 0.960 |
| 8950 | 0.951 | 0.905 | 0.992 | 0.952 | 0.996 | 0.880 | 0.952 |
| 8960 | 0.951 | 0.905 | 0.992 | 0.952 | 0.996 | 0.880 | 0.956 |
| 8970 | 0.951 | 0.905 | 0.992 | 0.952 | 0.996 | 0.880 | 0.952 |
| 8980 | 0.951 | 0.905 | 0.992 | 0.952 | 0.996 | 0.884 | 0.952 |
| 8990 | 0.955 | 0.901 | 0.992 | 0.952 | 0.996 | 0.876 | 0.952 |
| 9000 | 0.955 | 0.905 | 0.992 | 0.952 | 0.996 | 0.876 | 0.952 |
| 9010 | 0.955 | 0.901 | 0.992 | 0.952 | 0.996 | 0.876 | 0.952 |
| 9020 | 0.955 | 0.901 | 0.992 | 0.952 | 0.996 | 0.876 | 0.960 |
| 9030 | 0.955 | 0.901 | 0.992 | 0.952 | 0.996 | 0.876 | 0.956 |
| 9040 | 0.955 | 0.897 | 0.992 | 0.952 | 0.996 | 0.876 | 0.964 |
| 9050 | 0.955 | 0.897 | 0.992 | 0.952 | 0.996 | 0.876 | 0.964 |
| 9060 | 0.955 | 0.897 | 0.992 | 0.952 | 0.996 | 0.876 | 0.960 |
| 9070 | 0.955 | 0.897 | 0.992 | 0.952 | 0.996 | 0.876 | 0.960 |
| 9080 | 0.955 | 0.893 | 0.992 | 0.952 | 0.996 | 0.880 | 0.960 |
| 9090 | 0.955 | 0.893 | 0.992 | 0.952 | 0.996 | 0.880 | 0.960 |
| 9100 | 0.955 | 0.893 | 0.992 | 0.952 | 0.996 | 0.880 | 0.960 |
| 9110 | 0.955 | 0.893 | 0.992 | 0.952 | 0.996 | 0.880 | 0.960 |
| 9120 | 0.955 | 0.893 | 0.992 | 0.952 | 0.996 | 0.880 | 0.960 |
| 9130 | 0.955 | 0.893 | 0.992 | 0.952 | 0.996 | 0.880 | 0.960 |
| 9140 | 0.955 | 0.893 | 0.992 | 0.952 | 0.996 | 0.880 | 0.960 |
| 9150 | 0.955 | 0.893 | 0.992 | 0.952 | 0.996 | 0.880 | 0.960 |
| 9160 | 0.955 | 0.897 | 0.992 | 0.952 | 0.996 | 0.871 | 0.964 |
| 9170 | 0.955 | 0.897 | 0.992 | 0.952 | 0.996 | 0.871 | 0.964 |
| 9180 | 0.955 | 0.897 | 0.992 | 0.952 | 0.996 | 0.871 | 0.964 |
| 9190 | 0.955 | 0.897 | 0.992 | 0.952 | 1.000 | 0.876 | 0.960 |
| 9200 | 0.955 | 0.897 | 0.992 | 0.952 | 1.000 | 0.876 | 0.960 |
| 9210 | 0.955 | 0.901 | 0.992 | 0.952 | 1.000 | 0.871 | 0.960 |
| 9220 | 0.955 | 0.901 | 0.992 | 0.952 | 1.000 | 0.876 | 0.960 |
| 9230 | 0.955 | 0.897 | 0.992 | 0.952 | 1.000 | 0.876 | 0.960 |
| 9240 | 0.955 | 0.897 | 0.992 | 0.952 | 1.000 | 0.876 | 0.960 |
| 9250 | 0.955 | 0.897 | 0.992 | 0.952 | 1.000 | 0.876 | 0.960 |
| 9260 | 0.955 | 0.897 | 0.992 | 0.952 | 0.996 | 0.876 | 0.960 |
| 9270 | 0.955 | 0.897 | 0.992 | 0.952 | 0.996 | 0.876 | 0.960 |
| 9280 | 0.955 | 0.897 | 0.992 | 0.952 | 0.996 | 0.876 | 0.960 |
| 9290 | 0.955 | 0.897 | 0.992 | 0.952 | 0.996 | 0.876 | 0.960 |
| 9300 | 0.955 | 0.897 | 0.992 | 0.952 | 0.996 | 0.876 | 0.960 |
| 9310 | 0.959 | 0.893 | 0.992 | 0.952 | 0.996 | 0.876 | 0.960 |
| 9320 | 0.959 | 0.889 | 0.992 | 0.952 | 0.996 | 0.876 | 0.960 |
| 9330 | 0.959 | 0.889 | 0.992 | 0.952 | 0.996 | 0.876 | 0.960 |
| 9340 | 0.963 | 0.889 | 0.992 | 0.952 | 0.996 | 0.876 | 0.960 |
| 9350 | 0.963 | 0.889 | 0.992 | 0.952 | 0.996 | 0.876 | 0.960 |
| 9360 | 0.963 | 0.889 | 0.992 | 0.952 | 0.996 | 0.876 | 0.960 |
| 9370 | 0.963 | 0.889 | 0.992 | 0.952 | 1.000 | 0.876 | 0.956 |
| 9380 | 0.963 | 0.889 | 0.992 | 0.952 | 1.000 | 0.876 | 0.956 |
| 9390 | 0.963 | 0.889 | 0.992 | 0.952 | 0.996 | 0.876 | 0.960 |
| 9400 | 0.963 | 0.889 | 0.992 | 0.952 | 1.000 | 0.876 | 0.956 |
| 9410 | 0.963 | 0.889 | 0.992 | 0.952 | 1.000 | 0.876 | 0.956 |
| 9420 | 0.963 | 0.889 | 0.992 | 0.952 | 1.000 | 0.876 | 0.956 |
| 9430 | 0.963 | 0.889 | 0.992 | 0.952 | 0.996 | 0.876 | 0.960 |
| 9440 | 0.963 | 0.889 | 0.992 | 0.952 | 0.996 | 0.876 | 0.960 |
| 9450 | 0.963 | 0.889 | 0.992 | 0.952 | 0.996 | 0.876 | 0.960 |
| 9460 | 0.963 | 0.889 | 0.992 | 0.948 | 1.000 | 0.876 | 0.956 |
| 9470 | 0.963 | 0.889 | 0.992 | 0.948 | 1.000 | 0.871 | 0.956 |
| 9480 | 0.963 | 0.893 | 0.992 | 0.948 | 1.000 | 0.876 | 0.956 |
| 9490 | 0.963 | 0.893 | 0.992 | 0.948 | 0.996 | 0.876 | 0.960 |
| 9500 | 0.963 | 0.893 | 0.992 | 0.948 | 1.000 | 0.871 | 0.956 |
| 9510 | 0.963 | 0.889 | 0.992 | 0.948 | 0.996 | 0.871 | 0.960 |
| 9520 | 0.963 | 0.889 | 0.992 | 0.948 | 0.996 | 0.871 | 0.960 |
| 9530 | 0.963 | 0.889 | 0.992 | 0.948 | 0.996 | 0.876 | 0.960 |
| 9540 | 0.963 | 0.897 | 0.992 | 0.948 | 1.000 | 0.876 | 0.952 |
| 9550 | 0.963 | 0.889 | 0.992 | 0.948 | 0.996 | 0.876 | 0.960 |
| 9560 | 0.963 | 0.897 | 0.992 | 0.948 | 0.996 | 0.876 | 0.956 |
| 9570 | 0.963 | 0.901 | 0.992 | 0.948 | 0.996 | 0.871 | 0.956 |
| 9580 | 0.963 | 0.897 | 0.992 | 0.948 | 0.996 | 0.876 | 0.956 |
| 9590 | 0.963 | 0.897 | 0.992 | 0.948 | 0.996 | 0.876 | 0.956 |
| 9600 | 0.963 | 0.897 | 0.992 | 0.948 | 0.996 | 0.876 | 0.956 |
| 9610 | 0.963 | 0.893 | 0.992 | 0.948 | 0.996 | 0.876 | 0.960 |
| 9620 | 0.959 | 0.901 | 0.992 | 0.948 | 0.996 | 0.876 | 0.956 |
| 9630 | 0.963 | 0.901 | 0.992 | 0.948 | 0.996 | 0.876 | 0.956 |
| 9640 | 0.959 | 0.901 | 0.992 | 0.948 | 0.996 | 0.876 | 0.956 |
| 9650 | 0.959 | 0.901 | 0.992 | 0.948 | 0.996 | 0.876 | 0.956 |
| 9660 | 0.959 | 0.901 | 0.992 | 0.948 | 0.996 | 0.876 | 0.952 |
| 9670 | 0.959 | 0.901 | 0.992 | 0.948 | 0.996 | 0.876 | 0.956 |
| 9680 | 0.959 | 0.901 | 0.992 | 0.948 | 0.996 | 0.876 | 0.952 |
| 9690 | 0.959 | 0.901 | 0.992 | 0.948 | 0.996 | 0.876 | 0.952 |
| 9700 | 0.959 | 0.901 | 0.992 | 0.948 | 0.996 | 0.867 | 0.952 |
| 9710 | 0.959 | 0.897 | 0.992 | 0.948 | 0.996 | 0.867 | 0.952 |
| 9720 | 0.959 | 0.901 | 0.992 | 0.948 | 0.996 | 0.871 | 0.952 |
| 9730 | 0.959 | 0.901 | 0.992 | 0.948 | 0.996 | 0.867 | 0.952 |
| 9740 | 0.959 | 0.897 | 0.992 | 0.948 | 0.996 | 0.871 | 0.952 |
| 9750 | 0.959 | 0.901 | 0.992 | 0.948 | 0.996 | 0.871 | 0.952 |
| 9760 | 0.959 | 0.905 | 0.992 | 0.948 | 0.996 | 0.876 | 0.952 |
| 9770 | 0.959 | 0.905 | 0.992 | 0.948 | 0.996 | 0.871 | 0.952 |
| 9780 | 0.959 | 0.905 | 0.992 | 0.948 | 0.996 | 0.876 | 0.952 |
| 9790 | 0.955 | 0.905 | 0.992 | 0.948 | 0.996 | 0.876 | 0.952 |
| 9800 | 0.959 | 0.905 | 0.992 | 0.948 | 0.996 | 0.876 | 0.952 |
| 9810 | 0.959 | 0.901 | 0.992 | 0.948 | 0.996 | 0.871 | 0.952 |
| 9820 | 0.959 | 0.901 | 0.992 | 0.948 | 0.996 | 0.871 | 0.952 |
| 9830 | 0.959 | 0.901 | 0.992 | 0.948 | 0.996 | 0.871 | 0.952 |
| 9840 | 0.959 | 0.901 | 0.992 | 0.948 | 0.996 | 0.871 | 0.952 |
| 9850 | 0.959 | 0.901 | 0.992 | 0.948 | 0.996 | 0.871 | 0.952 |
| 9860 | 0.959 | 0.897 | 0.992 | 0.948 | 0.996 | 0.871 | 0.952 |
| 9870 | 0.959 | 0.901 | 0.992 | 0.948 | 0.996 | 0.871 | 0.952 |
| 9880 | 0.959 | 0.897 | 0.992 | 0.948 | 0.996 | 0.867 | 0.948 |
| 9890 | 0.959 | 0.901 | 0.992 | 0.948 | 0.996 | 0.867 | 0.952 |
| 9900 | 0.959 | 0.901 | 0.992 | 0.948 | 0.996 | 0.867 | 0.948 |
| 9910 | 0.959 | 0.901 | 0.992 | 0.948 | 0.996 | 0.867 | 0.944 |
| 9920 | 0.959 | 0.897 | 0.992 | 0.948 | 0.996 | 0.867 | 0.944 |
| 9930 | 0.955 | 0.897 | 0.992 | 0.948 | 0.996 | 0.867 | 0.948 |
| 9940 | 0.955 | 0.889 | 0.992 | 0.948 | 0.996 | 0.867 | 0.956 |
| 9950 | 0.955 | 0.893 | 0.992 | 0.948 | 0.996 | 0.867 | 0.948 |
| 9960 | 0.955 | 0.893 | 0.992 | 0.948 | 0.996 | 0.867 | 0.948 |
| 9970 | 0.955 | 0.893 | 0.992 | 0.948 | 0.996 | 0.867 | 0.952 |
| 9980 | 0.955 | 0.893 | 0.992 | 0.948 | 0.996 | 0.867 | 0.952 |
| 9990 | 0.955 | 0.893 | 0.992 | 0.948 | 0.996 | 0.867 | 0.952 |
| 10000 | 0.955 | 0.893 | 0.992 | 0.948 | 0.996 | 0.867 | 0.952 |
| 10010 | 0.955 | 0.893 | 0.992 | 0.948 | 0.996 | 0.867 | 0.952 |
| 10020 | 0.955 | 0.893 | 0.992 | 0.948 | 0.996 | 0.867 | 0.952 |
| 10030 | 0.955 | 0.889 | 0.992 | 0.948 | 0.996 | 0.867 | 0.956 |
| 10040 | 0.955 | 0.889 | 0.992 | 0.948 | 0.996 | 0.867 | 0.956 |
| 10050 | 0.955 | 0.889 | 0.992 | 0.948 | 0.996 | 0.867 | 0.956 |
| 10060 | 0.951 | 0.893 | 0.992 | 0.948 | 0.996 | 0.867 | 0.956 |
| 10070 | 0.955 | 0.893 | 0.992 | 0.948 | 0.996 | 0.867 | 0.956 |
| 10080 | 0.951 | 0.897 | 0.992 | 0.948 | 0.996 | 0.867 | 0.952 |
| 10090 | 0.951 | 0.897 | 0.992 | 0.948 | 0.996 | 0.867 | 0.952 |
| 10100 | 0.951 | 0.897 | 0.992 | 0.948 | 0.996 | 0.867 | 0.952 |
| 10110 | 0.951 | 0.897 | 0.992 | 0.948 | 0.996 | 0.867 | 0.952 |
| 10120 | 0.951 | 0.897 | 0.992 | 0.948 | 0.996 | 0.867 | 0.952 |
| 10130 | 0.955 | 0.893 | 0.992 | 0.948 | 0.996 | 0.867 | 0.952 |
| 10140 | 0.951 | 0.893 | 0.992 | 0.948 | 0.996 | 0.871 | 0.952 |
| 10150 | 0.955 | 0.893 | 0.992 | 0.948 | 0.996 | 0.867 | 0.948 |
| 10160 | 0.955 | 0.893 | 0.992 | 0.948 | 0.996 | 0.867 | 0.952 |
| 10170 | 0.955 | 0.893 | 0.992 | 0.948 | 0.996 | 0.867 | 0.948 |
| 10180 | 0.955 | 0.893 | 0.992 | 0.948 | 0.996 | 0.867 | 0.952 |
| 10190 | 0.951 | 0.897 | 0.992 | 0.948 | 0.996 | 0.867 | 0.952 |
| 10200 | 0.951 | 0.897 | 0.992 | 0.948 | 0.996 | 0.867 | 0.952 |
| 10210 | 0.951 | 0.901 | 0.992 | 0.948 | 0.996 | 0.867 | 0.948 |
| 10220 | 0.951 | 0.897 | 0.992 | 0.948 | 1.000 | 0.867 | 0.944 |
| 10230 | 0.951 | 0.893 | 0.992 | 0.948 | 1.000 | 0.867 | 0.952 |
| 10240 | 0.951 | 0.893 | 0.992 | 0.948 | 1.000 | 0.867 | 0.948 |
| 10250 | 0.947 | 0.893 | 0.992 | 0.948 | 1.000 | 0.871 | 0.948 |
| 10260 | 0.947 | 0.893 | 0.992 | 0.948 | 1.000 | 0.871 | 0.948 |
| 10270 | 0.947 | 0.897 | 0.992 | 0.948 | 1.000 | 0.871 | 0.940 |
| 10280 | 0.947 | 0.897 | 0.992 | 0.948 | 1.000 | 0.871 | 0.944 |
| 10290 | 0.947 | 0.897 | 0.992 | 0.948 | 0.996 | 0.871 | 0.944 |
| 10300 | 0.947 | 0.893 | 0.992 | 0.948 | 0.996 | 0.871 | 0.948 |
| 10310 | 0.947 | 0.897 | 0.992 | 0.948 | 0.996 | 0.871 | 0.944 |
| 10320 | 0.947 | 0.897 | 0.992 | 0.948 | 0.996 | 0.871 | 0.948 |
| 10330 | 0.947 | 0.897 | 0.992 | 0.948 | 0.996 | 0.871 | 0.948 |
| 10340 | 0.947 | 0.897 | 0.992 | 0.948 | 0.996 | 0.871 | 0.948 |
| 10350 | 0.947 | 0.893 | 0.992 | 0.948 | 1.000 | 0.871 | 0.952 |
| 10360 | 0.947 | 0.893 | 0.992 | 0.948 | 0.996 | 0.871 | 0.952 |
| 10370 | 0.947 | 0.897 | 0.992 | 0.948 | 1.000 | 0.871 | 0.944 |
| 10380 | 0.947 | 0.897 | 0.992 | 0.948 | 1.000 | 0.871 | 0.944 |
| 10390 | 0.951 | 0.897 | 0.992 | 0.948 | 1.000 | 0.867 | 0.940 |
| 10400 | 0.951 | 0.897 | 0.992 | 0.948 | 0.996 | 0.867 | 0.944 |
| 10410 | 0.951 | 0.893 | 0.992 | 0.948 | 0.996 | 0.867 | 0.944 |
| 10420 | 0.951 | 0.893 | 0.992 | 0.948 | 1.000 | 0.867 | 0.940 |
| 10430 | 0.951 | 0.893 | 0.992 | 0.948 | 1.000 | 0.867 | 0.944 |
| 10440 | 0.951 | 0.893 | 0.992 | 0.948 | 0.996 | 0.867 | 0.944 |
| 10450 | 0.955 | 0.889 | 0.992 | 0.948 | 1.000 | 0.867 | 0.944 |
| 10460 | 0.951 | 0.893 | 0.992 | 0.948 | 1.000 | 0.867 | 0.944 |
| 10470 | 0.951 | 0.893 | 0.992 | 0.948 | 1.000 | 0.867 | 0.944 |
| 10480 | 0.951 | 0.893 | 0.992 | 0.948 | 1.000 | 0.867 | 0.944 |
| 10490 | 0.951 | 0.893 | 0.992 | 0.948 | 1.000 | 0.867 | 0.944 |
| 10500 | 0.951 | 0.893 | 0.992 | 0.948 | 1.000 | 0.867 | 0.944 |
| 10510 | 0.955 | 0.889 | 0.992 | 0.948 | 1.000 | 0.867 | 0.944 |
| 10520 | 0.955 | 0.889 | 0.992 | 0.948 | 0.996 | 0.867 | 0.948 |
| 10530 | 0.955 | 0.889 | 0.992 | 0.948 | 0.996 | 0.867 | 0.948 |
| 10540 | 0.951 | 0.893 | 0.992 | 0.948 | 0.996 | 0.867 | 0.948 |
| 10550 | 0.951 | 0.893 | 0.992 | 0.948 | 0.996 | 0.867 | 0.948 |
| 10560 | 0.951 | 0.893 | 0.992 | 0.948 | 0.996 | 0.867 | 0.952 |
| 10570 | 0.951 | 0.893 | 0.992 | 0.948 | 0.996 | 0.867 | 0.956 |
| 10580 | 0.951 | 0.893 | 0.992 | 0.948 | 0.996 | 0.867 | 0.952 |
| 10590 | 0.951 | 0.893 | 0.992 | 0.948 | 1.000 | 0.867 | 0.948 |
| 10600 | 0.951 | 0.893 | 0.992 | 0.948 | 0.996 | 0.867 | 0.952 |
| 10610 | 0.951 | 0.893 | 0.992 | 0.948 | 1.000 | 0.867 | 0.948 |
| 10620 | 0.951 | 0.893 | 0.992 | 0.948 | 1.000 | 0.867 | 0.948 |
| 10630 | 0.951 | 0.893 | 0.992 | 0.948 | 1.000 | 0.867 | 0.944 |
| 10640 | 0.951 | 0.893 | 0.992 | 0.948 | 1.000 | 0.867 | 0.948 |
| 10650 | 0.951 | 0.893 | 0.992 | 0.948 | 1.000 | 0.867 | 0.948 |
| 10660 | 0.951 | 0.893 | 0.992 | 0.948 | 1.000 | 0.867 | 0.944 |
| 10670 | 0.951 | 0.893 | 0.992 | 0.948 | 1.000 | 0.867 | 0.944 |
| 10680 | 0.955 | 0.889 | 0.992 | 0.948 | 1.000 | 0.867 | 0.948 |
| 10690 | 0.955 | 0.889 | 0.992 | 0.948 | 1.000 | 0.867 | 0.948 |
| 10700 | 0.951 | 0.893 | 0.992 | 0.948 | 1.000 | 0.867 | 0.944 |
| 10710 | 0.951 | 0.893 | 0.992 | 0.948 | 1.000 | 0.867 | 0.940 |
| 10720 | 0.951 | 0.893 | 0.992 | 0.948 | 1.000 | 0.867 | 0.940 |
| 10730 | 0.955 | 0.889 | 0.992 | 0.948 | 1.000 | 0.867 | 0.940 |
| 10740 | 0.955 | 0.889 | 0.992 | 0.943 | 1.000 | 0.867 | 0.944 |
| 10750 | 0.955 | 0.889 | 0.992 | 0.943 | 1.000 | 0.867 | 0.944 |
| 10760 | 0.955 | 0.889 | 0.992 | 0.943 | 1.000 | 0.867 | 0.940 |
| 10770 | 0.955 | 0.889 | 0.992 | 0.948 | 1.000 | 0.867 | 0.940 |
| 10780 | 0.955 | 0.889 | 0.992 | 0.943 | 1.000 | 0.867 | 0.940 |
| 10790 | 0.955 | 0.889 | 0.992 | 0.943 | 1.000 | 0.867 | 0.940 |
| 10800 | 0.955 | 0.889 | 0.992 | 0.943 | 1.000 | 0.867 | 0.940 |
| 10810 | 0.951 | 0.893 | 0.992 | 0.943 | 1.000 | 0.867 | 0.944 |
| 10820 | 0.951 | 0.893 | 0.992 | 0.943 | 1.000 | 0.867 | 0.940 |
| 10830 | 0.951 | 0.893 | 0.992 | 0.943 | 1.000 | 0.867 | 0.940 |
| 10840 | 0.951 | 0.893 | 0.992 | 0.943 | 1.000 | 0.867 | 0.948 |
| 10850 | 0.951 | 0.893 | 0.992 | 0.943 | 1.000 | 0.867 | 0.944 |
| 10860 | 0.951 | 0.893 | 0.992 | 0.943 | 1.000 | 0.867 | 0.940 |
| 10870 | 0.951 | 0.893 | 0.992 | 0.943 | 1.000 | 0.867 | 0.944 |
| 10880 | 0.951 | 0.893 | 0.992 | 0.943 | 0.996 | 0.867 | 0.952 |
| 10890 | 0.951 | 0.893 | 0.992 | 0.943 | 0.996 | 0.867 | 0.948 |
| 10900 | 0.951 | 0.893 | 0.992 | 0.943 | 0.996 | 0.867 | 0.948 |
| 10910 | 0.951 | 0.893 | 0.992 | 0.943 | 0.996 | 0.867 | 0.948 |
| 10920 | 0.951 | 0.893 | 0.992 | 0.943 | 0.996 | 0.867 | 0.948 |
| 10930 | 0.951 | 0.893 | 0.992 | 0.943 | 0.996 | 0.867 | 0.948 |
| 10940 | 0.951 | 0.893 | 0.992 | 0.943 | 0.996 | 0.867 | 0.948 |
| 10950 | 0.951 | 0.893 | 0.992 | 0.943 | 0.996 | 0.867 | 0.948 |
| 10960 | 0.951 | 0.893 | 0.992 | 0.948 | 0.996 | 0.867 | 0.944 |
| 10970 | 0.951 | 0.893 | 0.992 | 0.948 | 0.996 | 0.867 | 0.944 |
| 10980 | 0.951 | 0.893 | 0.992 | 0.948 | 0.996 | 0.867 | 0.944 |
| 10990 | 0.951 | 0.893 | 0.992 | 0.948 | 0.996 | 0.867 | 0.944 |
| 11000 | 0.951 | 0.893 | 0.992 | 0.943 | 0.996 | 0.867 | 0.944 |
| 11010 | 0.951 | 0.893 | 0.992 | 0.948 | 0.996 | 0.867 | 0.944 |
| 11020 | 0.951 | 0.893 | 0.992 | 0.948 | 1.000 | 0.867 | 0.940 |
| 11030 | 0.951 | 0.893 | 0.992 | 0.948 | 1.000 | 0.867 | 0.940 |
| 11040 | 0.951 | 0.893 | 0.992 | 0.943 | 1.000 | 0.867 | 0.940 |
| 11050 | 0.951 | 0.893 | 0.992 | 0.943 | 1.000 | 0.867 | 0.936 |
| 11060 | 0.951 | 0.893 | 0.992 | 0.943 | 1.000 | 0.867 | 0.936 |
| 11070 | 0.951 | 0.893 | 0.992 | 0.943 | 1.000 | 0.867 | 0.936 |
| 11080 | 0.951 | 0.893 | 0.992 | 0.943 | 1.000 | 0.867 | 0.936 |
| 11090 | 0.955 | 0.889 | 0.992 | 0.943 | 1.000 | 0.867 | 0.936 |
| 11100 | 0.955 | 0.889 | 0.992 | 0.943 | 1.000 | 0.867 | 0.936 |
| 11110 | 0.955 | 0.889 | 0.992 | 0.943 | 1.000 | 0.867 | 0.936 |
| 11120 | 0.955 | 0.889 | 0.992 | 0.943 | 1.000 | 0.867 | 0.936 |
| 11130 | 0.955 | 0.889 | 0.992 | 0.943 | 1.000 | 0.867 | 0.936 |
| 11140 | 0.951 | 0.893 | 0.992 | 0.943 | 1.000 | 0.867 | 0.936 |
| 11150 | 0.951 | 0.893 | 0.992 | 0.943 | 1.000 | 0.867 | 0.936 |
| 11160 | 0.951 | 0.893 | 0.992 | 0.943 | 1.000 | 0.867 | 0.936 |
| 11170 | 0.951 | 0.893 | 0.992 | 0.943 | 1.000 | 0.867 | 0.936 |
| 11180 | 0.951 | 0.893 | 0.992 | 0.943 | 1.000 | 0.867 | 0.936 |
| 11190 | 0.951 | 0.893 | 0.992 | 0.943 | 1.000 | 0.867 | 0.936 |
| 11200 | 0.955 | 0.889 | 0.992 | 0.943 | 1.000 | 0.867 | 0.932 |
| 11210 | 0.955 | 0.889 | 0.992 | 0.943 | 1.000 | 0.867 | 0.936 |
| 11220 | 0.955 | 0.885 | 0.992 | 0.943 | 1.000 | 0.867 | 0.936 |
| 11230 | 0.955 | 0.885 | 0.992 | 0.943 | 1.000 | 0.867 | 0.936 |
| 11240 | 0.955 | 0.885 | 0.992 | 0.943 | 1.000 | 0.867 | 0.936 |
| 11250 | 0.951 | 0.885 | 0.992 | 0.943 | 1.000 | 0.867 | 0.936 |
| 11260 | 0.955 | 0.885 | 0.992 | 0.943 | 1.000 | 0.867 | 0.936 |
| 11270 | 0.947 | 0.889 | 0.992 | 0.943 | 1.000 | 0.867 | 0.936 |
| 11280 | 0.947 | 0.889 | 0.992 | 0.948 | 1.000 | 0.867 | 0.932 |
| 11290 | 0.947 | 0.889 | 0.992 | 0.943 | 1.000 | 0.867 | 0.932 |
| 11300 | 0.947 | 0.889 | 0.992 | 0.943 | 1.000 | 0.867 | 0.936 |
| 11310 | 0.947 | 0.889 | 0.992 | 0.943 | 1.000 | 0.867 | 0.936 |
| 11320 | 0.947 | 0.889 | 0.992 | 0.943 | 1.000 | 0.867 | 0.936 |
| 11330 | 0.947 | 0.889 | 0.992 | 0.943 | 1.000 | 0.867 | 0.936 |
| 11340 | 0.947 | 0.889 | 0.992 | 0.943 | 1.000 | 0.867 | 0.936 |
| 11350 | 0.947 | 0.889 | 0.992 | 0.943 | 1.000 | 0.867 | 0.932 |
| 11360 | 0.947 | 0.889 | 0.992 | 0.943 | 1.000 | 0.867 | 0.932 |
| 11370 | 0.947 | 0.889 | 0.992 | 0.943 | 1.000 | 0.867 | 0.932 |
| 11380 | 0.947 | 0.889 | 0.992 | 0.943 | 1.000 | 0.867 | 0.932 |
| 11390 | 0.947 | 0.889 | 0.992 | 0.943 | 1.000 | 0.867 | 0.928 |
| 11400 | 0.947 | 0.889 | 0.992 | 0.943 | 1.000 | 0.867 | 0.936 |
| 11410 | 0.947 | 0.889 | 0.992 | 0.943 | 1.000 | 0.867 | 0.928 |
| 11420 | 0.955 | 0.881 | 0.992 | 0.943 | 1.000 | 0.867 | 0.928 |
| 11430 | 0.955 | 0.881 | 0.992 | 0.943 | 1.000 | 0.867 | 0.928 |
| 11440 | 0.955 | 0.881 | 0.992 | 0.943 | 1.000 | 0.867 | 0.924 |
| 11450 | 0.955 | 0.881 | 0.992 | 0.943 | 1.000 | 0.867 | 0.924 |
| 11460 | 0.955 | 0.881 | 0.992 | 0.943 | 1.000 | 0.867 | 0.924 |
| 11470 | 0.955 | 0.881 | 0.992 | 0.943 | 1.000 | 0.867 | 0.928 |
| 11480 | 0.955 | 0.881 | 0.992 | 0.943 | 1.000 | 0.867 | 0.928 |
| 11490 | 0.955 | 0.877 | 0.992 | 0.943 | 1.000 | 0.867 | 0.928 |
| 11500 | 0.955 | 0.877 | 0.992 | 0.943 | 1.000 | 0.867 | 0.928 |
| 11510 | 0.955 | 0.877 | 0.992 | 0.943 | 1.000 | 0.867 | 0.924 |
| 11520 | 0.955 | 0.877 | 0.992 | 0.943 | 1.000 | 0.867 | 0.924 |
| 11530 | 0.955 | 0.877 | 0.992 | 0.943 | 1.000 | 0.867 | 0.928 |
| 11540 | 0.955 | 0.877 | 0.992 | 0.943 | 1.000 | 0.867 | 0.924 |
| 11550 | 0.955 | 0.881 | 0.992 | 0.943 | 1.000 | 0.867 | 0.928 |
| 11560 | 0.955 | 0.877 | 0.992 | 0.943 | 1.000 | 0.867 | 0.928 |
| 11570 | 0.955 | 0.877 | 0.992 | 0.943 | 1.000 | 0.867 | 0.924 |
| 11580 | 0.955 | 0.877 | 0.992 | 0.943 | 1.000 | 0.867 | 0.928 |
| 11590 | 0.955 | 0.877 | 0.992 | 0.943 | 1.000 | 0.867 | 0.928 |
| 11600 | 0.955 | 0.877 | 0.992 | 0.943 | 1.000 | 0.867 | 0.928 |
| 11610 | 0.955 | 0.872 | 0.992 | 0.943 | 1.000 | 0.867 | 0.928 |
| 11620 | 0.955 | 0.872 | 0.992 | 0.943 | 1.000 | 0.867 | 0.924 |
| 11630 | 0.955 | 0.877 | 0.992 | 0.943 | 1.000 | 0.867 | 0.924 |
| 11640 | 0.955 | 0.872 | 0.992 | 0.943 | 1.000 | 0.867 | 0.924 |
| 11650 | 0.955 | 0.872 | 0.992 | 0.943 | 1.000 | 0.867 | 0.924 |
| 11660 | 0.955 | 0.877 | 0.992 | 0.943 | 1.000 | 0.867 | 0.928 |
| 11670 | 0.955 | 0.877 | 0.992 | 0.943 | 1.000 | 0.867 | 0.928 |
| 11680 | 0.955 | 0.877 | 0.992 | 0.943 | 1.000 | 0.867 | 0.928 |
| 11690 | 0.955 | 0.877 | 0.992 | 0.943 | 1.000 | 0.867 | 0.932 |
| 11700 | 0.955 | 0.872 | 0.992 | 0.943 | 1.000 | 0.867 | 0.928 |
| 11710 | 0.955 | 0.872 | 0.992 | 0.943 | 1.000 | 0.867 | 0.928 |
| 11720 | 0.955 | 0.872 | 0.992 | 0.943 | 1.000 | 0.867 | 0.932 |
| 11730 | 0.955 | 0.872 | 0.992 | 0.943 | 1.000 | 0.867 | 0.932 |
| 11740 | 0.955 | 0.877 | 0.992 | 0.943 | 1.000 | 0.867 | 0.936 |
| 11750 | 0.955 | 0.877 | 0.992 | 0.943 | 1.000 | 0.862 | 0.936 |
| 11760 | 0.955 | 0.881 | 0.992 | 0.943 | 1.000 | 0.862 | 0.936 |
| 11770 | 0.955 | 0.881 | 0.992 | 0.943 | 1.000 | 0.862 | 0.932 |
| 11780 | 0.955 | 0.877 | 0.992 | 0.943 | 1.000 | 0.862 | 0.932 |
| 11790 | 0.955 | 0.877 | 0.992 | 0.943 | 1.000 | 0.862 | 0.932 |
| 11800 | 0.955 | 0.877 | 0.992 | 0.943 | 1.000 | 0.862 | 0.936 |
| 11810 | 0.955 | 0.877 | 0.992 | 0.943 | 1.000 | 0.862 | 0.936 |
| 11820 | 0.955 | 0.872 | 0.992 | 0.943 | 1.000 | 0.862 | 0.932 |
| 11830 | 0.955 | 0.877 | 0.992 | 0.943 | 1.000 | 0.862 | 0.928 |
| 11840 | 0.955 | 0.877 | 0.992 | 0.943 | 1.000 | 0.867 | 0.932 |
| 11850 | 0.955 | 0.877 | 0.992 | 0.943 | 1.000 | 0.862 | 0.928 |
| 11860 | 0.955 | 0.877 | 0.992 | 0.943 | 1.000 | 0.862 | 0.932 |
| 11870 | 0.955 | 0.877 | 0.992 | 0.943 | 1.000 | 0.862 | 0.928 |
| 11880 | 0.955 | 0.877 | 0.992 | 0.943 | 1.000 | 0.862 | 0.928 |
| 11890 | 0.955 | 0.877 | 0.992 | 0.943 | 1.000 | 0.862 | 0.928 |
| 11900 | 0.955 | 0.877 | 0.992 | 0.943 | 1.000 | 0.862 | 0.928 |
| 11910 | 0.955 | 0.877 | 0.992 | 0.943 | 1.000 | 0.862 | 0.928 |
| 11920 | 0.955 | 0.877 | 0.992 | 0.943 | 1.000 | 0.862 | 0.932 |
| 11930 | 0.955 | 0.877 | 0.992 | 0.943 | 1.000 | 0.862 | 0.932 |
| 11940 | 0.955 | 0.877 | 0.992 | 0.943 | 1.000 | 0.862 | 0.932 |
| 11950 | 0.955 | 0.877 | 0.992 | 0.943 | 1.000 | 0.862 | 0.932 |
| 11960 | 0.955 | 0.877 | 0.992 | 0.943 | 1.000 | 0.862 | 0.932 |
| 11970 | 0.955 | 0.877 | 0.992 | 0.943 | 1.000 | 0.862 | 0.928 |
| 11980 | 0.955 | 0.872 | 0.992 | 0.943 | 1.000 | 0.862 | 0.928 |
| 11990 | 0.955 | 0.872 | 0.992 | 0.943 | 1.000 | 0.862 | 0.928 |
| 12000 | 0.955 | 0.872 | 0.992 | 0.943 | 1.000 | 0.862 | 0.928 |
| 12010 | 0.951 | 0.877 | 0.992 | 0.943 | 1.000 | 0.862 | 0.928 |
| 12020 | 0.951 | 0.877 | 0.992 | 0.943 | 1.000 | 0.862 | 0.928 |
| 12030 | 0.951 | 0.877 | 0.992 | 0.943 | 1.000 | 0.862 | 0.928 |
| 12040 | 0.951 | 0.877 | 0.992 | 0.943 | 1.000 | 0.862 | 0.928 |
| 12050 | 0.951 | 0.877 | 0.992 | 0.943 | 1.000 | 0.862 | 0.928 |
| 12060 | 0.951 | 0.877 | 0.992 | 0.943 | 1.000 | 0.862 | 0.928 |
| 12070 | 0.947 | 0.877 | 0.992 | 0.943 | 1.000 | 0.862 | 0.928 |
| 12080 | 0.947 | 0.877 | 0.992 | 0.943 | 1.000 | 0.862 | 0.924 |
| 12090 | 0.951 | 0.872 | 0.992 | 0.943 | 1.000 | 0.862 | 0.924 |
| 12100 | 0.951 | 0.872 | 0.992 | 0.943 | 1.000 | 0.862 | 0.928 |
| 12110 | 0.955 | 0.872 | 0.992 | 0.943 | 1.000 | 0.862 | 0.924 |
| 12120 | 0.951 | 0.872 | 0.992 | 0.943 | 1.000 | 0.862 | 0.924 |
| 12130 | 0.951 | 0.872 | 0.992 | 0.943 | 1.000 | 0.862 | 0.928 |
| 12140 | 0.955 | 0.872 | 0.992 | 0.943 | 1.000 | 0.862 | 0.928 |
| 12150 | 0.951 | 0.872 | 0.992 | 0.943 | 1.000 | 0.862 | 0.928 |
| 12160 | 0.951 | 0.872 | 0.992 | 0.943 | 1.000 | 0.862 | 0.928 |
| 12170 | 0.951 | 0.872 | 0.992 | 0.943 | 1.000 | 0.862 | 0.928 |
| 12180 | 0.951 | 0.872 | 0.992 | 0.943 | 1.000 | 0.862 | 0.928 |
| 12190 | 0.951 | 0.872 | 0.992 | 0.943 | 1.000 | 0.862 | 0.928 |
| 12200 | 0.955 | 0.872 | 0.992 | 0.943 | 1.000 | 0.862 | 0.928 |
| 12210 | 0.955 | 0.872 | 0.992 | 0.943 | 1.000 | 0.862 | 0.928 |
| 12220 | 0.955 | 0.872 | 0.992 | 0.943 | 1.000 | 0.862 | 0.928 |
| 12230 | 0.955 | 0.872 | 0.992 | 0.943 | 1.000 | 0.862 | 0.928 |
| 12240 | 0.955 | 0.872 | 0.992 | 0.943 | 1.000 | 0.862 | 0.928 |
| 12250 | 0.955 | 0.872 | 0.992 | 0.943 | 1.000 | 0.862 | 0.928 |
| 12260 | 0.955 | 0.872 | 0.992 | 0.943 | 1.000 | 0.862 | 0.928 |
| 12270 | 0.955 | 0.872 | 0.992 | 0.943 | 1.000 | 0.862 | 0.928 |
| 12280 | 0.955 | 0.872 | 0.992 | 0.943 | 1.000 | 0.862 | 0.928 |
| 12290 | 0.955 | 0.872 | 0.992 | 0.943 | 1.000 | 0.862 | 0.928 |
| 12300 | 0.955 | 0.872 | 0.992 | 0.943 | 1.000 | 0.862 | 0.928 |
| 12310 | 0.955 | 0.872 | 0.992 | 0.943 | 1.000 | 0.862 | 0.928 |
| 12320 | 0.955 | 0.872 | 0.992 | 0.943 | 1.000 | 0.862 | 0.928 |
| 12330 | 0.955 | 0.872 | 0.992 | 0.943 | 1.000 | 0.862 | 0.928 |
| 12340 | 0.955 | 0.872 | 0.992 | 0.943 | 1.000 | 0.862 | 0.924 |
| 12350 | 0.955 | 0.872 | 0.992 | 0.943 | 1.000 | 0.862 | 0.928 |
| 12360 | 0.955 | 0.872 | 0.992 | 0.943 | 1.000 | 0.862 | 0.928 |
| 12370 | 0.955 | 0.872 | 0.992 | 0.943 | 1.000 | 0.862 | 0.928 |
| 12380 | 0.955 | 0.872 | 0.992 | 0.943 | 1.000 | 0.862 | 0.928 |
| 12390 | 0.955 | 0.872 | 0.992 | 0.943 | 1.000 | 0.862 | 0.928 |
| 12400 | 0.955 | 0.872 | 0.992 | 0.943 | 1.000 | 0.862 | 0.928 |
| 12410 | 0.955 | 0.872 | 0.992 | 0.943 | 1.000 | 0.862 | 0.928 |
| 12420 | 0.955 | 0.872 | 0.992 | 0.943 | 1.000 | 0.862 | 0.928 |
| 12430 | 0.955 | 0.872 | 0.992 | 0.943 | 1.000 | 0.862 | 0.928 |
| 12440 | 0.955 | 0.872 | 0.992 | 0.943 | 1.000 | 0.862 | 0.928 |
| 12450 | 0.955 | 0.872 | 0.992 | 0.943 | 1.000 | 0.862 | 0.928 |
| 12460 | 0.955 | 0.872 | 0.992 | 0.943 | 1.000 | 0.862 | 0.928 |
| 12470 | 0.955 | 0.872 | 0.992 | 0.943 | 1.000 | 0.862 | 0.928 |
| 12480 | 0.955 | 0.872 | 0.992 | 0.943 | 1.000 | 0.862 | 0.928 |
| 12490 | 0.955 | 0.872 | 0.992 | 0.943 | 1.000 | 0.862 | 0.928 |
| 12500 | 0.955 | 0.872 | 0.992 | 0.943 | 1.000 | 0.862 | 0.928 |
| 12510 | 0.955 | 0.872 | 0.992 | 0.943 | 1.000 | 0.862 | 0.928 |
| 12520 | 0.955 | 0.872 | 0.992 | 0.943 | 1.000 | 0.862 | 0.928 |
| 12530 | 0.951 | 0.872 | 0.992 | 0.943 | 1.000 | 0.862 | 0.928 |
| 12540 | 0.951 | 0.872 | 0.992 | 0.943 | 1.000 | 0.862 | 0.928 |
| 12550 | 0.955 | 0.872 | 0.992 | 0.943 | 1.000 | 0.862 | 0.928 |
| 12560 | 0.951 | 0.868 | 0.992 | 0.943 | 1.000 | 0.862 | 0.928 |
| 12570 | 0.951 | 0.868 | 0.992 | 0.943 | 1.000 | 0.862 | 0.928 |
| 12580 | 0.951 | 0.864 | 0.992 | 0.943 | 1.000 | 0.862 | 0.928 |
| 12590 | 0.951 | 0.864 | 0.992 | 0.943 | 1.000 | 0.862 | 0.924 |
| 12600 | 0.951 | 0.864 | 0.992 | 0.943 | 1.000 | 0.862 | 0.924 |
| 12610 | 0.951 | 0.864 | 0.992 | 0.943 | 1.000 | 0.862 | 0.928 |
| 12620 | 0.951 | 0.864 | 0.992 | 0.943 | 1.000 | 0.862 | 0.924 |
| 12630 | 0.951 | 0.864 | 0.992 | 0.943 | 1.000 | 0.862 | 0.928 |
| 12640 | 0.951 | 0.864 | 0.992 | 0.943 | 1.000 | 0.862 | 0.928 |
| 12650 | 0.951 | 0.864 | 0.992 | 0.943 | 1.000 | 0.862 | 0.928 |
| 12660 | 0.951 | 0.864 | 0.992 | 0.943 | 1.000 | 0.862 | 0.928 |
| 12670 | 0.951 | 0.864 | 0.992 | 0.943 | 1.000 | 0.862 | 0.928 |
| 12680 | 0.951 | 0.864 | 0.992 | 0.943 | 1.000 | 0.862 | 0.928 |
| 12690 | 0.951 | 0.864 | 0.992 | 0.943 | 1.000 | 0.862 | 0.928 |
| 12700 | 0.951 | 0.864 | 0.992 | 0.943 | 1.000 | 0.862 | 0.928 |
| 12710 | 0.951 | 0.864 | 0.992 | 0.943 | 1.000 | 0.862 | 0.928 |
| 12720 | 0.951 | 0.864 | 0.992 | 0.943 | 1.000 | 0.862 | 0.928 |
| 12730 | 0.951 | 0.864 | 0.992 | 0.943 | 1.000 | 0.862 | 0.928 |
| 12740 | 0.951 | 0.864 | 0.992 | 0.943 | 1.000 | 0.862 | 0.928 |
| 12750 | 0.951 | 0.864 | 0.992 | 0.943 | 1.000 | 0.862 | 0.928 |
| 12760 | 0.951 | 0.864 | 0.992 | 0.943 | 1.000 | 0.862 | 0.928 |
| 12770 | 0.951 | 0.864 | 0.992 | 0.943 | 1.000 | 0.862 | 0.928 |
| 12780 | 0.951 | 0.864 | 0.992 | 0.943 | 1.000 | 0.862 | 0.928 |
| 12790 | 0.951 | 0.864 | 0.992 | 0.943 | 1.000 | 0.862 | 0.928 |
| 12800 | 0.951 | 0.864 | 0.992 | 0.943 | 1.000 | 0.862 | 0.928 |
| 12810 | 0.959 | 0.863 | 0.992 | 0.947 | 1.000 | 0.862 | 0.923 |
| 12820 | 0.959 | 0.863 | 0.992 | 0.947 | 1.000 | 0.862 | 0.923 |
| 12830 | 0.951 | 0.867 | 0.992 | 0.947 | 1.000 | 0.858 | 0.907 |
| 12840 | 0.951 | 0.867 | 0.992 | 0.947 | 1.000 | 0.858 | 0.907 |
| 12850 | 0.951 | 0.867 | 0.992 | 0.947 | 1.000 | 0.858 | 0.907 |
| 12860 | 0.951 | 0.867 | 0.992 | 0.947 | 1.000 | 0.858 | 0.907 |
| 12870 | 0.951 | 0.867 | 0.992 | 0.947 | 1.000 | 0.858 | 0.907 |
| 12880 | 0.951 | 0.867 | 0.992 | 0.947 | 1.000 | 0.858 | 0.907 |
| 12890 | 0.951 | 0.867 | 0.992 | 0.947 | 1.000 | 0.858 | 0.907 |
| 12900 | 0.947 | 0.871 | 0.992 | 0.947 | 1.000 | 0.858 | 0.907 |
| 12910 | 0.951 | 0.867 | 0.992 | 0.947 | 1.000 | 0.858 | 0.907 |
| 12920 | 0.951 | 0.867 | 0.992 | 0.947 | 1.000 | 0.858 | 0.907 |
| 12930 | 0.951 | 0.867 | 0.992 | 0.947 | 1.000 | 0.858 | 0.907 |
| 12940 | 0.947 | 0.871 | 0.992 | 0.947 | 1.000 | 0.858 | 0.907 |
| 12950 | 0.947 | 0.871 | 0.992 | 0.947 | 1.000 | 0.858 | 0.907 |
| 12960 | 0.947 | 0.871 | 0.992 | 0.947 | 1.000 | 0.858 | 0.907 |
| 12970 | 0.947 | 0.875 | 0.992 | 0.947 | 1.000 | 0.858 | 0.907 |
| 12980 | 0.947 | 0.871 | 0.992 | 0.947 | 1.000 | 0.858 | 0.907 |
| 12990 | 0.947 | 0.875 | 0.992 | 0.947 | 1.000 | 0.858 | 0.907 |
| 13000 | 0.947 | 0.871 | 0.992 | 0.947 | 1.000 | 0.858 | 0.907 |
| 13010 | 0.947 | 0.871 | 0.992 | 0.947 | 1.000 | 0.858 | 0.907 |
| 13020 | 0.947 | 0.871 | 0.992 | 0.947 | 1.000 | 0.858 | 0.907 |
| 13030 | 0.947 | 0.871 | 0.992 | 0.947 | 1.000 | 0.858 | 0.907 |
| 13040 | 0.947 | 0.871 | 0.992 | 0.947 | 1.000 | 0.858 | 0.907 |
| 13050 | 0.947 | 0.871 | 0.992 | 0.947 | 1.000 | 0.858 | 0.907 |
| 13060 | 0.947 | 0.871 | 0.992 | 0.947 | 1.000 | 0.858 | 0.907 |
| 13070 | 0.947 | 0.871 | 0.992 | 0.947 | 1.000 | 0.858 | 0.907 |
| 13080 | 0.947 | 0.875 | 0.992 | 0.947 | 1.000 | 0.858 | 0.907 |
| 13090 | 0.947 | 0.875 | 0.992 | 0.947 | 1.000 | 0.858 | 0.907 |
| 13100 | 0.947 | 0.871 | 0.992 | 0.947 | 1.000 | 0.858 | 0.907 |
| 13110 | 0.947 | 0.871 | 0.992 | 0.947 | 1.000 | 0.858 | 0.907 |
| 13120 | 0.947 | 0.871 | 0.992 | 0.947 | 1.000 | 0.858 | 0.907 |
| 13130 | 0.942 | 0.871 | 0.992 | 0.947 | 1.000 | 0.858 | 0.911 |
| 13140 | 0.947 | 0.871 | 0.992 | 0.947 | 1.000 | 0.858 | 0.907 |
| 13150 | 0.942 | 0.871 | 0.992 | 0.947 | 1.000 | 0.858 | 0.911 |
| 13160 | 0.947 | 0.871 | 0.992 | 0.947 | 1.000 | 0.858 | 0.907 |
| 13170 | 0.947 | 0.871 | 0.992 | 0.947 | 1.000 | 0.858 | 0.907 |
| 13180 | 0.947 | 0.871 | 0.992 | 0.947 | 1.000 | 0.858 | 0.907 |
| 13190 | 0.947 | 0.871 | 0.992 | 0.947 | 1.000 | 0.858 | 0.907 |
| 13200 | 0.947 | 0.871 | 0.992 | 0.947 | 1.000 | 0.858 | 0.907 |
| 13210 | 0.951 | 0.864 | 0.992 | 0.943 | 1.000 | 0.862 | 0.920 |
| 13220 | 0.951 | 0.864 | 0.992 | 0.943 | 1.000 | 0.862 | 0.920 |
| 13230 | 0.951 | 0.864 | 0.992 | 0.943 | 1.000 | 0.862 | 0.920 |
| 13240 | 0.951 | 0.864 | 0.992 | 0.943 | 1.000 | 0.862 | 0.920 |
| 13250 | 0.951 | 0.864 | 0.992 | 0.943 | 1.000 | 0.862 | 0.920 |
| 13260 | 0.951 | 0.864 | 0.992 | 0.943 | 1.000 | 0.862 | 0.920 |
| 13270 | 0.951 | 0.864 | 0.992 | 0.943 | 1.000 | 0.862 | 0.924 |
| 13280 | 0.951 | 0.864 | 0.992 | 0.943 | 1.000 | 0.862 | 0.924 |
| 13290 | 0.951 | 0.864 | 0.992 | 0.943 | 1.000 | 0.862 | 0.924 |
| 13300 | 0.951 | 0.864 | 0.992 | 0.943 | 1.000 | 0.862 | 0.924 |
| 13310 | 0.951 | 0.864 | 0.992 | 0.943 | 1.000 | 0.862 | 0.924 |
| 13320 | 0.951 | 0.860 | 0.992 | 0.943 | 1.000 | 0.862 | 0.920 |
| 13330 | 0.951 | 0.864 | 0.992 | 0.943 | 1.000 | 0.862 | 0.920 |
| 13340 | 0.951 | 0.860 | 0.992 | 0.943 | 0.996 | 0.862 | 0.928 |
| 13350 | 0.951 | 0.860 | 0.992 | 0.943 | 0.996 | 0.862 | 0.928 |
| 13360 | 0.951 | 0.860 | 0.992 | 0.943 | 0.996 | 0.862 | 0.928 |
| 13370 | 0.951 | 0.860 | 0.992 | 0.943 | 0.996 | 0.862 | 0.928 |
| 13380 | 0.951 | 0.860 | 0.992 | 0.943 | 0.996 | 0.862 | 0.928 |
| 13390 | 0.955 | 0.860 | 0.992 | 0.943 | 0.996 | 0.862 | 0.924 |
| 13400 | 0.955 | 0.860 | 0.992 | 0.943 | 0.996 | 0.862 | 0.924 |
| 13410 | 0.955 | 0.860 | 0.992 | 0.943 | 0.996 | 0.862 | 0.928 |
| 13420 | 0.955 | 0.860 | 0.992 | 0.943 | 0.996 | 0.862 | 0.928 |
| 13430 | 0.955 | 0.860 | 0.992 | 0.943 | 0.996 | 0.862 | 0.928 |
| 13440 | 0.955 | 0.860 | 0.992 | 0.943 | 0.996 | 0.862 | 0.928 |
