# Supplementary material for: Identifying and analyzing different cancer subtypes using RNA-seq data of blood platelets
Source: Oncotarget. 2017 Sep 15;8(50):87494–511. doi: 10.18632/oncotarget.20903 (PMC5675649; doi:10.18632/oncotarget.20903)
Supplement: Supplementary file 7 [file oncotarget-08-87494-s007.docx]

**Supplementary Table 6:** **The specificity for each class yielded by the second stage of the IFS method**

| **Number of features** | **Breast cancer** | **Colorectal cancer** | **Glioblastoma** | **Healthy control** | **Hepatobiliary cancer** | **Lung cancer** | **Pancreas cancer** |
| --- | --- | --- | --- | --- | --- | --- | --- |
| 2000 | 0.931 | 0.922 | 0.988 | 0.948 | 0.996 | 0.920 | 0.956 |
| 2001 | 0.931 | 0.922 | 0.988 | 0.948 | 0.996 | 0.920 | 0.956 |
| 2002 | 0.931 | 0.922 | 0.988 | 0.948 | 0.996 | 0.920 | 0.956 |
| 2003 | 0.931 | 0.922 | 0.988 | 0.948 | 0.996 | 0.920 | 0.956 |
| 2004 | 0.931 | 0.922 | 0.988 | 0.948 | 0.996 | 0.920 | 0.956 |
| 2005 | 0.927 | 0.926 | 0.988 | 0.948 | 0.996 | 0.920 | 0.956 |
| 2006 | 0.927 | 0.926 | 0.988 | 0.948 | 0.996 | 0.920 | 0.956 |
| 2007 | 0.927 | 0.926 | 0.988 | 0.948 | 0.996 | 0.920 | 0.956 |
| 2008 | 0.927 | 0.926 | 0.988 | 0.948 | 0.996 | 0.920 | 0.956 |
| 2009 | 0.927 | 0.926 | 0.992 | 0.952 | 0.996 | 0.920 | 0.956 |
| 2010 | 0.927 | 0.926 | 0.992 | 0.952 | 0.996 | 0.920 | 0.956 |
| 2011 | 0.927 | 0.926 | 0.992 | 0.952 | 0.996 | 0.920 | 0.956 |
| 2012 | 0.927 | 0.926 | 0.992 | 0.952 | 0.996 | 0.920 | 0.956 |
| 2013 | 0.935 | 0.922 | 0.992 | 0.952 | 0.996 | 0.920 | 0.956 |
| 2014 | 0.935 | 0.922 | 0.992 | 0.952 | 0.996 | 0.920 | 0.956 |
| 2015 | 0.939 | 0.922 | 0.992 | 0.952 | 0.996 | 0.920 | 0.956 |
| 2016 | 0.939 | 0.922 | 0.992 | 0.952 | 0.996 | 0.920 | 0.960 |
| 2017 | 0.935 | 0.922 | 0.992 | 0.952 | 0.996 | 0.920 | 0.964 |
| 2018 | 0.935 | 0.922 | 0.992 | 0.952 | 0.996 | 0.920 | 0.964 |
| 2019 | 0.935 | 0.918 | 0.992 | 0.952 | 0.996 | 0.920 | 0.960 |
| 2020 | 0.935 | 0.918 | 0.992 | 0.952 | 0.996 | 0.920 | 0.960 |
| 2021 | 0.935 | 0.918 | 0.992 | 0.948 | 0.996 | 0.920 | 0.964 |
| 2022 | 0.935 | 0.918 | 0.992 | 0.948 | 0.996 | 0.920 | 0.964 |
| 2023 | 0.931 | 0.922 | 0.992 | 0.948 | 0.996 | 0.920 | 0.964 |
| 2024 | 0.939 | 0.930 | 0.992 | 0.948 | 0.996 | 0.920 | 0.964 |
| 2025 | 0.935 | 0.930 | 0.992 | 0.948 | 0.996 | 0.920 | 0.964 |
| 2026 | 0.939 | 0.930 | 0.992 | 0.948 | 0.996 | 0.920 | 0.964 |
| 2027 | 0.935 | 0.930 | 0.992 | 0.948 | 0.996 | 0.920 | 0.968 |
| 2028 | 0.935 | 0.934 | 0.992 | 0.948 | 0.996 | 0.920 | 0.964 |
| 2029 | 0.935 | 0.934 | 0.992 | 0.948 | 0.996 | 0.920 | 0.968 |
| 2030 | 0.935 | 0.934 | 0.992 | 0.952 | 0.996 | 0.920 | 0.968 |
| 2031 | 0.935 | 0.934 | 0.992 | 0.952 | 0.996 | 0.920 | 0.968 |
| 2032 | 0.935 | 0.934 | 0.992 | 0.952 | 0.996 | 0.920 | 0.968 |
| 2033 | 0.935 | 0.934 | 0.992 | 0.952 | 0.996 | 0.920 | 0.968 |
| 2034 | 0.935 | 0.938 | 0.992 | 0.952 | 0.996 | 0.920 | 0.964 |
| 2035 | 0.931 | 0.934 | 0.992 | 0.952 | 0.996 | 0.929 | 0.964 |
| 2036 | 0.935 | 0.930 | 0.992 | 0.952 | 0.996 | 0.924 | 0.964 |
| 2037 | 0.935 | 0.930 | 0.992 | 0.952 | 0.996 | 0.924 | 0.964 |
| 2038 | 0.935 | 0.930 | 0.992 | 0.952 | 0.996 | 0.924 | 0.964 |
| 2039 | 0.935 | 0.934 | 0.992 | 0.952 | 0.996 | 0.924 | 0.960 |
| 2040 | 0.935 | 0.934 | 0.992 | 0.952 | 0.996 | 0.924 | 0.960 |
| 2041 | 0.935 | 0.934 | 0.992 | 0.952 | 0.996 | 0.924 | 0.960 |
| 2042 | 0.935 | 0.934 | 0.992 | 0.952 | 0.996 | 0.924 | 0.960 |
| 2043 | 0.935 | 0.934 | 0.992 | 0.952 | 0.996 | 0.924 | 0.960 |
| 2044 | 0.935 | 0.930 | 0.992 | 0.952 | 0.996 | 0.924 | 0.960 |
| 2045 | 0.935 | 0.930 | 0.992 | 0.952 | 0.996 | 0.924 | 0.960 |
| 2046 | 0.939 | 0.930 | 0.992 | 0.952 | 0.996 | 0.924 | 0.964 |
| 2047 | 0.939 | 0.934 | 0.992 | 0.952 | 0.996 | 0.924 | 0.964 |
| 2048 | 0.935 | 0.934 | 0.992 | 0.952 | 0.996 | 0.924 | 0.964 |
| 2049 | 0.939 | 0.934 | 0.992 | 0.948 | 0.996 | 0.924 | 0.960 |
| 2050 | 0.935 | 0.934 | 0.992 | 0.948 | 0.996 | 0.924 | 0.960 |
| 2051 | 0.935 | 0.934 | 0.992 | 0.948 | 0.996 | 0.924 | 0.960 |
| 2052 | 0.935 | 0.934 | 0.992 | 0.948 | 0.996 | 0.924 | 0.960 |
| 2053 | 0.935 | 0.930 | 0.992 | 0.948 | 0.996 | 0.924 | 0.964 |
| 2054 | 0.935 | 0.930 | 0.992 | 0.948 | 0.996 | 0.924 | 0.964 |
| 2055 | 0.935 | 0.934 | 0.992 | 0.948 | 0.996 | 0.924 | 0.960 |
| 2056 | 0.935 | 0.934 | 0.992 | 0.948 | 0.996 | 0.924 | 0.956 |
| 2057 | 0.935 | 0.930 | 0.992 | 0.952 | 0.996 | 0.924 | 0.960 |
| 2058 | 0.935 | 0.930 | 0.992 | 0.952 | 0.996 | 0.924 | 0.960 |
| 2059 | 0.931 | 0.934 | 0.992 | 0.952 | 0.996 | 0.924 | 0.956 |
| 2060 | 0.931 | 0.930 | 0.992 | 0.952 | 0.996 | 0.924 | 0.956 |
| 2061 | 0.931 | 0.930 | 0.992 | 0.952 | 0.996 | 0.924 | 0.956 |
| 2062 | 0.931 | 0.930 | 0.992 | 0.952 | 0.996 | 0.924 | 0.956 |
| 2063 | 0.931 | 0.930 | 0.992 | 0.952 | 0.996 | 0.924 | 0.956 |
| 2064 | 0.931 | 0.926 | 0.992 | 0.952 | 0.996 | 0.929 | 0.956 |
| 2065 | 0.931 | 0.926 | 0.992 | 0.952 | 0.996 | 0.929 | 0.956 |
| 2066 | 0.931 | 0.926 | 0.992 | 0.952 | 0.996 | 0.929 | 0.960 |
| 2067 | 0.931 | 0.926 | 0.992 | 0.952 | 0.996 | 0.929 | 0.956 |
| 2068 | 0.931 | 0.926 | 0.992 | 0.952 | 0.996 | 0.929 | 0.956 |
| 2069 | 0.931 | 0.926 | 0.992 | 0.952 | 0.996 | 0.929 | 0.956 |
| 2070 | 0.931 | 0.926 | 0.992 | 0.952 | 0.996 | 0.929 | 0.956 |
| 2071 | 0.931 | 0.926 | 0.992 | 0.952 | 0.996 | 0.929 | 0.956 |
| 2072 | 0.931 | 0.926 | 0.992 | 0.952 | 0.996 | 0.929 | 0.956 |
| 2073 | 0.931 | 0.934 | 0.992 | 0.952 | 0.996 | 0.929 | 0.952 |
| 2074 | 0.931 | 0.930 | 0.992 | 0.952 | 0.996 | 0.929 | 0.952 |
| 2075 | 0.931 | 0.926 | 0.992 | 0.952 | 0.996 | 0.929 | 0.956 |
| 2076 | 0.931 | 0.926 | 0.992 | 0.952 | 0.996 | 0.929 | 0.956 |
| 2077 | 0.931 | 0.926 | 0.992 | 0.952 | 0.996 | 0.929 | 0.956 |
| 2078 | 0.931 | 0.934 | 0.992 | 0.952 | 0.996 | 0.929 | 0.952 |
| 2079 | 0.931 | 0.930 | 0.992 | 0.952 | 0.996 | 0.929 | 0.948 |
| 2080 | 0.931 | 0.934 | 0.992 | 0.952 | 0.996 | 0.929 | 0.952 |
| 2081 | 0.931 | 0.934 | 0.992 | 0.952 | 0.996 | 0.929 | 0.952 |
| 2082 | 0.931 | 0.934 | 0.992 | 0.952 | 0.996 | 0.929 | 0.952 |
| 2083 | 0.931 | 0.934 | 0.992 | 0.952 | 0.996 | 0.929 | 0.952 |
| 2084 | 0.931 | 0.934 | 0.992 | 0.952 | 0.996 | 0.929 | 0.952 |
| 2085 | 0.931 | 0.930 | 0.992 | 0.952 | 0.996 | 0.929 | 0.952 |
| 2086 | 0.931 | 0.930 | 0.992 | 0.952 | 0.996 | 0.929 | 0.952 |
| 2087 | 0.931 | 0.930 | 0.992 | 0.952 | 0.996 | 0.929 | 0.952 |
| 2088 | 0.931 | 0.930 | 0.992 | 0.952 | 0.996 | 0.929 | 0.952 |
| 2089 | 0.931 | 0.930 | 0.992 | 0.952 | 0.996 | 0.929 | 0.952 |
| 2090 | 0.931 | 0.930 | 0.992 | 0.952 | 0.996 | 0.929 | 0.952 |
| 2091 | 0.931 | 0.934 | 0.992 | 0.952 | 0.996 | 0.929 | 0.952 |
| 2092 | 0.931 | 0.930 | 0.992 | 0.952 | 0.996 | 0.929 | 0.952 |
| 2093 | 0.935 | 0.922 | 0.992 | 0.952 | 0.996 | 0.929 | 0.952 |
| 2094 | 0.935 | 0.922 | 0.992 | 0.952 | 0.996 | 0.929 | 0.952 |
| 2095 | 0.935 | 0.926 | 0.992 | 0.952 | 0.996 | 0.929 | 0.952 |
| 2096 | 0.935 | 0.922 | 0.992 | 0.952 | 0.996 | 0.929 | 0.952 |
| 2097 | 0.935 | 0.922 | 0.992 | 0.952 | 0.996 | 0.929 | 0.952 |
| 2098 | 0.939 | 0.922 | 0.992 | 0.952 | 0.996 | 0.929 | 0.952 |
| 2099 | 0.939 | 0.922 | 0.992 | 0.952 | 0.996 | 0.929 | 0.952 |
| 2100 | 0.939 | 0.922 | 0.992 | 0.952 | 0.996 | 0.929 | 0.952 |
| 2101 | 0.935 | 0.922 | 0.992 | 0.952 | 0.996 | 0.929 | 0.952 |
| 2102 | 0.935 | 0.922 | 0.992 | 0.952 | 0.996 | 0.929 | 0.956 |
| 2103 | 0.935 | 0.922 | 0.992 | 0.952 | 0.996 | 0.929 | 0.956 |
| 2104 | 0.935 | 0.922 | 0.992 | 0.952 | 0.996 | 0.929 | 0.956 |
| 2105 | 0.935 | 0.922 | 0.992 | 0.952 | 0.996 | 0.929 | 0.956 |
| 2106 | 0.935 | 0.922 | 0.992 | 0.952 | 0.996 | 0.929 | 0.952 |
| 2107 | 0.935 | 0.922 | 0.992 | 0.952 | 0.996 | 0.929 | 0.952 |
| 2108 | 0.935 | 0.922 | 0.992 | 0.952 | 0.996 | 0.929 | 0.952 |
| 2109 | 0.935 | 0.922 | 0.992 | 0.952 | 0.996 | 0.929 | 0.956 |
| 2110 | 0.939 | 0.922 | 0.992 | 0.952 | 0.996 | 0.929 | 0.956 |
| 2111 | 0.939 | 0.922 | 0.992 | 0.952 | 0.996 | 0.929 | 0.956 |
| 2112 | 0.939 | 0.922 | 0.992 | 0.952 | 0.996 | 0.929 | 0.956 |
| 2113 | 0.939 | 0.922 | 0.992 | 0.952 | 0.996 | 0.929 | 0.956 |
| 2114 | 0.943 | 0.918 | 0.992 | 0.952 | 0.996 | 0.929 | 0.952 |
| 2115 | 0.943 | 0.918 | 0.992 | 0.952 | 0.996 | 0.929 | 0.952 |
| 2116 | 0.943 | 0.918 | 0.992 | 0.952 | 0.996 | 0.929 | 0.956 |
| 2117 | 0.943 | 0.918 | 0.992 | 0.952 | 0.996 | 0.929 | 0.956 |
| 2118 | 0.943 | 0.918 | 0.992 | 0.952 | 0.996 | 0.929 | 0.956 |
| 2119 | 0.939 | 0.918 | 0.992 | 0.952 | 0.996 | 0.929 | 0.956 |
| 2120 | 0.939 | 0.918 | 0.992 | 0.952 | 0.996 | 0.929 | 0.956 |
| 2121 | 0.939 | 0.918 | 0.992 | 0.952 | 0.996 | 0.929 | 0.956 |
| 2122 | 0.939 | 0.926 | 0.992 | 0.952 | 0.996 | 0.929 | 0.956 |
| 2123 | 0.939 | 0.926 | 0.992 | 0.952 | 0.996 | 0.929 | 0.956 |
| 2124 | 0.939 | 0.926 | 0.992 | 0.952 | 0.996 | 0.929 | 0.956 |
| 2125 | 0.939 | 0.926 | 0.992 | 0.952 | 0.996 | 0.929 | 0.956 |
| 2126 | 0.943 | 0.918 | 0.992 | 0.952 | 0.996 | 0.929 | 0.960 |
| 2127 | 0.943 | 0.918 | 0.992 | 0.952 | 0.996 | 0.929 | 0.960 |
| 2128 | 0.943 | 0.918 | 0.992 | 0.952 | 0.996 | 0.929 | 0.960 |
| 2129 | 0.939 | 0.926 | 0.992 | 0.952 | 0.996 | 0.929 | 0.960 |
| 2130 | 0.939 | 0.926 | 0.992 | 0.952 | 0.996 | 0.929 | 0.960 |
| 2131 | 0.939 | 0.926 | 0.992 | 0.952 | 0.996 | 0.929 | 0.960 |
| 2132 | 0.935 | 0.922 | 0.992 | 0.952 | 0.996 | 0.929 | 0.960 |
| 2133 | 0.935 | 0.922 | 0.992 | 0.952 | 0.996 | 0.929 | 0.960 |
| 2134 | 0.935 | 0.922 | 0.992 | 0.952 | 0.996 | 0.929 | 0.960 |
| 2135 | 0.935 | 0.922 | 0.992 | 0.952 | 0.996 | 0.929 | 0.960 |
| 2136 | 0.939 | 0.918 | 0.992 | 0.952 | 0.996 | 0.924 | 0.956 |
| 2137 | 0.939 | 0.914 | 0.992 | 0.952 | 0.996 | 0.924 | 0.960 |
| 2138 | 0.939 | 0.914 | 0.992 | 0.952 | 0.996 | 0.924 | 0.960 |
| 2139 | 0.939 | 0.914 | 0.992 | 0.952 | 0.996 | 0.924 | 0.960 |
| 2140 | 0.939 | 0.914 | 0.992 | 0.952 | 0.996 | 0.924 | 0.960 |
| 2141 | 0.939 | 0.914 | 0.992 | 0.952 | 0.996 | 0.924 | 0.960 |
| 2142 | 0.939 | 0.914 | 0.992 | 0.952 | 0.996 | 0.924 | 0.960 |
| 2143 | 0.935 | 0.914 | 0.992 | 0.952 | 0.996 | 0.924 | 0.968 |
| 2144 | 0.935 | 0.914 | 0.992 | 0.952 | 0.996 | 0.924 | 0.968 |
| 2145 | 0.935 | 0.914 | 0.992 | 0.952 | 0.996 | 0.924 | 0.968 |
| 2146 | 0.935 | 0.914 | 0.992 | 0.952 | 0.996 | 0.924 | 0.968 |
| 2147 | 0.935 | 0.914 | 0.992 | 0.952 | 0.996 | 0.924 | 0.968 |
| 2148 | 0.935 | 0.909 | 0.992 | 0.952 | 0.996 | 0.924 | 0.968 |
| 2149 | 0.935 | 0.909 | 0.992 | 0.952 | 0.996 | 0.924 | 0.968 |
| 2150 | 0.935 | 0.909 | 0.992 | 0.952 | 0.996 | 0.929 | 0.968 |
| 2151 | 0.935 | 0.909 | 0.992 | 0.952 | 0.996 | 0.929 | 0.964 |
| 2152 | 0.935 | 0.909 | 0.992 | 0.952 | 0.996 | 0.929 | 0.964 |
| 2153 | 0.935 | 0.909 | 0.992 | 0.952 | 0.996 | 0.929 | 0.964 |
| 2154 | 0.935 | 0.905 | 0.992 | 0.952 | 0.996 | 0.929 | 0.964 |
| 2155 | 0.935 | 0.905 | 0.992 | 0.952 | 0.996 | 0.929 | 0.964 |
| 2156 | 0.939 | 0.905 | 0.992 | 0.952 | 0.996 | 0.929 | 0.964 |
| 2157 | 0.939 | 0.914 | 0.992 | 0.952 | 0.996 | 0.929 | 0.964 |
| 2158 | 0.939 | 0.914 | 0.992 | 0.952 | 0.996 | 0.929 | 0.964 |
| 2159 | 0.939 | 0.914 | 0.992 | 0.952 | 0.996 | 0.929 | 0.964 |
| 2160 | 0.939 | 0.909 | 0.992 | 0.952 | 0.996 | 0.929 | 0.964 |
| 2161 | 0.931 | 0.918 | 0.992 | 0.952 | 0.996 | 0.929 | 0.968 |
| 2162 | 0.931 | 0.918 | 0.992 | 0.952 | 0.996 | 0.929 | 0.968 |
| 2163 | 0.931 | 0.918 | 0.992 | 0.952 | 0.996 | 0.929 | 0.968 |
| 2164 | 0.935 | 0.909 | 0.992 | 0.952 | 0.996 | 0.929 | 0.968 |
| 2165 | 0.935 | 0.909 | 0.992 | 0.952 | 0.996 | 0.929 | 0.968 |
| 2166 | 0.935 | 0.909 | 0.992 | 0.952 | 0.996 | 0.929 | 0.968 |
| 2167 | 0.935 | 0.909 | 0.992 | 0.952 | 0.996 | 0.924 | 0.972 |
| 2168 | 0.935 | 0.909 | 0.992 | 0.952 | 0.996 | 0.929 | 0.972 |
| 2169 | 0.935 | 0.909 | 0.992 | 0.952 | 0.996 | 0.929 | 0.972 |
| 2170 | 0.931 | 0.918 | 0.992 | 0.952 | 0.996 | 0.929 | 0.972 |
| 2171 | 0.931 | 0.918 | 0.992 | 0.952 | 0.996 | 0.929 | 0.972 |
| 2172 | 0.935 | 0.918 | 0.992 | 0.952 | 0.996 | 0.929 | 0.972 |
| 2173 | 0.935 | 0.918 | 0.992 | 0.952 | 0.996 | 0.929 | 0.972 |
| 2174 | 0.935 | 0.918 | 0.992 | 0.952 | 0.996 | 0.929 | 0.972 |
| 2175 | 0.935 | 0.918 | 0.992 | 0.952 | 0.996 | 0.924 | 0.968 |
| 2176 | 0.935 | 0.918 | 0.992 | 0.952 | 0.996 | 0.924 | 0.964 |
| 2177 | 0.935 | 0.918 | 0.992 | 0.952 | 0.996 | 0.924 | 0.964 |
| 2178 | 0.935 | 0.918 | 0.992 | 0.952 | 0.996 | 0.924 | 0.964 |
| 2179 | 0.935 | 0.918 | 0.992 | 0.952 | 0.996 | 0.924 | 0.964 |
| 2180 | 0.935 | 0.918 | 0.992 | 0.952 | 0.996 | 0.924 | 0.964 |
| 2181 | 0.935 | 0.918 | 0.992 | 0.952 | 0.996 | 0.924 | 0.964 |
| 2182 | 0.935 | 0.914 | 0.992 | 0.952 | 0.996 | 0.924 | 0.964 |
| 2183 | 0.935 | 0.914 | 0.992 | 0.952 | 0.996 | 0.924 | 0.964 |
| 2184 | 0.935 | 0.914 | 0.992 | 0.952 | 0.996 | 0.924 | 0.964 |
| 2185 | 0.935 | 0.914 | 0.992 | 0.952 | 0.996 | 0.924 | 0.964 |
| 2186 | 0.935 | 0.914 | 0.992 | 0.952 | 0.996 | 0.924 | 0.964 |
| 2187 | 0.935 | 0.914 | 0.992 | 0.952 | 0.996 | 0.924 | 0.964 |
| 2188 | 0.935 | 0.914 | 0.992 | 0.952 | 0.996 | 0.924 | 0.964 |
| 2189 | 0.935 | 0.918 | 0.992 | 0.952 | 0.996 | 0.924 | 0.964 |
| 2190 | 0.935 | 0.918 | 0.992 | 0.952 | 0.996 | 0.924 | 0.964 |
| 2191 | 0.935 | 0.918 | 0.992 | 0.952 | 0.996 | 0.924 | 0.964 |
| 2192 | 0.939 | 0.922 | 0.992 | 0.952 | 0.996 | 0.924 | 0.956 |
| 2193 | 0.939 | 0.914 | 0.992 | 0.952 | 0.996 | 0.924 | 0.956 |
| 2194 | 0.939 | 0.914 | 0.992 | 0.952 | 0.996 | 0.924 | 0.956 |
| 2195 | 0.939 | 0.914 | 0.992 | 0.952 | 0.996 | 0.924 | 0.956 |
| 2196 | 0.939 | 0.918 | 0.992 | 0.952 | 0.996 | 0.920 | 0.956 |
| 2197 | 0.939 | 0.918 | 0.992 | 0.952 | 0.996 | 0.920 | 0.956 |
| 2198 | 0.939 | 0.909 | 0.992 | 0.952 | 0.996 | 0.920 | 0.960 |
| 2199 | 0.939 | 0.909 | 0.992 | 0.952 | 0.996 | 0.920 | 0.960 |
| 2200 | 0.939 | 0.909 | 0.992 | 0.952 | 0.996 | 0.920 | 0.960 |
