# Supplementary material for: Identifying and analyzing different cancer subtypes using RNA-seq data of blood platelets
Source: Oncotarget. 2017 Sep 15;8(50):87494–511. doi: 10.18632/oncotarget.20903 (PMC5675649; doi:10.18632/oncotarget.20903)
Supplement: Supplementary file 8 [file oncotarget-08-87494-s008.docx]

**Supplementary Table 7: Cancer panel genes in seven commercial cancer detection panels**

| **CancerNext** | **CancerNextExpanded** | **CloudHealth** | **GeneDx** | **Illumina** | **NanoString** | **xGen** |
| --- | --- | --- | --- | --- | --- | --- |
| APC | AIP | BRCA1 | APC | ABCC3 | ABL1 | ACVR1B |
| ATM | ALK | BRCA2 | ATM | ABI1 | ACVR1B | ACVR2A |
| BARD1 | APC | BRCA1 | AXIN2 | ABL1 | ACVR1C | AJUBA |
| BRCA1 | ATM | BRCA2 | BARD1 | ABL2 | ACVR2A | AKT1 |
| BRCA2 | BAP1 | PTEN | BMPR1A | ABLIM1 | AKT1 | APC |
| BRIP1 | BARD1 | TP53 | BRCA1 | ACACA | AKT2 | AR |
| BMPR1A | BLM | CDH1 | BRCA2 | ACE | AKT3 | ARHGAP35 |
| CDH1 | BRCA1 | ATM | BRIP1 | ACER1 | ALK | ARID1A |
| CDK4 | BRCA2 | BRIP1 | CDH1 | ACKR3 | ALKBH2 | ARID5B |
| CDKN2A | BRIP1 | CHEK2 | CDK4 | ACSBG1 | ALKBH3 | ASXL1 |
| CHEK2 | BMPR1A | MSH2 | CDKN2A | ACSL3 | AMER1 | ATM |
| EPCAM | CDH1 | MLH1 | CHEK2 | ACSL6 | AMH | ATR |
| DICER1 | CDK4 | MSH6 | EPCAM | ACVR1B | ANGPT1 | ATRX |
| GREM1 | CDKN1B | PMS2 | FANCC | ACVR1C | APC | AXIN2 |
| HOXB13 | CDKN2A | EPCAM | MLH1 | ACVR2A | APH1B | B4GALT3 |
| MLH1 | CHEK2 | NBN | MSH2 | ADD3 | AR | BAP1 |
| MRE11A | DICER1 | NF1 | MSH6 | ADM | ARID1A | BRAF |
| MSH2 | EPCAM | PALB2 | MUTYH | AFF1 | ARID1B | BRCA1 |
| MSH6 | FANCC | RAD51C | NBN | AFF3 | ARID2 | BRCA2 |
| MUTYH | FH | RAD51D | PALB2 | AFF4 | ARNT2 | CBFB |
| NBN | FLCN | STK11 | PMS2 | AGR3 | ASXL1 | CCND1 |
| NF1 | GALNT12 | BARD1 | POLD1 | AHCYL1 | ATM | CDH1 |
| PALB2 | GREM1 | FANCC | POLE | AHI1 | ATR | CDK12 |
| PMS2 | HOXB13 | MRE11A | PTEN | AHR | ATRX | CDKN1A |
| POLD1 | MAX | MUTYH | RAD51C | AHRR | AXIN1 | CDKN1B |
| POLE | MEN1 | XRCC2 | RAD51D | AIP | AXIN2 | CDKN2A |
| PTEN | MET | SMARCA | SCG5 | AK2 | B2M | CDKN2C |
| RAD50 | MITF | RAD50 | GREM1 | AK5 | BAD | CEBPA |
| RAD51C | MLH1 | APC | SMAD4 | AKAP12 | BAIAP3 | CHEK2 |
| RAD51D | MRE11A | BMPR1A | STK11 | AKAP6 | BAMBI | CRIPAK |
| SMAD4 | MSH2 | EPCAM | TP53 | AKAP9 | BAP1 | CTCF |
| SMARCA4 | MSH6 | MLH1 | VHL | AKR1C3 | BAX | CTNNB1 |
| STK11 | MUTYH | MSH2 | XRCC2 | AKT1 | BCL2 | DNMT3A |
| TP53 | NBN | MSH6 |  | AKT2 | BCL2A1 | EGFR |
|  | NF1 | MUTYH |  | AKT3 | BCL2L1 | EGR3 |
|  | NF2 | PMS2 |  | ALDH1A1 | BCOR | EIF4A2 |
|  | PALB2 | PTEN |  | ALDH2 | BDNF | ELF3 |
|  | PHOX2B | SMAD4 |  | ALDOC | BID | EP300 |
|  | PMS2 | TP53 |  | ALK | BIRC3 | EPHA3 |
|  | POLD1 | CDH1 |  | AMER1 | BIRC7 | EPHB6 |
|  | POLE | APC |  | AMH | BMP2 | EPPK1 |
|  | POT1 | ATM |  | ANGPT1 | BMP4 | ERBB4 |
|  | PRKAR1A | AXIN2 |  | ANKRD28 | BMP5 | ERCC2 |
|  | PTCH1 | BLM |  | ANLN | BMP6 | EZH2 |
|  | PTEN | BMPR1A |  | APC | BMP7 | FBXW7 |
|  | RAD50 | CHEK2 |  | APH1A | BMP8A | FGFR2 |
|  | RAD51C | EPCAM |  | APLP2 | BMPR1B | FGFR3 |
|  | RAD51D | EXO1 |  | APOD | BNIP3 | FLT3 |
|  | RB1 | GALNT12 |  | AR | BRAF | FOXA1 |
|  | RET | GREM1 |  | ARAF | BRCA1 | FOXA2 |
|  | SDHA | LH3 |  | ARFRP1 | BRCA2 | GATA3 |
|  | SDHAF2 | MLH1 |  | ARHGAP20 | BRIP1 | H3F3C |
|  | SDHB | MSH2 |  | ARHGAP26 | C19orf40 | HGF |
|  | SDHC | MSH6 |  | ARHGEF12 | CACNA1C | HIST1H1C |
|  | SDHD | MUTYH |  | ARHGEF7 | CACNA1D | HIST1H2BD |
|  | SMAD4 | NTHL1 |  | ARID1A | CACNA1E | IDH1 |
|  | SMARCA4 | PMS1 |  | ARID2 | CACNA1G | IDH2 |
|  | SMARCB1 | PMS2 |  | ARIH2 | CACNA1H | KDM5C |
|  | SMARCE1 | POLD1 |  | ARNT | CACNA2D1 | KDM6A |
|  | STK11 | POLE |  | ARRDC4 | CACNA2D2 | KEAP1 |
|  | SUFU | PTEN |  | ASMTL | CACNA2D3 | KIT |
|  | TMEM127 | SMAD4 |  | ASPH | CACNA2D4 | KRAS |
|  | TP53 | STK11 |  | ASPSCR1 | CACNB2 | LIFR |
|  | TSC1 | TP53 |  | ASTN2 | CACNB3 | LRRK2 |
|  | TSC2 | BRCA1 |  | ASXL1 | CACNB4 | MALAT1 |
|  | VHL | BRCA2 |  | ATF1 | CACNG1 | MAP2K4 |
|  | XRCC2 | PALB2 |  | ATF3 | CACNG4 | MAP3K1 |
|  |  | FANCC |  | ATG13 | CACNG6 | MAPK8IP1 |
|  |  | FANCG |  | ATG5 | CALML3 | MECOM |
|  |  | ATM |  | ATIC | CALML5 | MIR142 |
|  |  | PRSS1 |  | ATL1 | CALML6 | KMT2B |
|  |  | SPINK1 |  | ATM | CAMK2B | KMT2C |
|  |  | CTFR |  | ATP1B4 | CAPN2 | KMT2D |
|  |  | MLH1 |  | ATP8A2 | CARD11 | MTOR |
|  |  | MSH2 |  | ATR | CASP10 | NAV3 |
|  |  | MSH6 |  | ATRNL1 | CASP12 | NCOR1 |
|  |  | PMS2 |  | ATRX | CASP3 | NF1 |
|  |  | EPCAM |  | AURKA | CASP7 | NFE2L2 |
|  |  | STK11 |  | AURKB | CASP8 | NFE2L3 |
|  |  | VHL |  | AUTS2 | CASP9 | NOTCH1 |
|  |  | CDKN2A |  | AXIN1 | CBL | NPM1 |
|  |  | CDK4 |  | AXL | CBLC | NRAS |
|  |  | CDKN2A |  | BACH1 | CCNA1 | NSD1 |
|  |  | CDK4 |  | BACH2 | CCNA2 | PBRM1 |
|  |  | TP53 |  | BAG4 | CCNB1 | PCBP1 |
|  |  | PTEN |  | BAIAP2L1 | CCNB3 | PDGFRA |
|  |  | BRCA2 |  | BAP1 | CCND1 | PHF6 |
|  |  | BRCA2 |  | BARD1 | CCND2 | PIK3CA |
|  |  | BRCA1 |  | BAX | CCND3 | PIK3CG |
|  |  | CHEK2 |  | BAZ2A | CCNE1 | PIK3R1 |
|  |  | TP53 |  | BCAS3 | CCNE2 | POLQ |
|  |  | NBN |  | BCAS4 | CCNO | PPP2R1A |
|  |  | RET |  | BCL10 | CCR7 | PRX |
|  |  | VHL |  | BCL11A | CD14 | PTEN |
|  |  | TSC1 |  | BCL11B | CD19 | PTPN11 |
|  |  | TSC2 |  | BCL2 | CD40 | RAD21 |
|  |  | FLCN |  | BCL2A1 | CDC14A | RB1 |
|  |  | MET |  | BCL2L1 | CDC14B | RPL22 |
|  |  | FH |  | BCL2L2 | CDC25A | RPL5 |
|  |  | RB1 |  | BCL3 | CDC25B | RUNX1 |
|  |  | TP53 |  | BCL6 | CDC25C | SETBP1 |
|  |  | PTEN |  | BCL7A | CDC6 | SETD2 |
|  |  | CHEK2 |  | BCL9 | CDC7 | SF3B1 |
|  |  | MEN1 |  | BCOR | CDH1 | SIN3A |
|  |  |  |  | BCORL1 | CDK2 | SMAD2 |
|  |  |  |  | BCR | CDK4 | SMAD4 |
|  |  |  |  | BDNF | CDK6 | SMC1A |
|  |  |  |  | BHLHE22 | CDKN1A | SMC3 |
|  |  |  |  | BICC1 | CDKN1B | SOX17 |
|  |  |  |  | BIN1 | CDKN1C | SOX9 |
|  |  |  |  | BIRC3 | CDKN2A | SPOP |
|  |  |  |  | BIRC6 | CDKN2B | STAG2 |
|  |  |  |  | BLM | CDKN2C | STK11 |
|  |  |  |  | BMP4 | CDKN2D | TAF1 |
|  |  |  |  | BMPR1A | CEBPA | TBL1XR1 |
|  |  |  |  | BRAF | CEBPE | TBX3 |
|  |  |  |  | BRCA1 | CHAD | TET2 |
|  |  |  |  | BRCA2 | CHEK1 | TGFBR2 |
|  |  |  |  | BRD1 | CHEK2 | TLR4 |
|  |  |  |  | BRD3 | CHUK | TP53 |
|  |  |  |  | BRD4 | CIC | TSHZ2 |
|  |  |  |  | BRIP1 | CLCF1 | TSHZ3 |
|  |  |  |  | BRSK1 | CNTFR | U2AF1 |
|  |  |  |  | BRWD3 | COL11A1 | USP9X |
|  |  |  |  | BTBD18 | COL11A2 | VEZF1 |
|  |  |  |  | BTG1 | COL1A1 | VHL |
|  |  |  |  | BTG2 | COL1A2 | WT1 |
|  |  |  |  | BTK | COL24A1 |  |
|  |  |  |  | BTLA | COL27A1 |  |
|  |  |  |  | BUB1B | COL2A1 |  |
|  |  |  |  | C11orf1 | COL3A1 |  |
|  |  |  |  | C11orf30 | COL4A3 |  |
|  |  |  |  | C11orf54 | COL4A4 |  |
|  |  |  |  | C11orf95 | COL4A5 |  |
|  |  |  |  | C2CD2L | COL4A6 |  |
|  |  |  |  | C2orf44 | COL5A1 |  |
|  |  |  |  | C3orf27 | COL5A2 |  |
|  |  |  |  | CACNA1F | COL6A6 |  |
|  |  |  |  | CACNA1G | COMP |  |
|  |  |  |  | CACNA2D3 | CREB3L1 |  |
|  |  |  |  | CAD | CREB3L3 |  |
|  |  |  |  | CALR | CREB3L4 |  |
|  |  |  |  | CAMK2A | CREB5 |  |
|  |  |  |  | CAMK2B | CREBBP |  |
|  |  |  |  | CAMK2G | CRLF2 |  |
|  |  |  |  | CAMTA1 | CSF1R |  |
|  |  |  |  | CANT1 | CSF2 |  |
|  |  |  |  | CAPRIN1 | CSF3 |  |
|  |  |  |  | CAPZB | CSF3R |  |
|  |  |  |  | CARD11 | CTNNB1 |  |
|  |  |  |  | CARM1 | CUL1 |  |
|  |  |  |  | CARS | CXXC4 |  |
|  |  |  |  | CASC5 | CYLD |  |
|  |  |  |  | CASP3 | DAXX |  |
|  |  |  |  | CASP7 | DDB2 |  |
|  |  |  |  | CASP8 | DDIT3 |  |
|  |  |  |  | CAV1 | DDIT4 |  |
|  |  |  |  | CBFA2T3 | DKK1 |  |
|  |  |  |  | CBFB | DKK2 |  |
|  |  |  |  | CBL | DKK4 |  |
|  |  |  |  | CBLB | DLL1 |  |
|  |  |  |  | CBLC | DLL3 |  |
|  |  |  |  | CCAR2 | DLL4 |  |
|  |  |  |  | CCDC28A | DNMT1 |  |
|  |  |  |  | CCDC6 | DNMT3A |  |
|  |  |  |  | CCDC88C | DTX1 |  |
|  |  |  |  | CCK | DTX3 |  |
|  |  |  |  | CCL2 | DTX4 |  |
|  |  |  |  | CCNA2 | DUSP10 |  |
|  |  |  |  | CCNB1IP1 | DUSP2 |  |
|  |  |  |  | CCNB3 | DUSP4 |  |
|  |  |  |  | CCND1 | DUSP5 |  |
|  |  |  |  | CCND2 | DUSP6 |  |
|  |  |  |  | CCND3 | DUSP8 |  |
|  |  |  |  | CCNE1 | E2F1 |  |
|  |  |  |  | CCNG1 | E2F5 |  |
|  |  |  |  | CCT6B | EFNA1 |  |
|  |  |  |  | CD19 | EFNA2 |  |
|  |  |  |  | CD22 | EFNA3 |  |
|  |  |  |  | CD274 | EFNA5 |  |
|  |  |  |  | CD28 | EGF |  |
|  |  |  |  | CD36 | EGFR |  |
|  |  |  |  | CD44 | EIF4EBP1 |  |
|  |  |  |  | CD58 | ENDOG |  |
|  |  |  |  | CD70 | EP300 |  |
|  |  |  |  | CD74 | EPHA2 |  |
|  |  |  |  | CD79A | EPO |  |
|  |  |  |  | CD79B | EPOR |  |
|  |  |  |  | CD8A | ERBB2 |  |
|  |  |  |  | CDC14A | ERCC2 |  |
|  |  |  |  | CDC14B | ERCC6 |  |
|  |  |  |  | CDC25A | ETS2 |  |
|  |  |  |  | CDC25C | ETV1 |  |
|  |  |  |  | CDC42 | ETV4 |  |
|  |  |  |  | CDC73 | ETV7 |  |
|  |  |  |  | CDH1 | EYA1 |  |
|  |  |  |  | CDH11 | EZH2 |  |
|  |  |  |  | CDK1 | FANCA |  |
|  |  |  |  | CDK12 | FANCB |  |
|  |  |  |  | CDK2 | FANCC |  |
|  |  |  |  | CDK4 | FANCE |  |
|  |  |  |  | CDK5RAP2 | FANCF |  |
|  |  |  |  | CDK6 | FANCG |  |
|  |  |  |  | CDK7 | FANCL |  |
|  |  |  |  | CDK8 | FAS |  |
|  |  |  |  | CDK9 | FASLG |  |
|  |  |  |  | CDKL5 | FBXW7 |  |
|  |  |  |  | CDKN1A | FEN1 |  |
|  |  |  |  | CDKN1B | FGF1 |  |
|  |  |  |  | CDKN1C | FGF10 |  |
|  |  |  |  | CDKN2A | FGF11 |  |
|  |  |  |  | CDKN2B | FGF12 |  |
|  |  |  |  | CDKN2C | FGF13 |  |
|  |  |  |  | CDKN2D | FGF14 |  |
|  |  |  |  | CDX1 | FGF16 |  |
|  |  |  |  | CDX2 | FGF17 |  |
|  |  |  |  | CEBPA | FGF18 |  |
|  |  |  |  | CEBPB | FGF19 |  |
|  |  |  |  | CEBPD | FGF2 |  |
|  |  |  |  | CEBPE | FGF20 |  |
|  |  |  |  | CENPF | FGF21 |  |
|  |  |  |  | CENPU | FGF22 |  |
|  |  |  |  | CEP170B | FGF23 |  |
|  |  |  |  | CEP57 | FGF3 |  |
|  |  |  |  | CEP85L | FGF4 |  |
|  |  |  |  | CHCHD7 | FGF5 |  |
|  |  |  |  | CHD2 | FGF6 |  |
|  |  |  |  | CHD6 | FGF7 |  |
|  |  |  |  | CHEK1 | FGF8 |  |
|  |  |  |  | CHEK2 | FGF9 |  |
|  |  |  |  | CHIC2 | FGFR1 |  |
|  |  |  |  | CHL1 | FGFR2 |  |
|  |  |  |  | CHMP2B | FGFR3 |  |
|  |  |  |  | CHN1 | FGFR4 |  |
|  |  |  |  | CHST11 | FIGF |  |
|  |  |  |  | CHUK | FLNA |  |
|  |  |  |  | CIC | FLNC |  |
|  |  |  |  | CIITA | FLT1 |  |
|  |  |  |  | CIRH1A | FLT3 |  |
|  |  |  |  | CIT | FN1 |  |
|  |  |  |  | CKB | FOS |  |
|  |  |  |  | CKS1B | FOSL1 |  |
|  |  |  |  | CLP1 | FOXL2 |  |
|  |  |  |  | CLTA | FOXO4 |  |
|  |  |  |  | CLTC | FST |  |
|  |  |  |  | CLTCL1 | FUBP1 |  |
|  |  |  |  | CMKLR1 | FUT8 |  |
|  |  |  |  | CNBP | FZD10 |  |
|  |  |  |  | CNOT2 | FZD2 |  |
|  |  |  |  | CNTN1 | FZD3 |  |
|  |  |  |  | CNTRL | FZD7 |  |
|  |  |  |  | COG5 | FZD8 |  |
|  |  |  |  | COL11A1 | FZD9 |  |
|  |  |  |  | COL1A1 | GADD45A |  |
|  |  |  |  | COL1A2 | GADD45B |  |
|  |  |  |  | COL3A1 | GADD45G |  |
|  |  |  |  | COL6A3 | GAS1 |  |
|  |  |  |  | COL9A3 | GATA1 |  |
|  |  |  |  | COMMD1 | GATA2 |  |
|  |  |  |  | COX6C | GATA3 |  |
|  |  |  |  | CPNE1 | GDF6 |  |
|  |  |  |  | CPS1 | GHR |  |
|  |  |  |  | CPSF6 | GLI1 |  |
|  |  |  |  | CRADD | GLI3 |  |
|  |  |  |  | CREB1 | GNA11 |  |
|  |  |  |  | CREB3L1 | GNAQ |  |
|  |  |  |  | CREB3L2 | GNAS |  |
|  |  |  |  | CREBBP | GNG12 |  |
|  |  |  |  | CRKL | GNG4 |  |
|  |  |  |  | CRLF2 | GNG7 |  |
|  |  |  |  | CRTC1 | GNGT1 |  |
|  |  |  |  | CRTC3 | GPC4 |  |
|  |  |  |  | CSF1 | GRB2 |  |
|  |  |  |  | CSF1R | GRIA3 |  |
|  |  |  |  | CSF3 | GRIN1 |  |
|  |  |  |  | CSF3R | GRIN2A |  |
|  |  |  |  | CSNK1G2 | GRIN2B |  |
|  |  |  |  | CSNK2A1 | GSK3B |  |
|  |  |  |  | CTCF | GTF2H3 |  |
|  |  |  |  | CTDSP2 | GZMB |  |
|  |  |  |  | CTLA4 | H2AFX |  |
|  |  |  |  | CTNNA1 | H3F3A |  |
|  |  |  |  | CTNNB1 | H3F3C |  |
|  |  |  |  | CTNND2 | HDAC1 |  |
|  |  |  |  | CTRB1 | HDAC10 |  |
|  |  |  |  | CTSA | HDAC11 |  |
|  |  |  |  | CUX1 | HDAC2 |  |
|  |  |  |  | CXCL8 | HDAC4 |  |
|  |  |  |  | CXCR4 | HDAC5 |  |
|  |  |  |  | CXXC4 | HDAC6 |  |
|  |  |  |  | CYFIP2 | HELLS |  |
|  |  |  |  | CYLD | HES1 |  |
|  |  |  |  | CYP1B1 | HES5 |  |
|  |  |  |  | CYP2C19 | HGF |  |
|  |  |  |  | DAB2IP | HHEX |  |
|  |  |  |  | DACH1 | HHIP |  |
|  |  |  |  | DACH2 | HIST1H3B |  |
|  |  |  |  | DAXX | HIST1H3G |  |
|  |  |  |  | DCLK2 | HIST1H3H |  |
|  |  |  |  | DCN | HMGA1 |  |
|  |  |  |  | DDB2 | HMGA2 |  |
|  |  |  |  | DDIT3 | HNF1A |  |
|  |  |  |  | DDR2 | HOXA10 |  |
|  |  |  |  | DDX10 | HOXA11 |  |
|  |  |  |  | DDX20 | HOXA9 |  |
|  |  |  |  | DDX39B | HPGD |  |
|  |  |  |  | DDX3X | HRAS |  |
|  |  |  |  | DDX5 | HSP90B1 |  |
|  |  |  |  | DDX6 | HSPA1A |  |
|  |  |  |  | DEK | HSPA2 |  |
|  |  |  |  | DGKB | HSPA6 |  |
|  |  |  |  | DGKI | HSPB1 |  |
|  |  |  |  | DGKZ | IBSP |  |
|  |  |  |  | DICER1 | ID1 |  |
|  |  |  |  | DIRAS3 | ID2 |  |
|  |  |  |  | DIS3L2 | ID4 |  |
|  |  |  |  | DKK1 | IDH1 |  |
|  |  |  |  | DKK2 | IDH2 |  |
|  |  |  |  | DKK4 | IFNA17 |  |
|  |  |  |  | DLEC1 | IFNA2 |  |
|  |  |  |  | DLL1 | IFNA7 |  |
|  |  |  |  | DLL3 | IFNG |  |
|  |  |  |  | DLL4 | IGF1 |  |
|  |  |  |  | DMRT1 | IGF1R |  |
|  |  |  |  | DMRTA2 | IGFBP3 |  |
|  |  |  |  | DNAJB1 | IKBKB |  |
|  |  |  |  | DNM1 | IKBKG |  |
|  |  |  |  | DNM2 | IL10 |  |
|  |  |  |  | DNM3 | IL11 |  |
|  |  |  |  | DNMT1 | IL11RA |  |
|  |  |  |  | DNMT3A | IL12A |  |
|  |  |  |  | DOCK1 | IL12B |  |
|  |  |  |  | DOT1L | IL12RB2 |  |
|  |  |  |  | DPM1 | IL13 |  |
|  |  |  |  | DPYD | IL13RA2 |  |
|  |  |  |  | DST | IL15 |  |
|  |  |  |  | DTX1 | IL19 |  |
|  |  |  |  | DTX4 | IL1A |  |
|  |  |  |  | DUSP2 | IL1B |  |
|  |  |  |  | DUSP22 | IL1R1 |  |
|  |  |  |  | DUSP26 | IL1R2 |  |
|  |  |  |  | DUSP9 | IL1RAP |  |
|  |  |  |  | DUX4 | IL20RA |  |
|  |  |  |  | E2F1 | IL20RB |  |
|  |  |  |  | EBF1 | IL22RA1 |  |
|  |  |  |  | ECT2L | IL22RA2 |  |
|  |  |  |  | EDIL3 | IL23A |  |
|  |  |  |  | EDNRB | IL23R |  |
|  |  |  |  | EED | IL24 |  |
|  |  |  |  | EEFSEC | IL2RA |  |
|  |  |  |  | EGF | IL2RB |  |
|  |  |  |  | EGFR | IL3 |  |
|  |  |  |  | EGR1 | IL3RA |  |
|  |  |  |  | EGR2 | IL5RA |  |
|  |  |  |  | EGR3 | IL6 |  |
|  |  |  |  | EGR4 | IL6R |  |
|  |  |  |  | EIF4A2 | IL7 |  |
|  |  |  |  | EIF4E | IL7R |  |
|  |  |  |  | ELF4 | IL8 |  |
|  |  |  |  | ELK4 | INHBA |  |
|  |  |  |  | ELL | INHBB |  |
|  |  |  |  | ELN | IRAK2 |  |
|  |  |  |  | ELOVL2 | IRAK3 |  |
|  |  |  |  | ELP2 | IRS1 |  |
|  |  |  |  | EML1 | ITGA2 |  |
|  |  |  |  | EML4 | ITGA3 |  |
|  |  |  |  | ENPP2 | ITGA6 |  |
|  |  |  |  | EP300 | ITGA7 |  |
|  |  |  |  | EP400 | ITGA8 |  |
|  |  |  |  | EPC1 | ITGA9 |  |
|  |  |  |  | EPCAM | ITGB3 |  |
|  |  |  |  | EPHA10 | ITGB4 |  |
|  |  |  |  | EPHA2 | ITGB6 |  |
|  |  |  |  | EPHA3 | ITGB7 |  |
|  |  |  |  | EPHA5 | ITGB8 |  |
|  |  |  |  | EPHA7 | JAG1 |  |
|  |  |  |  | EPHB1 | JAG2 |  |
|  |  |  |  | EPHB6 | JAK1 |  |
|  |  |  |  | EPO | JAK2 |  |
|  |  |  |  | EPOR | JAK3 |  |
|  |  |  |  | EPS15 | JUN |  |
|  |  |  |  | ERBB2 | KAT2B |  |
|  |  |  |  | ERBB3 | KDM5C |  |
|  |  |  |  | ERBB4 | KDM6A |  |
|  |  |  |  | ERC1 | KIT |  |
|  |  |  |  | ERCC1 | KITLG |  |
|  |  |  |  | ERCC2 | KLF4 |  |
|  |  |  |  | ERCC3 | KMT2C |  |
|  |  |  |  | ERCC4 | KMT2D |  |
|  |  |  |  | ERCC5 | KRAS |  |
|  |  |  |  | ERCC6 | LAMA1 |  |
|  |  |  |  | ERG | LAMA3 |  |
|  |  |  |  | ERLIN2 | LAMA5 |  |
|  |  |  |  | ESR1 | LAMB3 |  |
|  |  |  |  | ETS1 | LAMB4 |  |
|  |  |  |  | ETS2 | LAMC2 |  |
|  |  |  |  | ETV1 | LAMC3 |  |
|  |  |  |  | ETV4 | LAT |  |
|  |  |  |  | ETV5 | LEF1 |  |
|  |  |  |  | ETV6 | LEFTY1 |  |
|  |  |  |  | EWSR1 | LEFTY2 |  |
|  |  |  |  | EXOSC6 | LEP |  |
|  |  |  |  | EXT1 | LEPR |  |
|  |  |  |  | EXT2 | LFNG |  |
|  |  |  |  | EYA1 | LIF |  |
|  |  |  |  | EYA2 | LIFR |  |
|  |  |  |  | EZH2 | LIG4 |  |
|  |  |  |  | EZR | LRP2 |  |
|  |  |  |  | FAF1 | LTBP1 |  |
|  |  |  |  | FAM127C | MAD2L2 |  |
|  |  |  |  | FAM19A2 | MAML2 |  |
|  |  |  |  | FAM19A5 | MAP2K1 |  |
|  |  |  |  | FAM46C | MAP2K2 |  |
|  |  |  |  | FAM64A | MAP2K4 |  |
|  |  |  |  | FANCA | MAP2K6 |  |
|  |  |  |  | FANCB | MAP3K1 |  |
|  |  |  |  | FANCC | MAP3K12 |  |
|  |  |  |  | FANCD2 | MAP3K13 |  |
|  |  |  |  | FANCE | MAP3K14 |  |
|  |  |  |  | FANCF | MAP3K5 |  |
|  |  |  |  | FANCG | MAP3K8 |  |
|  |  |  |  | FANCI | MAPK1 |  |
|  |  |  |  | FANCL | MAPK10 |  |
|  |  |  |  | FANCM | MAPK12 |  |
|  |  |  |  | FAS | MAPK3 |  |
|  |  |  |  | FASLG | MAPK8 |  |
|  |  |  |  | FBN2 | MAPK8IP1 |  |
|  |  |  |  | FBXO11 | MAPK8IP2 |  |
|  |  |  |  | FBXO31 | MAPK9 |  |
|  |  |  |  | FBXW7 | MAPT |  |
|  |  |  |  | FCGBP | MCM2 |  |
|  |  |  |  | FCGR2B | MCM4 |  |
|  |  |  |  | FCRL4 | MCM5 |  |
|  |  |  |  | FEN1 | MCM7 |  |
|  |  |  |  | FEV | MDC1 |  |
|  |  |  |  | FGF1 | MDM2 |  |
|  |  |  |  | FGF10 | MECOM |  |
|  |  |  |  | FGF13 | MED12 |  |
|  |  |  |  | FGF14 | MEN1 |  |
|  |  |  |  | FGF19 | MET |  |
|  |  |  |  | FGF2 | MFNG |  |
|  |  |  |  | FGF23 | MGMT |  |
|  |  |  |  | FGF3 | MLF1 |  |
|  |  |  |  | FGF4 | MLH1 |  |
|  |  |  |  | FGF6 | MLLT3 |  |
|  |  |  |  | FGF8 | MLLT4 |  |
|  |  |  |  | FGF9 | MMP3 |  |
|  |  |  |  | FGFR1 | MMP7 |  |
|  |  |  |  | FGFR1OP | MMP9 |  |
|  |  |  |  | FGFR1OP2 | MNAT1 |  |
|  |  |  |  | FGFR2 | MPL |  |
|  |  |  |  | FGFR3 | MPO |  |
|  |  |  |  | FGFR4 | MSH2 |  |
|  |  |  |  | FH | MSH6 |  |
|  |  |  |  | FHIT | MTOR |  |
|  |  |  |  | FHL2 | MUTYH |  |
|  |  |  |  | FIGF | MYB |  |
|  |  |  |  | FIP1L1 | MYC |  |
|  |  |  |  | FLCN | MYCN |  |
|  |  |  |  | FLI1 | MYD88 |  |
|  |  |  |  | FLNA | NASP |  |
|  |  |  |  | FLNC | NBN |  |
|  |  |  |  | FLT1 | NCOR1 |  |
|  |  |  |  | FLT3 | NF1 |  |
|  |  |  |  | FLT3LG | NF2 |  |
|  |  |  |  | FLT4 | NFATC1 |  |
|  |  |  |  | FLYWCH1 | NFE2L2 |  |
|  |  |  |  | FNBP1 | NFKB1 |  |
|  |  |  |  | FOS | NFKBIA |  |
|  |  |  |  | FOSB | NFKBIZ |  |
|  |  |  |  | FOSL1 | NGF |  |
|  |  |  |  | FOXL2 | NGFR |  |
|  |  |  |  | FOXO1 | NKD1 |  |
|  |  |  |  | FOXO3 | NODAL |  |
|  |  |  |  | FOXO4 | NOG |  |
|  |  |  |  | FOXP1 | NOS3 |  |
|  |  |  |  | FRK | NOTCH1 |  |
|  |  |  |  | FRMPD4 | NOTCH2 |  |
|  |  |  |  | FRS2 | NOTCH3 |  |
|  |  |  |  | FRYL | NPM1 |  |
|  |  |  |  | FSTL3 | NPM2 |  |
|  |  |  |  | FUS | NR4A1 |  |
|  |  |  |  | FUT1 | NR4A3 |  |
|  |  |  |  | FZD10 | NRAS |  |
|  |  |  |  | FZD2 | NSD1 |  |
|  |  |  |  | FZD3 | NTF3 |  |
|  |  |  |  | FZD6 | NTHL1 |  |
|  |  |  |  | FZD7 | NTRK1 |  |
|  |  |  |  | FZD8 | NTRK2 |  |
|  |  |  |  | GAB1 | NUMBL |  |
|  |  |  |  | GABRG2 | NUPR1 |  |
|  |  |  |  | GADD45B | OSM |  |
|  |  |  |  | GANAB | PAK3 |  |
|  |  |  |  | GAS1 | PAK7 |  |
|  |  |  |  | GAS5 | PAX3 |  |
|  |  |  |  | GAS7 | PAX5 |  |
|  |  |  |  | GATA1 | PAX8 |  |
|  |  |  |  | GATA2 | PBRM1 |  |
|  |  |  |  | GATA3 | PBX1 |  |
|  |  |  |  | GATA6 | PBX3 |  |
|  |  |  |  | GBP2 | PCK1 |  |
|  |  |  |  | GDF6 | PCNA |  |
|  |  |  |  | GFAP | PDGFA |  |
|  |  |  |  | GHR | PDGFB |  |
|  |  |  |  | GID4 | PDGFC |  |
|  |  |  |  | GIT2 | PDGFD |  |
|  |  |  |  | GLI1 | PDGFRA |  |
|  |  |  |  | GLI3 | PDGFRB |  |
|  |  |  |  | GMPS | PGF |  |
|  |  |  |  | GNA11 | PHF6 |  |
|  |  |  |  | GNA12 | PIK3CA |  |
|  |  |  |  | GNA13 | PIK3CB |  |
|  |  |  |  | GNAI1 | PIK3CD |  |
|  |  |  |  | GNAQ | PIK3CG |  |
|  |  |  |  | GNAS | PIK3R1 |  |
|  |  |  |  | GNG4 | PIK3R2 |  |
|  |  |  |  | GOLGA5 | PIK3R3 |  |
|  |  |  |  | GOPC | PIK3R5 |  |
|  |  |  |  | GOSR1 | PIM1 |  |
|  |  |  |  | GOT1 | PITX2 |  |
|  |  |  |  | GPC3 | PKMYT1 |  |
|  |  |  |  | GPHN | PLA1A |  |
|  |  |  |  | GPR124 | PLA2G10 |  |
|  |  |  |  | GPR128 | PLA2G2A |  |
|  |  |  |  | GPR34 | PLA2G3 |  |
|  |  |  |  | GRB10 | PLA2G4A |  |
|  |  |  |  | GRB2 | PLA2G4C |  |
|  |  |  |  | GRHPR | PLA2G4E |  |
|  |  |  |  | GRID1 | PLA2G4F |  |
|  |  |  |  | GRIN2A | PLA2G5 |  |
|  |  |  |  | GRIN2B | PLAT |  |
|  |  |  |  | GRM1 | PLAU |  |
|  |  |  |  | GRM3 | PLCB1 |  |
|  |  |  |  | GSK3B | PLCB4 |  |
|  |  |  |  | GSN | PLCE1 |  |
|  |  |  |  | GSTT1 | PLCG2 |  |
|  |  |  |  | GTF2I | PLD1 |  |
|  |  |  |  | GTSE1 | PML |  |
|  |  |  |  | H2AFX | POLB |  |
|  |  |  |  | H3F3A | POLD1 |  |
|  |  |  |  | HAS2 | POLD4 |  |
|  |  |  |  | HDAC1 | POLE2 |  |
|  |  |  |  | HDAC2 | POLR2D |  |
|  |  |  |  | HDAC3 | POLR2H |  |
|  |  |  |  | HDAC4 | POLR2J |  |
|  |  |  |  | HDAC5 | PPARG |  |
|  |  |  |  | HDAC6 | PPARGC1A |  |
|  |  |  |  | HDAC7 | PPP2CB |  |
|  |  |  |  | HECW1 | PPP2R1A |  |
|  |  |  |  | HEPH | PPP2R2B |  |
|  |  |  |  | HERPUD1 | PPP2R2C |  |
|  |  |  |  | HES1 | PPP3CA |  |
|  |  |  |  | HES5 | PPP3CB |  |
|  |  |  |  | HEY1 | PPP3CC |  |
|  |  |  |  | HGF | PPP3R1 |  |
|  |  |  |  | HHEX | PPP3R2 |  |
|  |  |  |  | HIF1A | PRDM1 |  |
|  |  |  |  | HIP1 | PRKAA2 |  |
|  |  |  |  | HIPK1 | PRKACA |  |
|  |  |  |  | HIPK2 | PRKACB |  |
|  |  |  |  | HIST1H1C | PRKACG |  |
|  |  |  |  | HIST1H1D | PRKAR1B |  |
|  |  |  |  | HIST1H1E | PRKAR2A |  |
|  |  |  |  | HIST1H2AC | PRKAR2B |  |
|  |  |  |  | HIST1H2AG | PRKCA |  |
|  |  |  |  | HIST1H2AL | PRKCB |  |
|  |  |  |  | HIST1H2AM | PRKCG |  |
|  |  |  |  | HIST1H2BC | PRKDC |  |
|  |  |  |  | HIST1H2BJ | PRKX |  |
|  |  |  |  | HIST1H2BK | PRL |  |
|  |  |  |  | HIST1H2BO | PRLR |  |
|  |  |  |  | HIST1H3B | PRMT8 |  |
|  |  |  |  | HIST1H4I | PROM1 |  |
|  |  |  |  | HLF | PTCH1 |  |
|  |  |  |  | HMGA1 | PTCRA |  |
|  |  |  |  | HMGA2 | PTEN |  |
|  |  |  |  | HMGB1 | PTPN11 |  |
|  |  |  |  | HMGN2P46 | PTPN5 |  |
|  |  |  |  | HNF1A | PTPRR |  |
|  |  |  |  | HNRNPA2B1 | PTTG2 |  |
|  |  |  |  | HOOK3 | RAC1 |  |
|  |  |  |  | HOXA10 | RAC2 |  |
|  |  |  |  | HOXA11 | RAC3 |  |
|  |  |  |  | HOXA13 | RAD21 |  |
|  |  |  |  | HOXA3 | RAD50 |  |
|  |  |  |  | HOXA9 | RAD51 |  |
|  |  |  |  | HOXC11 | RAD52 |  |
|  |  |  |  | HOXC13 | RAF1 |  |
|  |  |  |  | HOXD11 | RASA4 |  |
|  |  |  |  | HOXD13 | RASAL1 |  |
|  |  |  |  | HOXD9 | RASGRF1 |  |
|  |  |  |  | HRAS | RASGRF2 |  |
|  |  |  |  | HSP90AA1 | RASGRP1 |  |
|  |  |  |  | HSP90AB1 | RASGRP2 |  |
|  |  |  |  | HSPA1A | RB1 |  |
|  |  |  |  | HSPA2 | RBX1 |  |
|  |  |  |  | HSPA4 | RELA |  |
|  |  |  |  | HSPA5 | RELN |  |
|  |  |  |  | HTRA1 | RET |  |
|  |  |  |  | HUWE1 | RFC3 |  |
|  |  |  |  | IBSP | RFC4 |  |
|  |  |  |  | ICAM1 | RHOA |  |
|  |  |  |  | ICK | RIN1 |  |
|  |  |  |  | ID1 | RNF43 |  |
|  |  |  |  | ID3 | RPA3 |  |
|  |  |  |  | ID4 | RPS27A |  |
|  |  |  |  | IDH1 | RPS6KA5 |  |
|  |  |  |  | IDH2 | RPS6KA6 |  |
|  |  |  |  | IFNG | RRAS2 |  |
|  |  |  |  | IFRD1 | RUNX1 |  |
|  |  |  |  | IGF1 | RUNX1T1 |  |
|  |  |  |  | IGF1R | RXRG |  |
|  |  |  |  | IGFBP2 | SETBP1 |  |
|  |  |  |  | IGFBP3 | SETD2 |  |
|  |  |  |  | IKBKB | SF3B1 |  |
|  |  |  |  | IKBKE | SFN |  |
|  |  |  |  | IKZF1 | SFRP1 |  |
|  |  |  |  | IKZF2 | SFRP2 |  |
|  |  |  |  | IKZF3 | SFRP4 |  |
|  |  |  |  | IL12RB2 | SGK2 |  |
|  |  |  |  | IL13 | SHC1 |  |
|  |  |  |  | IL13RA2 | SHC2 |  |
|  |  |  |  | IL15 | SHC3 |  |
|  |  |  |  | IL1B | SHC4 |  |
|  |  |  |  | IL1R1 | SIN3A |  |
|  |  |  |  | IL1RAP | SIRT4 |  |
|  |  |  |  | IL2 | SIX1 |  |
|  |  |  |  | IL21R | SKP1 |  |
|  |  |  |  | IL2RA | SKP2 |  |
|  |  |  |  | IL3 | SMAD2 |  |
|  |  |  |  | IL6 | SMAD3 |  |
|  |  |  |  | IL7R | SMAD4 |  |
|  |  |  |  | INHBA | SMAD9 |  |
|  |  |  |  | INPP4A | SMARCA4 |  |
|  |  |  |  | INPP4B | SMARCB1 |  |
|  |  |  |  | INPP5A | SMC1A |  |
|  |  |  |  | INPP5D | SMC1B |  |
|  |  |  |  | IQCG | SMC3 |  |
|  |  |  |  | IRF1 | SMO |  |
|  |  |  |  | IRF2BP2 | SOCS1 |  |
|  |  |  |  | IRF4 | SOCS2 |  |
|  |  |  |  | IRF8 | SOCS3 |  |
|  |  |  |  | IRS1 | SOS1 |  |
|  |  |  |  | IRS2 | SOS2 |  |
|  |  |  |  | IRS4 | SOST |  |
|  |  |  |  | ITGA5 | SOX17 |  |
|  |  |  |  | ITGA7 | SOX9 |  |
|  |  |  |  | ITGA8 | SP1 |  |
|  |  |  |  | ITGAV | SPOP |  |
|  |  |  |  | ITGB3 | SPP1 |  |
|  |  |  |  | ITK | SPRY1 |  |
|  |  |  |  | ITPKA | SPRY2 |  |
|  |  |  |  | JAG2 | SPRY4 |  |
|  |  |  |  | JAK1 | SRSF2 |  |
|  |  |  |  | JAK2 | SSX1 |  |
|  |  |  |  | JAK3 | STAG2 |  |
|  |  |  |  | JARID2 | STAT1 |  |
|  |  |  |  | JAZF1 | STAT3 |  |
|  |  |  |  | JUN | STAT4 |  |
|  |  |  |  | KALRN | STK11 |  |
|  |  |  |  | KANK1 | STMN1 |  |
|  |  |  |  | KAT2B | SUV39H2 |  |
|  |  |  |  | KAT6A | SYK |  |
|  |  |  |  | KAT6B | TBL1XR1 |  |
|  |  |  |  | KCNB1 | TCF3 |  |
|  |  |  |  | KDM1A | TCF7L1 |  |
|  |  |  |  | KDM2B | TCL1B |  |
|  |  |  |  | KDM4C | TET2 |  |
|  |  |  |  | KDM5A | TFDP1 |  |
|  |  |  |  | KDM5C | TGFB1 |  |
|  |  |  |  | KDM6A | TGFB2 |  |
|  |  |  |  | KDR | TGFB3 |  |
|  |  |  |  | KDSR | TGFBR2 |  |
|  |  |  |  | KEAP1 | THBS1 |  |
|  |  |  |  | KIAA0232 | THBS4 |  |
|  |  |  |  | KIAA1524 | THEM4 |  |
|  |  |  |  | KIAA1549 | TIAM1 |  |
|  |  |  |  | KIAA1598 | TLR2 |  |
|  |  |  |  | KIF5B | TLR4 |  |
|  |  |  |  | KIT | TLX1 |  |
|  |  |  |  | KLF4 | TMPRSS2 |  |
|  |  |  |  | KLHL6 | TNC |  |
|  |  |  |  | KLK2 | TNF |  |
|  |  |  |  | KLK7 | TNFAIP3 |  |
|  |  |  |  | KMT2A | TNFRSF10A |  |
|  |  |  |  | KMT2B | TNFRSF10B |  |
|  |  |  |  | KMT2C | TNFRSF10C |  |
|  |  |  |  | KMT2D | TNFRSF10D |  |
|  |  |  |  | KPNB1 | TNFSF10 |  |
|  |  |  |  | KRAS | TNN |  |
|  |  |  |  | KSR1 | TNR |  |
|  |  |  |  | KTN1 | TP53 |  |
|  |  |  |  | LAMA1 | TPO |  |
|  |  |  |  | LAMA5 | TRAF7 |  |
|  |  |  |  | LAMP2 | TSC1 |  |
|  |  |  |  | LASP1 | TSHR |  |
|  |  |  |  | LCK | TSLP |  |
|  |  |  |  | LCP1 | TSPAN7 |  |
|  |  |  |  | LEF1 | TTK |  |
|  |  |  |  | LEFTY2 | U2AF1 |  |
|  |  |  |  | LFNG | UBB |  |
|  |  |  |  | LGALS3 | UBE2T |  |
|  |  |  |  | LGR5 | UTY |  |
|  |  |  |  | LHFP | VEGFA |  |
|  |  |  |  | LHX2 | VEGFC |  |
|  |  |  |  | LHX4 | VHL |  |
|  |  |  |  | LIFR | WEE1 |  |
|  |  |  |  | LINC00598 | WHSC1 |  |
|  |  |  |  | LINC00982 | WHSC1L1 |  |
|  |  |  |  | LINGO2 | WIF1 |  |
|  |  |  |  | LMBRD1 | WNT10A |  |
|  |  |  |  | LMO1 | WNT10B |  |
|  |  |  |  | LMO2 | WNT11 |  |
|  |  |  |  | LMO7 | WNT16 |  |
|  |  |  |  | LNP1 | WNT2 |  |
|  |  |  |  | LOX | WNT2B |  |
|  |  |  |  | LPAR1 | WNT3 |  |
|  |  |  |  | LPP | WNT4 |  |
|  |  |  |  | LPXN | WNT5A |  |
|  |  |  |  | LRIG3 | WNT5B |  |
|  |  |  |  | LRMP | WNT6 |  |
|  |  |  |  | LRP1B | WNT7A |  |
|  |  |  |  | LRP5 | WNT7B |  |
|  |  |  |  | LRPPRC | WT1 |  |
|  |  |  |  | LRRC37B | XPA |  |
|  |  |  |  | LRRC59 | XRCC4 |  |
|  |  |  |  | LRRC7 | ZAK |  |
|  |  |  |  | LRRK2 | ZBTB16 |  |
|  |  |  |  | LTBP1 | ZBTB32 |  |
|  |  |  |  | LYL1 | ZIC2 |  |
|  |  |  |  | LYN |  |  |
|  |  |  |  | MACROD1 |  |  |
|  |  |  |  | MAD2L1 |  |  |
|  |  |  |  | MADD |  |  |
|  |  |  |  | MAF |  |  |
|  |  |  |  | MAFB |  |  |
|  |  |  |  | MAGED1 |  |  |
|  |  |  |  | MAGEE1 |  |  |
|  |  |  |  | MALAT1 |  |  |
|  |  |  |  | MALT1 |  |  |
|  |  |  |  | MAML1 |  |  |
|  |  |  |  | MAML2 |  |  |
|  |  |  |  | MAP2 |  |  |
|  |  |  |  | MAP2K1 |  |  |
|  |  |  |  | MAP2K2 |  |  |
|  |  |  |  | MAP2K3 |  |  |
|  |  |  |  | MAP2K4 |  |  |
|  |  |  |  | MAP2K5 |  |  |
|  |  |  |  | MAP2K6 |  |  |
|  |  |  |  | MAP2K7 |  |  |
|  |  |  |  | MAP3K1 |  |  |
|  |  |  |  | MAP3K14 |  |  |
|  |  |  |  | MAP3K6 |  |  |
|  |  |  |  | MAP3K7 |  |  |
|  |  |  |  | MAPK1 |  |  |
|  |  |  |  | MAPK3 |  |  |
|  |  |  |  | MAPK8 |  |  |
|  |  |  |  | MAPK8IP2 |  |  |
|  |  |  |  | MAPK9 |  |  |
|  |  |  |  | MAPRE1 |  |  |
|  |  |  |  | MATK |  |  |
|  |  |  |  | MAX |  |  |
|  |  |  |  | MB21D2 |  |  |
|  |  |  |  | MBNL1 |  |  |
|  |  |  |  | MBTD1 |  |  |
|  |  |  |  | MCL1 |  |  |
|  |  |  |  | MDC1 |  |  |
|  |  |  |  | MDH1 |  |  |
|  |  |  |  | MDM2 |  |  |
|  |  |  |  | MDM4 |  |  |
|  |  |  |  | MDS2 |  |  |
|  |  |  |  | MEAF6 |  |  |
|  |  |  |  | MECOM |  |  |
|  |  |  |  | MED12 |  |  |
|  |  |  |  | MEF2B |  |  |
|  |  |  |  | MEF2C |  |  |
|  |  |  |  | MEF2D |  |  |
|  |  |  |  | MELK |  |  |
|  |  |  |  | MEN1 |  |  |
|  |  |  |  | MET |  |  |
|  |  |  |  | METTL18 |  |  |
|  |  |  |  | METTL7B |  |  |
|  |  |  |  | MFNG |  |  |
|  |  |  |  | MGEA5 |  |  |
|  |  |  |  | MGMT |  |  |
|  |  |  |  | MIB1 |  |  |
|  |  |  |  | MIPOL1 |  |  |
|  |  |  |  | MITF |  |  |
|  |  |  |  | MKI67 |  |  |
|  |  |  |  | MKL1 |  |  |
|  |  |  |  | MKL2 |  |  |
|  |  |  |  | MLF1 |  |  |
|  |  |  |  | MLH1 |  |  |
|  |  |  |  | MLLT1 |  |  |
|  |  |  |  | MLLT10 |  |  |
|  |  |  |  | MLLT11 |  |  |
|  |  |  |  | MLLT3 |  |  |
|  |  |  |  | MLLT4 |  |  |
|  |  |  |  | MLLT6 |  |  |
|  |  |  |  | MMP7 |  |  |
|  |  |  |  | MMP9 |  |  |
|  |  |  |  | MN1 |  |  |
|  |  |  |  | MNAT1 |  |  |
|  |  |  |  | MNX1 |  |  |
|  |  |  |  | MPL |  |  |
|  |  |  |  | MRE11A |  |  |
|  |  |  |  | MSH2 |  |  |
|  |  |  |  | MSH3 |  |  |
|  |  |  |  | MSH6 |  |  |
|  |  |  |  | MSI2 |  |  |
|  |  |  |  | MSN |  |  |
|  |  |  |  | MTCP1 |  |  |
|  |  |  |  | MTOR |  |  |
|  |  |  |  | MTUS2 |  |  |
|  |  |  |  | MUC1 |  |  |
|  |  |  |  | MUTYH |  |  |
|  |  |  |  | MYB |  |  |
|  |  |  |  | MYBL1 |  |  |
|  |  |  |  | MYC |  |  |
|  |  |  |  | MYCL |  |  |
|  |  |  |  | MYCN |  |  |
|  |  |  |  | MYD88 |  |  |
|  |  |  |  | MYH11 |  |  |
|  |  |  |  | MYH9 |  |  |
|  |  |  |  | MYO18A |  |  |
|  |  |  |  | MYO1F |  |  |
|  |  |  |  | NAB2 |  |  |
|  |  |  |  | NACA |  |  |
|  |  |  |  | NAPA |  |  |
|  |  |  |  | NAV3 |  |  |
|  |  |  |  | NBEAP1 |  |  |
|  |  |  |  | NBN |  |  |
|  |  |  |  | NBR1 |  |  |
|  |  |  |  | NCAM1 |  |  |
|  |  |  |  | NCKIPSD |  |  |
|  |  |  |  | NCOA1 |  |  |
|  |  |  |  | NCOA2 |  |  |
|  |  |  |  | NCOA3 |  |  |
|  |  |  |  | NCOA4 |  |  |
|  |  |  |  | NCOR2 |  |  |
|  |  |  |  | NCSTN |  |  |
|  |  |  |  | NDC80 |  |  |
|  |  |  |  | NDE1 |  |  |
|  |  |  |  | NDRG1 |  |  |
|  |  |  |  | NDUFAF1 |  |  |
|  |  |  |  | NEDD4 |  |  |
|  |  |  |  | NEURL1 |  |  |
|  |  |  |  | NF1 |  |  |
|  |  |  |  | NF2 |  |  |
|  |  |  |  | NFATC1 |  |  |
|  |  |  |  | NFATC2 |  |  |
|  |  |  |  | NFE2L2 |  |  |
|  |  |  |  | NFIB |  |  |
|  |  |  |  | NFKB1 |  |  |
|  |  |  |  | NFKB2 |  |  |
|  |  |  |  | NFKBIA |  |  |
|  |  |  |  | NGF |  |  |
|  |  |  |  | NGFR |  |  |
|  |  |  |  | NIN |  |  |
|  |  |  |  | NIPBL |  |  |
|  |  |  |  | NKX2-1 |  |  |
|  |  |  |  | NKX2-5 |  |  |
|  |  |  |  | NOD1 |  |  |
|  |  |  |  | NODAL |  |  |
|  |  |  |  | NONO |  |  |
|  |  |  |  | NOS3 |  |  |
|  |  |  |  | NOTCH1 |  |  |
|  |  |  |  | NOTCH2 |  |  |
|  |  |  |  | NOTCH3 |  |  |
|  |  |  |  | NOTCH4 |  |  |
|  |  |  |  | NPM1 |  |  |
|  |  |  |  | NPM2 |  |  |
|  |  |  |  | NR3C1 |  |  |
|  |  |  |  | NR4A3 |  |  |
|  |  |  |  | NR6A1 |  |  |
|  |  |  |  | NRAS |  |  |
|  |  |  |  | NSD1 |  |  |
|  |  |  |  | NT5C2 |  |  |
|  |  |  |  | NTF3 |  |  |
|  |  |  |  | NTF4 |  |  |
|  |  |  |  | NTRK1 |  |  |
|  |  |  |  | NTRK2 |  |  |
|  |  |  |  | NTRK3 |  |  |
|  |  |  |  | NUMA1 |  |  |
|  |  |  |  | NUP107 |  |  |
|  |  |  |  | NUP214 |  |  |
|  |  |  |  | NUP93 |  |  |
|  |  |  |  | NUP98 |  |  |
|  |  |  |  | NUTM1 |  |  |
|  |  |  |  | NUTM2A |  |  |
|  |  |  |  | NUTM2B |  |  |
|  |  |  |  | OFD1 |  |  |
|  |  |  |  | OLIG1 |  |  |
|  |  |  |  | OLIG2 |  |  |
|  |  |  |  | OLR1 |  |  |
|  |  |  |  | OMD |  |  |
|  |  |  |  | P2RY8 |  |  |
|  |  |  |  | PAFAH1B2 |  |  |
|  |  |  |  | PAG1 |  |  |
|  |  |  |  | PAK1 |  |  |
|  |  |  |  | PAK3 |  |  |
|  |  |  |  | PAK6 |  |  |
|  |  |  |  | PAK7 |  |  |
|  |  |  |  | PALB2 |  |  |
|  |  |  |  | PAPPA |  |  |
|  |  |  |  | PASK |  |  |
|  |  |  |  | PATZ1 |  |  |
|  |  |  |  | PAX3 |  |  |
|  |  |  |  | PAX5 |  |  |
|  |  |  |  | PAX7 |  |  |
|  |  |  |  | PAX8 |  |  |
|  |  |  |  | PBRM1 |  |  |
|  |  |  |  | PBX1 |  |  |
|  |  |  |  | PC |  |  |
|  |  |  |  | PCBP1 |  |  |
|  |  |  |  | PCLO |  |  |
|  |  |  |  | PCM1 |  |  |
|  |  |  |  | PCNA |  |  |
|  |  |  |  | PCSK7 |  |  |
|  |  |  |  | PDCD1 |  |  |
|  |  |  |  | PDCD11 |  |  |
|  |  |  |  | PDCD1LG2 |  |  |
|  |  |  |  | PDE4DIP |  |  |
|  |  |  |  | PDGFA |  |  |
|  |  |  |  | PDGFB |  |  |
|  |  |  |  | PDGFD |  |  |
|  |  |  |  | PDGFRA |  |  |
|  |  |  |  | PDGFRB |  |  |
|  |  |  |  | PDK1 |  |  |
|  |  |  |  | PEG3 |  |  |
|  |  |  |  | PER1 |  |  |
|  |  |  |  | PFDN5 |  |  |
|  |  |  |  | PHB |  |  |
|  |  |  |  | PHF1 |  |  |
|  |  |  |  | PHF23 |  |  |
|  |  |  |  | PHF6 |  |  |
|  |  |  |  | PHOX2B |  |  |
|  |  |  |  | PI4KA |  |  |
|  |  |  |  | PICALM |  |  |
|  |  |  |  | PIK3CA |  |  |
|  |  |  |  | PIK3CB |  |  |
|  |  |  |  | PIK3CD |  |  |
|  |  |  |  | PIK3CG |  |  |
|  |  |  |  | PIK3R1 |  |  |
|  |  |  |  | PIK3R2 |  |  |
|  |  |  |  | PIM1 |  |  |
|  |  |  |  | PKM |  |  |
|  |  |  |  | PLA2G2A |  |  |
|  |  |  |  | PLA2G5 |  |  |
|  |  |  |  | PLAG1 |  |  |
|  |  |  |  | PLAT |  |  |
|  |  |  |  | PLAU |  |  |
|  |  |  |  | PLCB1 |  |  |
|  |  |  |  | PLCB4 |  |  |
|  |  |  |  | PLCG1 |  |  |
|  |  |  |  | PLCG2 |  |  |
|  |  |  |  | PLEKHM2 |  |  |
|  |  |  |  | PML |  |  |
|  |  |  |  | PMS1 |  |  |
|  |  |  |  | PMS2 |  |  |
|  |  |  |  | POFUT1 |  |  |
|  |  |  |  | POLD1 |  |  |
|  |  |  |  | POLD4 |  |  |
|  |  |  |  | POLR2H |  |  |
|  |  |  |  | POM121 |  |  |
|  |  |  |  | POMGNT1 |  |  |
|  |  |  |  | POSTN |  |  |
|  |  |  |  | POT1 |  |  |
|  |  |  |  | POU2AF1 |  |  |
|  |  |  |  | POU5F1 |  |  |
|  |  |  |  | PPAP2B |  |  |
|  |  |  |  | PPARG |  |  |
|  |  |  |  | PPARGC1A |  |  |
|  |  |  |  | PPFIA2 |  |  |
|  |  |  |  | PPFIBP1 |  |  |
|  |  |  |  | PPM1D |  |  |
|  |  |  |  | PPP1CB |  |  |
|  |  |  |  | PPP1R13B |  |  |
|  |  |  |  | PPP1R13L |  |  |
|  |  |  |  | PPP2CB |  |  |
|  |  |  |  | PPP2R1A |  |  |
|  |  |  |  | PPP2R1B |  |  |
|  |  |  |  | PPP2R2B |  |  |
|  |  |  |  | PPP2R4 |  |  |
|  |  |  |  | PPP3CA |  |  |
|  |  |  |  | PPP3CB |  |  |
|  |  |  |  | PPP3CC |  |  |
|  |  |  |  | PPP3R1 |  |  |
|  |  |  |  | PPP3R2 |  |  |
|  |  |  |  | PPP4C |  |  |
|  |  |  |  | PQLC3 |  |  |
|  |  |  |  | PRCC |  |  |
|  |  |  |  | PRDM1 |  |  |
|  |  |  |  | PRDM16 |  |  |
|  |  |  |  | PRDM7 |  |  |
|  |  |  |  | PRF1 |  |  |
|  |  |  |  | PRG2 |  |  |
|  |  |  |  | PRICKLE1 |  |  |
|  |  |  |  | PRKACA |  |  |
|  |  |  |  | PRKACG |  |  |
|  |  |  |  | PRKAR1A |  |  |
|  |  |  |  | PRKCA |  |  |
|  |  |  |  | PRKCB |  |  |
|  |  |  |  | PRKCD |  |  |
|  |  |  |  | PRKCG |  |  |
|  |  |  |  | PRKDC |  |  |
|  |  |  |  | PRKG2 |  |  |
|  |  |  |  | PRMT1 |  |  |
|  |  |  |  | PRMT8 |  |  |
|  |  |  |  | PROM1 |  |  |
|  |  |  |  | PRRX1 |  |  |
|  |  |  |  | PRRX2 |  |  |
|  |  |  |  | PRSS8 |  |  |
|  |  |  |  | PSD3 |  |  |
|  |  |  |  | PSEN1 |  |  |
|  |  |  |  | PSIP1 |  |  |
|  |  |  |  | PSMD2 |  |  |
|  |  |  |  | PTBP1 |  |  |
|  |  |  |  | PTCH1 |  |  |
|  |  |  |  | PTCRA |  |  |
|  |  |  |  | PTEN |  |  |
|  |  |  |  | PTGS2 |  |  |
|  |  |  |  | PTK2 |  |  |
|  |  |  |  | PTK2B |  |  |
|  |  |  |  | PTK7 |  |  |
|  |  |  |  | PTPN11 |  |  |
|  |  |  |  | PTPN2 |  |  |
|  |  |  |  | PTPN6 |  |  |
|  |  |  |  | PTPRA |  |  |
|  |  |  |  | PTPRK |  |  |
|  |  |  |  | PTPRO |  |  |
|  |  |  |  | PTPRR |  |  |
|  |  |  |  | PTTG1 |  |  |
|  |  |  |  | PVT1 |  |  |
|  |  |  |  | RABEP1 |  |  |
|  |  |  |  | RAC1 |  |  |
|  |  |  |  | RAC2 |  |  |
|  |  |  |  | RAC3 |  |  |
|  |  |  |  | RAD21 |  |  |
|  |  |  |  | RAD50 |  |  |
|  |  |  |  | RAD51 |  |  |
|  |  |  |  | RAD51B |  |  |
|  |  |  |  | RAD51C |  |  |
|  |  |  |  | RAD51D |  |  |
|  |  |  |  | RAD52 |  |  |
|  |  |  |  | RAF1 |  |  |
|  |  |  |  | RALGDS |  |  |
|  |  |  |  | RANBP17 |  |  |
|  |  |  |  | RANBP2 |  |  |
|  |  |  |  | RAP1GDS1 |  |  |
|  |  |  |  | RARA |  |  |
|  |  |  |  | RASAL1 |  |  |
|  |  |  |  | RASGEF1A |  |  |
|  |  |  |  | RASGRF1 |  |  |
|  |  |  |  | RASGRF2 |  |  |
|  |  |  |  | RASGRP1 |  |  |
|  |  |  |  | RB1 |  |  |
|  |  |  |  | RBM15 |  |  |
|  |  |  |  | RBM6 |  |  |
|  |  |  |  | RCHY1 |  |  |
|  |  |  |  | RCOR1 |  |  |
|  |  |  |  | RCSD1 |  |  |
|  |  |  |  | RECQL4 |  |  |
|  |  |  |  | REEP3 |  |  |
|  |  |  |  | RELA |  |  |
|  |  |  |  | RELN |  |  |
|  |  |  |  | RERG |  |  |
|  |  |  |  | RET |  |  |
|  |  |  |  | RGS7 |  |  |
|  |  |  |  | RHBDF2 |  |  |
|  |  |  |  | RHOA |  |  |
|  |  |  |  | RHOD |  |  |
|  |  |  |  | RHOH |  |  |
|  |  |  |  | RICTOR |  |  |
|  |  |  |  | RLTPR |  |  |
|  |  |  |  | RMI2 |  |  |
|  |  |  |  | RNF213 |  |  |
|  |  |  |  | RNF43 |  |  |
|  |  |  |  | ROBO1 |  |  |
|  |  |  |  | ROBO2 |  |  |
|  |  |  |  | ROS1 |  |  |
|  |  |  |  | RPA3 |  |  |
|  |  |  |  | RPL22 |  |  |
|  |  |  |  | RPN1 |  |  |
|  |  |  |  | RPN2 |  |  |
|  |  |  |  | RPS21 |  |  |
|  |  |  |  | RPS6KA1 |  |  |
|  |  |  |  | RPS6KA2 |  |  |
|  |  |  |  | RPS6KA3 |  |  |
|  |  |  |  | RPTOR |  |  |
|  |  |  |  | RREB1 |  |  |
|  |  |  |  | RRM1 |  |  |
|  |  |  |  | RRM2B |  |  |
|  |  |  |  | RTEL1 |  |  |
|  |  |  |  | RTN3 |  |  |
|  |  |  |  | RUNX1 |  |  |
|  |  |  |  | RUNX1T1 |  |  |
|  |  |  |  | RUNX2 |  |  |
|  |  |  |  | RYR3 |  |  |
|  |  |  |  | S1PR2 |  |  |
|  |  |  |  | SARNP |  |  |
|  |  |  |  | SBDS |  |  |
|  |  |  |  | SCN8A |  |  |
|  |  |  |  | SDC4 |  |  |
|  |  |  |  | SDHA |  |  |
|  |  |  |  | SDHAF2 |  |  |
|  |  |  |  | SDHB |  |  |
|  |  |  |  | SDHC |  |  |
|  |  |  |  | SDHD |  |  |
|  |  |  |  | SEC31A |  |  |
|  |  |  |  | SEPT2 |  |  |
|  |  |  |  | SEPT5 |  |  |
|  |  |  |  | SEPT6 |  |  |
|  |  |  |  | SEPT9 |  |  |
|  |  |  |  | SERP2 |  |  |
|  |  |  |  | SERPINE1 |  |  |
|  |  |  |  | SERPINF1 |  |  |
|  |  |  |  | SET |  |  |
|  |  |  |  | SETBP1 |  |  |
|  |  |  |  | SETD2 |  |  |
|  |  |  |  | SETD7 |  |  |
|  |  |  |  | SF3B1 |  |  |
|  |  |  |  | SFPQ |  |  |
|  |  |  |  | SFRP2 |  |  |
|  |  |  |  | SFRP4 |  |  |
|  |  |  |  | SGK1 |  |  |
|  |  |  |  | SGPP2 |  |  |
|  |  |  |  | SH2D5 |  |  |
|  |  |  |  | SH3BP1 |  |  |
|  |  |  |  | SH3D19 |  |  |
|  |  |  |  | SH3GL1 |  |  |
|  |  |  |  | SH3GL2 |  |  |
|  |  |  |  | SHC1 |  |  |
|  |  |  |  | SHC2 |  |  |
|  |  |  |  | SIK3 |  |  |
|  |  |  |  | SIN3A |  |  |
|  |  |  |  | SIRT1 |  |  |
|  |  |  |  | SKP2 |  |  |
|  |  |  |  | SLC1A2 |  |  |
|  |  |  |  | SLC34A2 |  |  |
|  |  |  |  | SLC45A3 |  |  |
|  |  |  |  | SLC7A5 |  |  |
|  |  |  |  | SLCO1B3 |  |  |
|  |  |  |  | SLX4 |  |  |
|  |  |  |  | SMAD2 |  |  |
|  |  |  |  | SMAD3 |  |  |
|  |  |  |  | SMAD4 |  |  |
|  |  |  |  | SMAD6 |  |  |
|  |  |  |  | SMAP1 |  |  |
|  |  |  |  | SMARCA1 |  |  |
|  |  |  |  | SMARCA4 |  |  |
|  |  |  |  | SMARCA5 |  |  |
|  |  |  |  | SMARCB1 |  |  |
|  |  |  |  | SMC1A |  |  |
|  |  |  |  | SMC3 |  |  |
|  |  |  |  | SMO |  |  |
|  |  |  |  | SNAPC3 |  |  |
|  |  |  |  | SNCG |  |  |
|  |  |  |  | SNHG5 |  |  |
|  |  |  |  | SNW1 |  |  |
|  |  |  |  | SNX29 |  |  |
|  |  |  |  | SNX9 |  |  |
|  |  |  |  | SOCS1 |  |  |
|  |  |  |  | SOCS2 |  |  |
|  |  |  |  | SOCS3 |  |  |
|  |  |  |  | SOD2 |  |  |
|  |  |  |  | SORBS2 |  |  |
|  |  |  |  | SORT1 |  |  |
|  |  |  |  | SOS1 |  |  |
|  |  |  |  | SOX10 |  |  |
|  |  |  |  | SOX11 |  |  |
|  |  |  |  | SOX2 |  |  |
|  |  |  |  | SP1 |  |  |
|  |  |  |  | SP3 |  |  |
|  |  |  |  | SPECC1 |  |  |
|  |  |  |  | SPEN |  |  |
|  |  |  |  | SPOP |  |  |
|  |  |  |  | SPP1 |  |  |
|  |  |  |  | SPRY2 |  |  |
|  |  |  |  | SPRY4 |  |  |
|  |  |  |  | SPTAN1 |  |  |
|  |  |  |  | SPTBN1 |  |  |
|  |  |  |  | SQSTM1 |  |  |
|  |  |  |  | SRC |  |  |
|  |  |  |  | SRF |  |  |
|  |  |  |  | SRGAP3 |  |  |
|  |  |  |  | SRRM3 |  |  |
|  |  |  |  | SRSF2 |  |  |
|  |  |  |  | SRSF3 |  |  |
|  |  |  |  | SS18 |  |  |
|  |  |  |  | SS18L1 |  |  |
|  |  |  |  | SSBP2 |  |  |
|  |  |  |  | SSX1 |  |  |
|  |  |  |  | SSX2 |  |  |
|  |  |  |  | SSX4 |  |  |
|  |  |  |  | ST6GAL1 |  |  |
|  |  |  |  | STAG2 |  |  |
|  |  |  |  | STAT1 |  |  |
|  |  |  |  | STAT3 |  |  |
|  |  |  |  | STAT4 |  |  |
|  |  |  |  | STAT5A |  |  |
|  |  |  |  | STAT5B |  |  |
|  |  |  |  | STAT6 |  |  |
|  |  |  |  | STIL |  |  |
|  |  |  |  | STK11 |  |  |
|  |  |  |  | STL |  |  |
|  |  |  |  | STRN |  |  |
|  |  |  |  | STX5 |  |  |
|  |  |  |  | STYK1 |  |  |
|  |  |  |  | SUFU |  |  |
|  |  |  |  | SUGP2 |  |  |
|  |  |  |  | SULF1 |  |  |
|  |  |  |  | SUV39H2 |  |  |
|  |  |  |  | SUZ12 |  |  |
|  |  |  |  | SYK |  |  |
|  |  |  |  | SYP |  |  |
|  |  |  |  | TACC1 |  |  |
|  |  |  |  | TACC2 |  |  |
|  |  |  |  | TACC3 |  |  |
|  |  |  |  | TAF1 |  |  |
|  |  |  |  | TAF15 |  |  |
|  |  |  |  | TAL1 |  |  |
|  |  |  |  | TAL2 |  |  |
|  |  |  |  | TAOK1 |  |  |
|  |  |  |  | TBL1XR1 |  |  |
|  |  |  |  | TBX15 |  |  |
|  |  |  |  | TCEA1 |  |  |
|  |  |  |  | TCF12 |  |  |
|  |  |  |  | TCF3 |  |  |
|  |  |  |  | TCF7L2 |  |  |
|  |  |  |  | TCL1A |  |  |
|  |  |  |  | TCL6 |  |  |
|  |  |  |  | TCTA |  |  |
|  |  |  |  | TEAD1 |  |  |
|  |  |  |  | TEAD2 |  |  |
|  |  |  |  | TEAD3 |  |  |
|  |  |  |  | TEAD4 |  |  |
|  |  |  |  | TEC |  |  |
|  |  |  |  | TENM1 |  |  |
|  |  |  |  | TERF1 |  |  |
|  |  |  |  | TERF2 |  |  |
|  |  |  |  | TERT |  |  |
|  |  |  |  | TET1 |  |  |
|  |  |  |  | TET2 |  |  |
|  |  |  |  | TFAP2A |  |  |
|  |  |  |  | TFDP1 |  |  |
|  |  |  |  | TFE3 |  |  |
|  |  |  |  | TFEB |  |  |
|  |  |  |  | TFG |  |  |
|  |  |  |  | TFPT |  |  |
|  |  |  |  | TFRC |  |  |
|  |  |  |  | TGFB2 |  |  |
|  |  |  |  | TGFB3 |  |  |
|  |  |  |  | TGFBI |  |  |
|  |  |  |  | TGFBR2 |  |  |
|  |  |  |  | TGFBR3 |  |  |
|  |  |  |  | THADA |  |  |
|  |  |  |  | THBS1 |  |  |
|  |  |  |  | THRAP3 |  |  |
|  |  |  |  | TIAM1 |  |  |
|  |  |  |  | TIRAP |  |  |
|  |  |  |  | TLL2 |  |  |
|  |  |  |  | TLR4 |  |  |
|  |  |  |  | TLX1 |  |  |
|  |  |  |  | TLX3 |  |  |
|  |  |  |  | TMEM127 |  |  |
|  |  |  |  | TMEM230 |  |  |
|  |  |  |  | TMEM30A |  |  |
|  |  |  |  | TMPRSS2 |  |  |
|  |  |  |  | TNC |  |  |
|  |  |  |  | TNF |  |  |
|  |  |  |  | TNFAIP3 |  |  |
|  |  |  |  | TNFRSF10B | |  |
|  |  |  |  | TNFRSF10D | |  |
|  |  |  |  | TNFRSF11A | |  |
|  |  |  |  | TNFRSF14 |  |  |
|  |  |  |  | TNFRSF17 |  |  |
|  |  |  |  | TNFRSF6B |  |  |
|  |  |  |  | TOP1 |  |  |
|  |  |  |  | TOP2A |  |  |
|  |  |  |  | TOP2B |  |  |
|  |  |  |  | TP53 |  |  |
|  |  |  |  | TP53BP1 |  |  |
|  |  |  |  | TP63 |  |  |
|  |  |  |  | TP73 |  |  |
|  |  |  |  | TPD52L2 |  |  |
|  |  |  |  | TPM3 |  |  |
|  |  |  |  | TPM4 |  |  |
|  |  |  |  | TPO |  |  |
|  |  |  |  | TPR |  |  |
|  |  |  |  | TRAF2 |  |  |
|  |  |  |  | TRAF3 |  |  |
|  |  |  |  | TRAF5 |  |  |
|  |  |  |  | TRHDE |  |  |
|  |  |  |  | TRIM24 |  |  |
|  |  |  |  | TRIM27 |  |  |
|  |  |  |  | TRIM33 |  |  |
|  |  |  |  | TRIP11 |  |  |
|  |  |  |  | TRPS1 |  |  |
|  |  |  |  | TSC1 |  |  |
|  |  |  |  | TSC2 |  |  |
|  |  |  |  | TSHR |  |  |
|  |  |  |  | TTK |  |  |
|  |  |  |  | TTL |  |  |
|  |  |  |  | TUSC3 |  |  |
|  |  |  |  | TYK2 |  |  |
|  |  |  |  | TYMS |  |  |
|  |  |  |  | U2AF1 |  |  |
|  |  |  |  | U2AF2 |  |  |
|  |  |  |  | UBE2B |  |  |
|  |  |  |  | UBE2C |  |  |
|  |  |  |  | UFC1 |  |  |
|  |  |  |  | UFM1 |  |  |
|  |  |  |  | USP16 |  |  |
|  |  |  |  | USP42 |  |  |
|  |  |  |  | USP5 |  |  |
|  |  |  |  | USP6 |  |  |
|  |  |  |  | USP7 |  |  |
|  |  |  |  | VCAM1 |  |  |
|  |  |  |  | VEGFA |  |  |
|  |  |  |  | VEGFC |  |  |
|  |  |  |  | VGLL3 |  |  |
|  |  |  |  | VHL |  |  |
|  |  |  |  | VTI1A |  |  |
|  |  |  |  | WASF2 |  |  |
|  |  |  |  | WDFY3 |  |  |
|  |  |  |  | WDR1 |  |  |
|  |  |  |  | WDR18 |  |  |
|  |  |  |  | WDR70 |  |  |
|  |  |  |  | WDR90 |  |  |
|  |  |  |  | WEE1 |  |  |
|  |  |  |  | WHSC1 |  |  |
|  |  |  |  | WHSC1L1 |  |  |
|  |  |  |  | WIF1 |  |  |
|  |  |  |  | WISP3 |  |  |
|  |  |  |  | WNT10A |  |  |
|  |  |  |  | WNT10B |  |  |
|  |  |  |  | WNT11 |  |  |
|  |  |  |  | WNT16 |  |  |
|  |  |  |  | WNT2B |  |  |
|  |  |  |  | WNT3 |  |  |
|  |  |  |  | WNT4 |  |  |
|  |  |  |  | WNT5B |  |  |
|  |  |  |  | WNT6 |  |  |
|  |  |  |  | WNT7B |  |  |
|  |  |  |  | WNT8B |  |  |
|  |  |  |  | WRN |  |  |
|  |  |  |  | WSB1 |  |  |
|  |  |  |  | WT1 |  |  |
|  |  |  |  | WWOX |  |  |
|  |  |  |  | WWTR1 |  |  |
|  |  |  |  | XBP1 |  |  |
|  |  |  |  | XIAP |  |  |
|  |  |  |  | XKR3 |  |  |
|  |  |  |  | XPA |  |  |
|  |  |  |  | XPC |  |  |
|  |  |  |  | XPO1 |  |  |
|  |  |  |  | XRCC6 |  |  |
|  |  |  |  | YAP1 |  |  |
|  |  |  |  | YPEL5 |  |  |
|  |  |  |  | YTHDF2 |  |  |
|  |  |  |  | YWHAE |  |  |
|  |  |  |  | YY1AP1 |  |  |
|  |  |  |  | ZBTB16 |  |  |
|  |  |  |  | ZC3H7A |  |  |
|  |  |  |  | ZC3H7B |  |  |
|  |  |  |  | ZFP64 |  |  |
|  |  |  |  | ZFPM2 |  |  |
|  |  |  |  | ZFYVE19 |  |  |
|  |  |  |  | ZIC2 |  |  |
|  |  |  |  | ZMIZ1 |  |  |
|  |  |  |  | ZMYM2 |  |  |
|  |  |  |  | ZMYM3 |  |  |
|  |  |  |  | ZMYND11 |  |  |
|  |  |  |  | ZNF207 |  |  |
|  |  |  |  | ZNF217 |  |  |
|  |  |  |  | ZNF24 |  |  |
|  |  |  |  | ZNF331 |  |  |
|  |  |  |  | ZNF384 |  |  |
|  |  |  |  | ZNF444 |  |  |
|  |  |  |  | ZNF521 |  |  |
|  |  |  |  | ZNF585B |  |  |
|  |  |  |  | ZNF687 |  |  |
|  |  |  |  | ZNF703 |  |  |
|  |  |  |  | ZRSR2 |  |  |
